# Time-Lapse Analysis of Human Embryonic Stem Cells Reveals Multiple Bottlenecks Restricting Colony Formation and Their Relief upon Culture Adaptation

**DOI:** 10.1016/j.stemcr.2014.05.006

**Published:** 2014-06-12

**Authors:** Ivana Barbaric, Veronica Biga, Paul J. Gokhale, Mark Jones, Dylan Stavish, Adam Glen, Daniel Coca, Peter W. Andrews

**Affiliations:** 1 Centre for Stem Cell Biology, Department of Biomedical Science, The University of Sheffield, Western Bank, Sheffield S10 2TN, UK; 2 Centre for Signal Processing and Complex Systems, Department of Automatic Control and Systems Engineering, The University of Sheffield, Western Bank, Sheffield S10 2TN, UK; 3 Department of Materials Science and Engineering, The University of Sheffield, Western Bank, Sheffield S10 2TN, UK

## Abstract

Using time-lapse imaging, we have identified a series of bottlenecks that restrict growth of early-passage human embryonic stem cells (hESCs) and that are relieved by karyotypically abnormal variants that are selected by prolonged culture. Only a minority of karyotypically normal cells divided after plating, and these were mainly cells in the later stages of cell cycle at the time of plating. Furthermore, the daughter cells showed a continued pattern of cell death after division, so that few formed long-term proliferating colonies. These colony-forming cells showed distinct patterns of cell movement. Increasing cell density enhanced cell movement facilitating cell:cell contact, which resulted in increased proportion of dividing cells and improved survival postplating of normal hESCs. In contrast, most of the karyotypically abnormal cells reentered the cell cycle on plating and gave rise to healthy progeny, without the need for cell:cell contacts and independent of their motility patterns.

## Highlights

• The growth of karyotypically normal hESCs is limited by multiple bottlenecks
• Bottlenecks include initial survival, entry into mitosis, and death following mitosis
• A privileged clonogenic state in normal hESCs is characterized by distinct motility
• Variant adapted cells and ROCK inhibition alleviate identified bottlenecks

## Graphical Abstract

**Figure:**
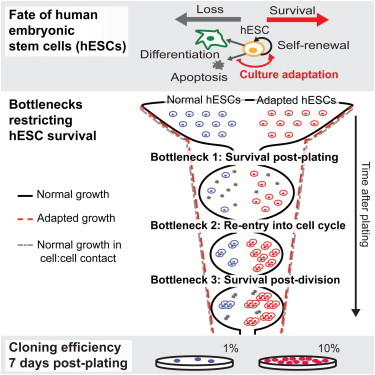

## Teaser

Andrews, Coca, and colleagues use time-lapse imaging of single human embryonic stem cells (hESCs) to identify aspects of their behavior that tend to restrict growth and so would be targets for mutations that provide selective advantage. They identify a series of bottlenecks that restricts growth of early-passage hESCs and that is relieved by karyotypically abnormal variants selected by prolonged culture.

## Introduction

Seemingly at odds with their indefinite self-renewing capability in vitro, human embryonic stem cells (hESCs) display a high death rate in culture, contributing to the problems of efficient mass culture. Indeed, although the cell-cycle time of hESCs is relatively short (less than 24 hr) (Becker et al., 2006), hESCs are commonly passaged only every 4–5 days at low split ratios (1:3 or even 1:2), implying a loss of up to 90% of cells from cultures (Olariu et al., 2010). The extensive hESC death is even further exacerbated upon passaging of cells by enzymatic methods that entail dissociation of cell colonies to single cells (Chen et al., 2010; Ohgushi et al., 2010; Watanabe et al., 2007), resulting in a very low single-cell cloning efficiency (typically <1%) (Enver et al., 2005; Harrison et al., 2007). The low cloning efficiency is at least partly due to an excessive apoptosis of cells upon dissociation (Chen et al., 2010; Ohgushi et al., 2010), but the discrepancy in the number of cells surviving the initial plating and the overall cloning efficiency suggests that critical restriction points exist between initial plating and when robust colony formation is established. The nature of these further restrictions remains unknown, although we have previously posited that cell:cell contact provides crucial signals, perhaps mediated by the NOTCH system, for the survival and proliferation of undifferentiated human pluripotent stem cells (Andrews et al., 1982; Fox et al., 2008).

The severe reduction in hESC numbers during culture has been proposed to impose a strong selection pressure on cells for genetic variants that permit escape from the normal restrictions for self-renewal (Amps et al., 2011; Baker et al., 2007; Draper et al., 2004). Indeed, nonrandom karyotypic changes, which might be indicative of such variants, are frequently observed in hESC cultures (Amps et al., 2011). We have previously termed such karyotypically abnormal hESC culture “adapted” cells because they present substantially more robust population growth patterns (Enver et al., 2005). The issue of adaptation has raised concerns about the safety of hESCs in regenerative therapies and has brought to the forefront the need for detection of adapted cells arising in culture. One of the major hallmarks of adapted cells is improved cloning efficiency (Enver et al., 2005; Harrison et al., 2007), but the interpretation of the origin of differences in cloning efficiencies of normal and adapted cells is complicated by the fact that in the cloning assays as routinely practiced, single cells are scored for their ability to form colonies after several days of growth, and no account is usually taken of how different sublineages from the single founder cells contribute to the final colony. Here, we have used a combination of cell sorting, time-lapse video microscopy, single-cell tracking, and modeling techniques to characterize the bottlenecks that prevent clonal expansion of normal hESCs and elucidate how these are overcome by adapted cells.

## Results

### Bottlenecks in Postplating Survival and Re-entry into the Cell Cycle Are Relieved upon Culture Adaptation

**Figure 1:**
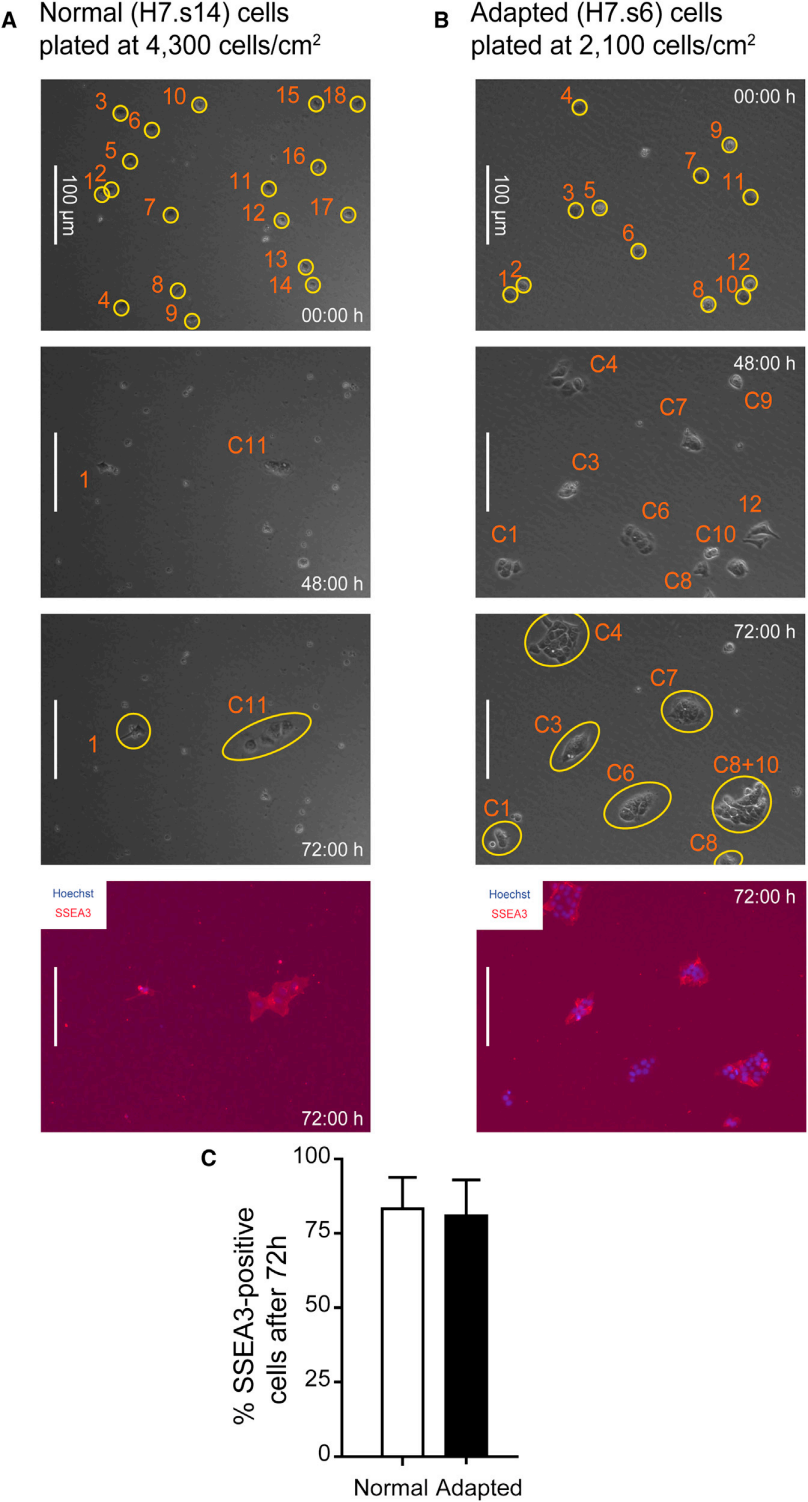
Single-Cell Behavior of Normal H7.s14 and Adapted H7.s6 Cells Investigated by Time-Lapse Analysis (A) Time-lapse analysis of SSEA3-sorted normal cells plated at low density. Still images are taken from Movie S1. Cells labeled 1–18 are circled in the uppermost panel, and their corresponding lineage trees are shown in Figure 2A. The only colony present at 72 hr is labeled C11 (originating from cell 11). The lowermost panel shows staining for SSEA3 (red) and nuclei counterstained with Hoechst 33342 (blue) at the end of the filming. (B) Time-lapse analysis of SSEA3-sorted adapted cells plated at low density. Still images are taken from Movie S2. Cells labeled 1–12 are circled in the uppermost panel, and their corresponding lineage trees are shown in Figure 2B. The majority of colonies present at 72 hr were formed from single cells (labeled C1–C8 according to the originating cell), but occasionally, we noted satellite colonies being formed (cell 8 gives rise to progeny in two colonies labeled C8 and C8+10) as well as rare mixing of nearby colonies (progeny from cells 8 and 10 found in the mixed colony labeled C8+10). The resulting colonies were stained for SSEA3 (red) and nuclei counterstained with Hoechst 33342 (blue) at the end of the filming (lowermost panel). (C) Proportions of normal and adapted cells positive for SSEA3 at the end of the time-lapse experiments (72 hr). Results are the mean of triplicate independent experiments ± SD.

We compared the behavior of normal and adapted sublines of two well-characterized hESC lines, H7 and H14 (Thomson et al., 1998): H7.s14 and H14.s9 are karyotypically normal sublines, whereas H7.s6 and H14.BJ1 are later passage, adapted sublines, with karyotypic changes and a markedly increased population growth rate (Baker et al., 2007; Draper et al., 2004; Enver et al., 2005). To ensure that the time-lapse analysis was done with undifferentiated stem cells rather than their differentiated derivatives, we first sorted the cells on the basis of their expression of the cell surface antigen, SSEA3, a particularly sensitive marker of the undifferentiated state (Draper et al., 2002; Enver et al., 2005). Dissociated normal and adapted cells were plated at low density and filmed from the time of plating for 72 hr (Figures 1A and 1B; Movies S1 and S2 available online). At the end of 72 hr, cells were fixed and stained for SSEA3 (using a different secondary antibody to the initial sort) to determine whether they remained undifferentiated (Figures 1A and 1B). A majority of cells remained SSEA3 positive, and there was no difference in the proportion of cells that expressed SSEA3 between normal and adapted cells (Figure 1C).

**Figure 2:**
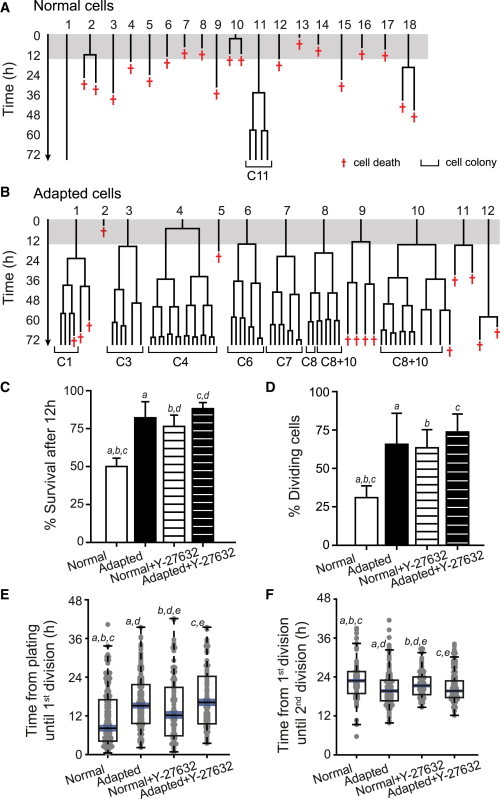
Bottlenecks Limiting the Growth of Normal H7.s14 hESCs Revealed through Time-Lapse Imaging (A) Lineage trees of normal cells reconstructed from Movie S1 filmed over 72 hr from the time of plating the cells. Numbers correspond to cells circled in Figure 1A. Red crosses indicate the cell death. Label C11 denotes a colony arising from cell 11, which is also highlighted in Figure 1A. Gray-shaded area indicates the first 12 hr postplating. (B) Lineage trees of adapted (H7.s6) cells reconstructed from Movie S2 filmed over 72 hr from the time of plating the cells. Numbers correspond to cells circled in Figure 1B. Red crosses indicate the cell death. Labels C1–C8 denote clonal colonies, whereas label C8+10 denotes a colony arising from merged progeny of cells 8 and 10, also highlighted in Figure 1B. Gray-shaded area indicates the first 12 hr postplating. (C) The percentage of cells from the initial plated population that survived the first 12 hr after plating for normal, adapted, and Y-27632-treated normal and adapted cells. Results are the mean of quadruplicate independent experiments ± SD (a–d represent Student’s t tests, left tail; p values: a, <0.001; b, <0.001; c, <0.0001; d, <0.05). Data consist of 150–205 cells per culture. (D) The percentage of cells that divided in the normal, adapted, and Y-27632-treated normal and adapted cultures plated at low density. Results are the mean of quadruplicate independent experiments ± SD (a–c represent Student’s t tests, left tail; p values: a, <0.01; b, <0.01; c, <0.001). Data consist of 150–205 cells per culture. (E) The cell-cycle re-entry time following plating for normal, adapted, and Y-27632-treated normal and adapted cells. Results are box plot representations of distributions from a minimum of four independent experiments with median line and 95% median confidence intervals (blue); observations (gray dots) are shown stretching horizontal by frequency (a–e represent Kruskal-Wallis tests; p values: a, <10^−8^; b, <0.01; c, <10^−8^; d, <0.05; e, <0.01). Data consist of 110–170 cells per culture. (F) Cell-cycle time in subsequent divisions for normal, adapted, and Y-27632-treated normal and adapted cells. Results are box plot representations of distributions from a minimum of four independent experiments with median line and 95% median confidence intervals (blue); observations (gray dots) are shown stretching horizontal by frequency (a–e represent Kruskal-Wallis tests; p values: a, <0.0001; b, <0.05; c, <0.001; d, <0.05; e, <0.001). Data consist of 70–170 cells per culture.

Cells were tracked from movies to record a detailed history of their survival and mitoses (Figures 2A and 2B; Movies S1 and S2). The lineage trees reconstructed from time-lapse movies of normal and adapted H7 cells were markedly different (Figures 2A and 2B). Strikingly, adapted cells showed better recovery after plating, with 82% ± 11% of the initial population still alive after 12 hr (Figure 2C) in contrast to 50% ± 5% of the initial normal population. The Rho-associated, coiled-coil containing protein kinase 1 (ROCK) inhibitor Y-27632 (Watanabe et al., 2007) improved the survival of normal cells but had no effect on the survival of adapted cells after 12 hr (Figures 2C and S1A–S1D; Movies S3 and S4). Although Y-27632 had no apparent beneficial effects on the individual adapted cells tracked in time-lapse movies, it increased the number of attached cells after plating and hence the overall cell number at 24 hr postplating (Figure S1E). We observed similar differences in the survival after plating between normal and adapted H14 hESCs (Figure S2A).

**Figure 3:**
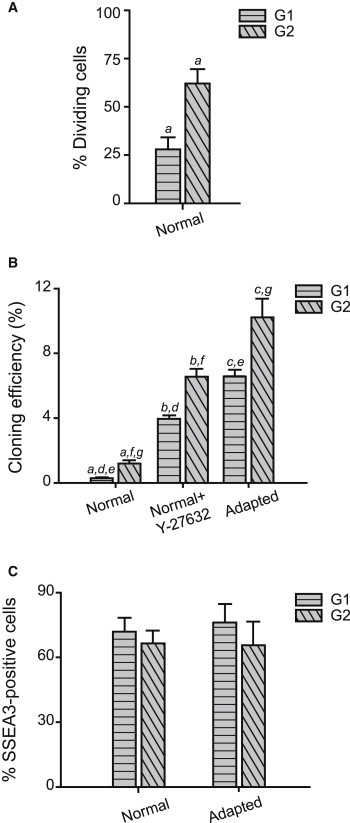
Cloning Efficiencies of Normal H7.s14 and Adapted H7.s6 Cells Plated in G1 or G2 Cell-Cycle Phase (A) The percentage of cells that divided in the normal SSEA3-sorted population fractionated into G1 and G2 based on the DNA profile, plated at low density, and analyzed using time-lapse imaging. Results are the mean of triplicate independent experiments ± SD (a represents Student’s t test, left tail; p value: <0.01). Data consist of 160–320 cells per fraction. (B) A representative example of cloning efficiencies of SSEA3-sorted normal and adapted cells fractionated into G1 and G2 based on the DNA profile, analyzed 7 days after plating. Results are the mean of triplicate wells from the same experiment ± SD (a–g represent Student’s t tests, left tail; p values: a, <10^−5^; b, <0.001; c, <0.01; d, <10^−9^; e, <10^−8^; f, <10^−6^; g, <10^−6^). (C) Proportions of normal and adapted cells expressing SSEA3 7 days after plating SSEA3-sorted cells fractionated into G1 and G2 based on the DNA profile. Results are the mean of triplicate wells from the same experiment ± SD.

Of the cells that survived plating, a smaller proportion of the normal than the adapted H7 hESCs reentered the cell cycle and divided (31% ± 8% in normal cells compared to 66% ± 20% in adapted cells) (Figure 2D), indicating a second bottleneck in the expansion of the normal cells. Normal and adapted H14 cells displayed the same trend in reentry into the cell cycle (Figure S2B). This second bottleneck was overcome by treatment of normal cells with the ROCK inhibitor, Y-27632 (Figure 2D). However, the time from plating to the first division was shorter for the surviving, mitotic normal cells (median 8 hr) than for the adapted cells (median 15 hr), or for the normal or adapted cells treated with Y-27632 (medians 12 and 16 hr, respectively) (Figure 2E). H14 normal and adapted cells displayed a similar pattern (Figure S2C). On the other hand, in subsequent cell divisions, the cell-cycle times for the normal and adapted cells, whether or not exposed to Y-27632, were similar, although there were small differences (Figures 2F and S2D). One explanation for the difference in the time until the first division would be that only normal cells that are in the later stages of the cell cycle are able to divide after plating. Indeed, we conducted experiments with cells sorted based on SSEA3 expression and DNA content to show that the majority of dividing normal cells come from the G2 fraction (Figure 3A). However, the same appeared to be true for the adapted cells (data not shown), and in cloning efficiency experiments, the G2 cells were more efficient in creating colonies than G1 in both sublines: the cloning efficiency ratio of G2/G1 in triplicate experiments was 3.1 ± 0.9 in normal cells and 1.8 ± 0.6 in adapted cells (no statistical difference) (Figure 3B). SSEA3 staining at the end of the cloning assay revealed no difference in the percentage of undifferentiated cells between colonies formed from cells in G1 or G2 at the time of plating (Figure 3C), indicating that in the conditions we used, the G1 cells are more sensitive to death (rather than differentiation) signals upon plating than the G2 cells. Furthermore, no significant difference between the ratios of G2/G1 cloning efficiencies in normal and adapted cells suggests that the difference in time to first division between normal and adapted cells is not due to stage in the cell cycle but rather to the normal cells reentering cell cycle prematurely before they had recovered from the disruption of harvesting and reseeding. Evidently, ROCK inhibitor is able to prevent this premature re-entry into cell division (Figure 3B). We confirmed that the observed bottlenecks were not a result of the change in culture conditions (i.e., transferring cells from mouse embryonic fibroblasts to Matrigel), by analyzing survival of cells that were serially passaged in Matrigel with mTeSR for three to four passages and then filming them under the same culture conditions (Figures S3A and 3B).

### Cell Death Postdivision Further Limits the Clonal Expansion of Normal hESCs but Is Reduced upon Culture Adaptation

**Figure 4:**
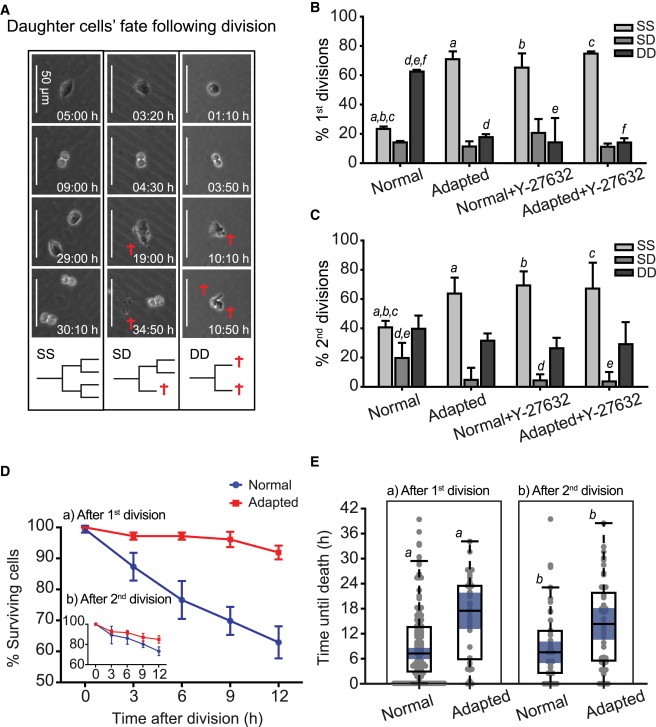
Survival of Normal H7.s14 and Adapted H7.s6 Daughter Cells Postdivision Characterized through Time-Lapse Imaging (A) Possible scenarios of daughter cells’ fate following division: (1) both daughter cells divide (SS), (2) only one daughter cell divides (SD), and (3) both daughter cells die without dividing (DD). Still images from time-lapse movies exemplify the three scenarios. Red crosses indicate cell death. Time after plating is indicated in each of the still images. (B) Percentage of SS, SD, and DD types of division occurring in normal, adapted, and Y-27632-treated normal and adapted cells that divided for the first time after plating. Results are the mean of triplicate independent experiments ± SD (a–f represent Student’s t tests, a–c left tail, d–f right tail; p values: a, 0.0001; b, <0.001; c, <10^−5^; d, <10^−5^; e, <0.01; f, <0.0001). Data consist of 60–110 cell divisions per culture. (C) Percentage of SS, SD, and DD types of division occurring in normal, adapted, and Y-27632-treated normal and adapted cells that divided for the second time after plating. Results are the mean of triplicate independent experiments ± SD (a–d represent Student’s t tests, a–c left tail, d–e right tail; p values: a, <0.05; b, <0.01; c, <0.05; d, <0.05; e, <0.05). Data consist of 35–65 cell divisions per culture. (D) Percentage of surviving normal and adapted cells over a 12 hr time period following the first (a) and the second division postplating (b). Results are the mean of triplicate independent experiments ± SD. Data consist of 145–250 cells per culture. (E) The time from division until the observed cell death in time-lapse movies of normal and adapted cells, corresponding to the first (a) and the second division (b) postplating. Results are box plot representations of distributions from triplicate independent experiments with median line and 95% median confidence intervals (blue); observations (gray dots) are shown stretching horizontal by frequency (a and b represent Kruskal-Wallis tests; p values: a, <0.0001; b, <0.01). Data consist of 40–160 cells per culture.

Another factor that can influence the population growth and colony formation is the rate of cell loss during subsequent culture. Following division, daughter cells can conform to one of the three patterns: both daughter cells survive and give progeny (SS, survival-survival); one daughter cell survives and gives progeny, the other one dies (SD, survival-death); or both daughter cells die without dividing (DD, death-death) (Figure 4A). Although all three scenarios occurred during the regrowth of normal and adapted cells, normal cell divisions were strongly biased toward both daughter cells dying following the first division postplating (DD, 62% ± 1%) compared to both adapted daughter cells surviving (SS, 71% ± 5%) (Figure 4B). A similar trend was identified in the H14 cell pair (Figure S2E) and in H7 cells after serial passaging on Matrigel (Figure S3C). Following the second division, the proportion of divisions leading to both daughter cells dying was reduced in normal cells compared to the first division postplating but, nonetheless, remained high (DD, 40% ± 9%), demonstrating that persistent cell death is an inherent feature of hESC cultures but is reduced by culture adaptation (Figures 4B and 4C). Treatment of normal cells with the ROCK inhibitor Y-27632 shifted the bias in cell division from death (DD) to survival (SS) of both daughter cells, with no further improvement in Y-27632-treated adapted cultures (Figures 4B and 4C). The number of surviving normal daughter cells declined rapidly in the 12 hr following the first division (Figure 4Da), with death occurring relatively early after the division (median 8 hr) (Figure 4Ea). Although the survival of normal daughter cells was improved following the second division (Figure 4Db), daughter cells continued to die sooner following division compared to the adapted cells (Figure 4Eb).

### Direct Cell:Cell Contact Rather Than Diffusible Factors Enhances Survival and Re-entry into the Cell Cycle of Normal hESCs

**Figure 5:**
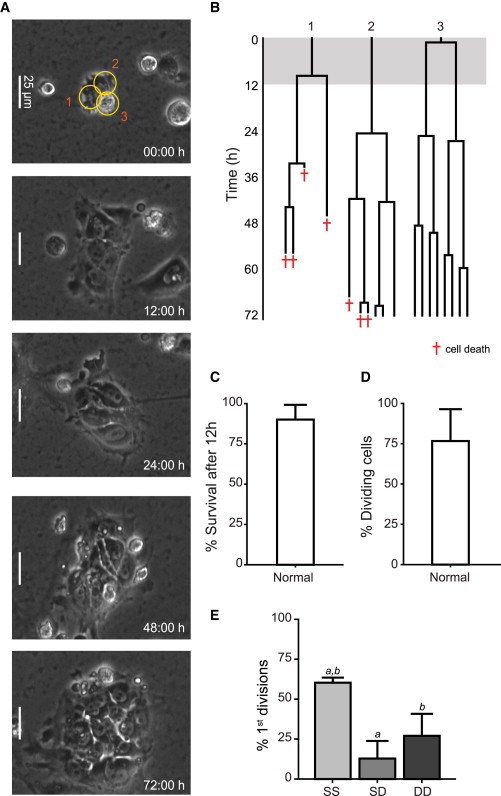
Time-Lapse Analysis of Normal H7.s14 Cells Passaged in Small Clumps (A) A three-cell clump of normal cells obtained by dissociation of cells with nonenzymatic solution. Still images are taken from Movie S5. The cells in the clump are labeled 1–3 and circled in the uppermost panel, and their corresponding lineage trees are shown in (B). (B) Lineage trees of normal cells passaged in small clumps (reconstructed from Movie S5) filmed over 72 hr from the time of plating the cells. Numbers correspond to cells circled in (A). Red crosses indicate the cell death. (C) The percentage of normal cells passaged as clumps that survived the first 12 hr postplating. Results are the mean of triplicate wells from the same experiment ± SD. Data consist of approximately 40 cells. (D) The percentage of normal cells passaged as clumps that divided after plating. Results are the mean of triplicate wells from the same experiment ± SD. Data consist of approximately 40 cells. (E) Percentage of first divisions following which both daughter cells divide further (SS), one daughter cell divides whereas the other dies (SD), or both daughter cells die (DD) in normal cells passaged as clumps. Results are the mean of triplicate wells from the same experiment ± SD (a and b represent Student’s t tests, right tail; p values: a, 0.001; b, <0.01). Data consist of approximately 25 divisions.

**Figure 6:**
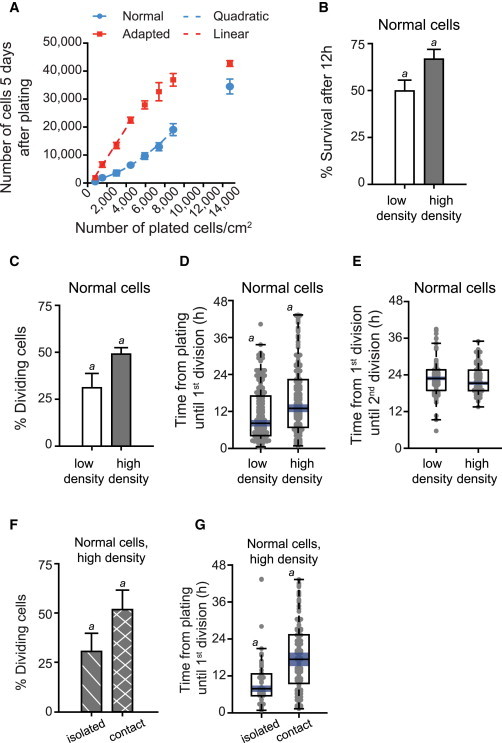
The Effect of High-Plating Density on the Survival of Normal H7.s14 hESCs (A) Effect of increasing cell-plating density on the numbers of normal (H7.s14) and adapted (H7.s6) cells 5 days postplating. The dashed blue line indicates a quadratic trend of the normal curve, and the dashed red line indicates the linear trend of the adapted curve. Due to the limited area of the well, at high-plating densities, cells form a monolayer, and the growth of the adapted population plateaus. Results are the mean of six wells from the same experiment ± SD. (B) Percentage of surviving normal hESCs 12 hr postplating at low and high cell-plating density. Low-density normal data are also presented in Figure 2C. Results are the mean of a minimum of triplicate independent experiments ± SD (a, Student’s t test, left tail; p value: <0.01). Data consist of 205–365 cells per culture condition. (C) Percentage of normal cells that divided from the initial plated population at low and high cell-plating density. Low-density normal data are also presented in Figure 2D. Results are the mean of a minimum of triplicate independent experiments ± SD (a, Student’s t test, left tail; p value: <0.01). Data consist of 205–365 cells per culture condition. (D) The cell-cycle re-entry time following plating for normal cells plated at low and high cell-plating density. Low-density normal data are also presented in Figure 2E. Results are a box plot analysis of a minimum of three independent experiments with median and 95% median confidence bounds (blue) and observations (gray dots) shown stretching horizontal by frequency (a, Kruskal-Wallis test; p value: <0.0001). Data consist of 175–180 cell divisions per culture condition. (E) Cell-cycle time for normal cells plated at low and high cell-plating density. Low-density normal data are also presented in Figure 2F. Results are box plot representations of distributions from a minimum of triplicate independent experiments with median line and 95% median confidence intervals (blue); observations (gray dots) are shown stretching horizontal by frequency. Data consist of 70–85 cells per culture condition. (F) Percentage of normal cells that divided when present in culture as isolated cells (and without ever making contact with other cells after plating) versus the cells that divided after having come into direct contact with other cells, analyzed within the same culture. Results are the mean of triplicate independent experiments ± SD (a, Student’s t test; p value: <0.05). Data consist of 365 cells. (G) Cell-cycle re-entry postplating for normal cells that divided when present as isolated cells versus the cells that divided after having come into direct contact with other cells, analyzed within the same high-cell density culture. Results are box plot representations of distributions from triplicate independent experiments with median line and 95% median confidence intervals (blue); observations (gray dots) are shown stretching horizontal by frequency (a, Kruskal-Wallis test; p value: <10^−7^). Data consist of 45–130 cells per category.

High-cell density is known to enhance hESC viability, and hESCs are routinely passaged as clumps that increase their survival (Figures 5A–5C; Movie S5), cell-cycle re-entry (Figure 5D; Movie S5), and survival after division (Figure 5E; Movie S5) compared to plating of single cells. However, it remains unclear if high-cell density plays a role in culture adaptation and whether it is mediated by direct cell:cell contact or a diffusible factor produced by the cells. When normal and adapted cells were plated at increasing cell densities, the adapted cells gave rise, as expected, to larger populations compared to the normal cells at all plating densities after 5 days of growth (Figure 6A). However, whereas the adapted cells showed a linear increase in cell numbers in respect to the plating density, the normal cells exhibited a nonlinear (quadratic) relationship between cell-plating density and population growth. Thus, increasing cell-seeding density enhanced the growth of normal cells, but not that of the adapted ones.

Given the growth advantage that high density conferred on normal cells, we used time-lapse microscopy to examine whether it alleviated bottlenecks early after plating. Higher plating density of SSEA3-sorted single cells did improve the initial survival of normal hESCs (Figure 6B) and also increased the proportion of cells that reentered the cell cycle (Figure 6C). Additionally, the bias in cells dividing early after plating evident in the low-density cultures of normal cells was not present at high density (Figure 6D). The cell-cycle duration in subsequent divisions was similar in both plating density conditions (Figure 6E).

Overcoming the bottlenecks by plating of cells at a higher density could be due to prosurvival signals transmitted via cell:cell contacts or due to higher concentrations of diffusible prosurvival factors produced by the cells. At our chosen high-plating density (10,600 cells/cm^2^), not all cells came into contact with others, allowing us to compare the survival and re-entry into the cell cycle of cells that remained isolated as opposed to cells that came into contact with other cells. Strikingly, the normal cells that made contact with other cells were more likely to divide than the isolated cells, which showed similar behavior to cells plated at a low density (Figure 6F). Nevertheless, some isolated cells did divide, but similar to low-density cultures, their time until the first division was low (median 8 hr), in contrast to cells in contact for which the median time to division was approximately 17 hr postplating (Figure 6G), closer to that of the adapted cells. Given that the behavior of both groups of cells was analyzed in the same culture, these results are consistent with the hypothesis that direct cell:cell contact rather than factors present throughout the culture environment promotes survival and mitosis of normal cells.

### The Proliferative Fraction of Normal but Not Adapted Cells Is Solely Associated with Enhanced Motility

Because cell:cell contact benefits normal cells, the growth advantage of the adapted cells could be explained if they were to exhibit enhanced motility. To test this, we analyzed the cell movement in the normal and adapted H7 and H14 cultures before division (<12 hr postplating) by calculating the mean squared displacement (msd) with respect to time (t). The msd is a measure related to the average distance traveled by cells and is proportional to ∼t^β^ in the long time limit (Dieterich et al., 2008), where the parameter 0 < β ≤ 2 distinguishes between different stochastic diffusion processes. For a random walk, or Brownian-like motion, msd has a linear trend, with β = 1, whereas non-Brownian motion types are defined when msd deviates from linearity, i.e., β≠1 (Metzler and Klafter, 2000). The motility analysis of plated normal and adapted hESCs highlighted the existence of three subpopulations, exhibiting different dynamics: (1) Brownian-like cells in which the trajectories resembled a random walk (msd∼t); (2) superdiffusive cells (msd∼t^β^,β > 1) in which the movement of cells was nonrandom; and (3) subdiffusive cells (msd∼t^β^,β < 1), which were essentially nonmotile (Movie S6).

**Figure 7:**
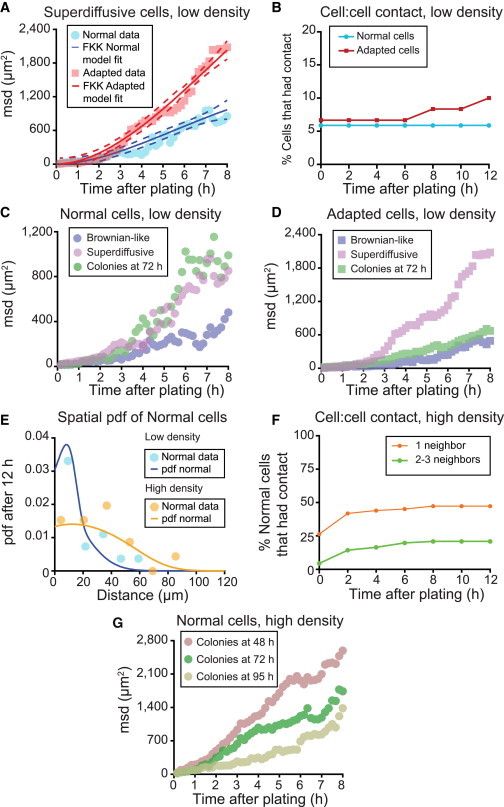
Motility Analysis for Normal H7.s14 and Adapted H7.s6 Cells (A) msd for trajectories of superdiffusive normal and adapted cells at low-plating density. Only motile cells were analyzed, with the msd for Brownian-like cells included in Figure S4A. Markers represent ensemble average msd, solid lines are trends predicted with the FKK model corresponding to superdiffusive motion, and dashed lines indicate the 95% prediction bounds. The rise in the msd for adapted superdiffusive cells indicates an increased ability to travel in culture compared to the trend in normal superdiffusive cells. Data consist of 11–13 cells per culture. (B) Percentage of cells plated at low density that had come into direct cell:cell contact with at least one other cell at any time up to 12 hr postplating. Data consist of 65 cells per culture. (C) Msd calculated from trajectories of normal cells exhibiting Brownian-like (also presented in Figure S4A) and superdiffusive (also presented in A) movement. Markers represent ensemble average msd from real data in specified categories. (D) Msd calculated from trajectories of adapted cells exhibiting Brownian-like (also presented in Figure S4A) and superdiffusive (also presented in A) movement. Markers represent ensemble average msd from real data in specified categories. Data consist of 55 cells forming colonies. (E) Probability of normal cells plated at low and high density to travel any given distance from origin after 12 hr postplating calculated as spatial pdf. Markers represent empirical pdf of distance traveled from origin after 12 hr, and solid lines represent predicted trend of spatial pdf. The trend is calculated assuming an equally weighted mixture of Brownian-like and superdiffusive normal cells. Data consist of approximately 20 cell displacements per culture condition. (F) Percentage of normal cells plated at high density that had come into cell:cell contact with nearby cells at any time up to 12 hr postplating, arranged by number of neighbors. Data consist of 91 cells. (G) Msd calculated from trajectories of normal cells that gave rise to colonies present at 48, 72, and 95 hr postplating. Markers represent ensemble average msd from real data in specified categories. Data consist of 30–80 cells per category.

We analyzed the msd data of the motile cells, both superdiffusive and Brownian-like, by estimating fractional diffusion models. There were no notable differences between the parameters of the diffusion models estimated from trajectories of Brownian-like normal and adapted cells (Figures S4A and S4B; Tables S1 and S2). However, for the adapted superdiffusive cells, the diffusion coefficient was double that of normal superdiffusive cells (Tables S1 and S2), indicating that they tended to travel further from their point of origin (Figures 7A and S4C). Nevertheless, at low-plating density, this made little difference because the average cell:cell distance was approximately 142 μm (Figure S5A), and 95% of normal and adapted cells, whether Brownian-like or superdiffusive, traveled at most only up to 43 μm during 12 hr postplating. Indeed, observations from time-lapse imaging showed that none of the normal and only 3% of adapted cells acquired additional cell contacts through movement over the 12 hr postplating period (Figure 7B), so that the enhanced growth of the adapted cells appears not to be a result of increased cell contacts mediated by enhanced motility. The small increase in contact observed in the adapted culture after 12 hr postplating is due to 95% of superdiffusive adapted hESCs traveling up to 12 μm further than 95% of superdiffusive normal hESCs.

Despite the lack of additional cell contact through cell movement, the msd for the normal cells that gave rise to colonies present at 72 hr postplating closely matched that of superdiffusive normal cells (Figures 7C and S4D). This suggests that the enhanced ability of superdiffusive normal hESCs to proliferate is an intrinsic property of this subpopulation rather than a consequence of increased motility mediating contacts with other cells. In contrast, the msd characteristic of colony-forming adapted cells was intermediate, indicating that, in this case, colonies formed from both Brownian-like and superdiffusive cells (Figures 7D and S4E). Notably, in both the normal and adapted cultures, colonies did not form from nonmotile cells.

Motility may reflect a response to diffusible signaling factors. Because the proliferation of normal cells was promoted by high-cell density conditions, where the concentrations of diffusible factors produced by the cells would be expected to be higher, we investigated whether these conditions further enhanced cell movement. When we analyzed normal H7 cells plated at high density, the msd of both Brownian-like and superdiffusive cells increased compared to the low-density condition (Figure S4F). However, the increased density disproportionately enhanced the motility of superdiffusive cells (diffusion coefficient increased 5-fold, and thermal speed increased 2-fold) compared to Brownian-like cells (diffusion coefficient increased 2-fold, and thermal speed was unchanged) (Table S1). This suggests that cultures do produce factors that enhance the motility of cells and that superdiffusive normal cells are more responsive to such factors.

The probability density function (pdf) derived from the diffusion models shows that at high-plating density, normal cells traveled further from their origins compared to low-density conditions (Figure 7E). Specifically, at high density, the average cell:cell distance was reduced to approximately 47 μm, and 95% of normal cells, whether Brownian-like or superdiffusive, traveled up to 76 μm over 12 hr postplating (Figure S5B). As a result, a significant proportion of normal (either superdiffusive or Brownian-like) cells acquired contacts through motility (Figure 7F). Analyzing the msd curves for cells that gave rise to colonies present in the high-density normal culture revealed that whereas the contribution of superdiffusive cells was noticeable in early colonies (after 48 hr, Figure 7G), at later times, the colony-forming cells encompassed larger proportions of the slower Brownian-like cells (after 72 and 95 hr, Figure 7G). Thus, in addition to the survival of superdiffusive normal cells, high-plating density also allowed Brownian-like normal hESCs to be rescued through contact. A further consequence was that, at high-plating density, most of the colonies derived from the normal cells observed after 12 hr were from mixed lineages rather than clonal (Figure S5B; Movie S7). These observations suggest that the cell density-dependent growth of normal cells is related, at least in part, to their ability to form cell contacts through increased cell motility, a feature not required of adapted cells.

## Discussion

The maintenance and expansion of hESCs are compromised by their propensity to differentiate or to die, which inevitably affords conditions for the selection of genetic variants that provide a growth advantage by altering the cellular dynamics to favor self-renewal. Characterization of the selection pressures that drive the appearance of (epi)genetic variants of hESCs is pivotal for optimizing their maintenance conditions and minimizing the opportunity for expansion of mutated clones that could compromise safety and efficacy for applications in regenerative medicine. Several genetic changes, notably gains of sections of chromosomes 12, 17, and 20, do occur commonly on the long-term passaging of hESCs and appear to provide such growth advantages, seemingly independently of one another. One hypothesis is that overexpression of a gene(s) encoded by these regions of genomic gain provides the selective advantage, but so far, only the role of one such gene, *BCL2L1*, encoded on chromosome 20 has been definitively characterized (Avery et al., 2013), though other candidates have been proposed (Amps et al., 2011; Laurent et al., 2011). In the present study, we took a different approach by using time-lapse analysis of single cells to identify aspects of hESC behavior that tend to restrict growth and so would be targets for mutations that promote proliferation.

In the time-lapse data, only a very small proportion (approximately 3%) of the initially seeded, karyotypically normal cells, but a much greater proportion (approximately 19%) of the karyotypically abnormal, adapted cells, formed robust, continually growing colonies, which correlates well with previous observations of the differential plating efficiencies of normal and adapted hESCs (Enver et al., 2005; Harrison et al., 2007). Our results indicated at least three different bottlenecks to establishing proliferating cultures of hESCs after passaging, namely, initial survival of harvested and replated cells, their subsequent ability to reenter the cell cycle, and, most surprisingly, a continued propensity in those that do reenter the cell cycle to continue to die following subsequent cell divisions. All of these bottlenecks were alleviated in large measure in the adapted, genetically variant sublines of two independent hESC lines: H7 and H14.

Inhibition of ROCK, using the chemical inhibitor Y-27632, also alleviated each of the bottlenecks in the normal cells to a significant extent. Several studies have shown that the tendency of hESCs to die after dissociation to single cells is caused by actin-myosin contraction, mediated by nonmuscle myosin II (Chen et al., 2010; Ohgushi et al., 2010; Walker et al., 2010). ROCK mediates this contraction through phosphorylation of a number of target proteins, notably the myosin regulatory light chains (MLC2) of nonmuscle myosin II. Thus, chemical inhibition of ROCK isoforms ROCK I and II (e.g., with Y-27632, HA1007, or Pinacidil) (Barbaric et al., 2010; Watanabe et al., 2007), or their knockdown, allows cells to reattach to extracellular matrix and survive (Chen et al., 2010; Ohgushi et al., 2010). Our present data from time-lapse tracking of single cells treated with Y-27632 confirmed this, but we also found that the effects of ROCK inhibition extend beyond the rescue of cells at the time of plating. Specifically, the treatment of normal cells with Y-27632 increased the proportion of dividing cells among those that survived reseeding. Furthermore, Y-27632 reverted the apparent premature entry of normal cells into mitosis following replating. The signal(s) that drives normal cells to attempt early divisions is not clear, but it is likely that such a signal is triggered as a result of an inappropriate disruption of extracellular receptors by trypsin treatment during cell dissociation. One possibility is NOTCH receptors, which may regulate hESC proliferation and differentiation (Fox et al., 2008) and are implicated in cell-cycle progression of various cell types (Campa et al., 2008; Joshi et al., 2009), are activated by EDTA-trypsin treatment of cells (Rand et al., 2000). Furthermore, NOTCH activation has been observed to upregulate ROCK1 signaling (Yugawa et al., 2013), and therefore, inhibition of Rho-ROCK signaling could counteract inappropriate activation of NOTCH signaling during dissociation and replating of hESCs. Y-27632 also shifted the bias in cell division from both daughter cells dying after division to both daughter cells surviving, so that normal cells grown in the presence of Y-27632 formed many more robust long-term colonies like the adapted cells. Because completion of cell division requires transient detachment of cells from the extracellular matrix, and given the sensitivity of hESCs to dissociation-related apoptosis (Chen et al., 2010; Ohgushi et al., 2010), one possibility is that the same mechanism that results in the death of normal cells following dissociation might also trigger their death when the cells detach to complete division.

Given the importance of ROCK signaling in hESC survival, it is likely that mutations affecting the activity or expression of multiple genes in this pathway could confer a competitive advantage to mutant cells. Indeed, neither of the adapted sublines of H7 or H14 was significantly influenced at any of the bottlenecks by Y-27632, whereas treatment of the normal sublines with Y-27632 closely phenocopied the adapted cells in each case, suggesting that adaptation in these H7 and H14 sublines may involve mutations affecting the ROCK pathway. A number of proteins that participate in the actin-myosin contractility are encoded by genes on commonly amplified chromosomes in hESCs. However, although these two sublines have some overt karyotypic abnormalities in common, notably gains of the long arm of chromosome 17, they almost certainly have other genetic and epigenetic changes that are not apparent at the gross G-banded karyotype level (Amps et al., 2011; Baker et al., 2007; Enver et al., 2005), so that their ability to overcome these bottlenecks cannot, at this stage, be ascribed to altered expression of genes specifically encoded in those shared regions.

Previous studies have indicated that the maintenance of undifferentiated hESCs is facilitated by culture at high-cell densities (Andrews et al., 1982; Thomson et al., 1998), and we have suggested that this depends upon NOTCH signaling requiring cell:cell contact (Fox et al., 2008). Our present time-lapse data do indeed confirm that the proliferation of normal hESCs is promoted by cell:cell contact. However, these data also reveal that the relationship between hESC proliferation and cell density involves a complex interplay with cell motility, which can be influenced by diffusible factors. Cells can acquire contact either through dividing and forming a colony with a sister cell or by migrating to form a colony with a nonsister cell. At low-plating density, which was prohibitive of cells meeting through migration, cells relied on mitosis for creating contacts. Nevertheless, the rare normal hESCs that did survive and proliferate under these conditions also belonged to a privileged clonogenic state that was characterized by superdiffusive motility; the Brownian-like cells did not seem to contribute to colonies. In the adapted cells, clonogenic capacity was also associated with motility, although in this case, both superdiffusive and Brownian-like cells formed colonies. The absence of nonmotile cells from the subsets of colony-forming hESCs might reflect cytoskeletal damage that interferes with both long-term proliferation of cells and motility. The future work should address the molecular underpinning of superdiffusive behavior and its association to survival and proliferation of hESCs, but this is likely to involve proteins that mediate actin cytoskeleton dynamics and/or proteins that regulate the crosstalk between cell motility and survival. ROCK is an example of one such protein, and certainly, inhibition of ROCK in the normal cells at low density enhanced motility as well as clonogenicity. The prosurvival phosphatidylinositol 3-kinase/Akt pathway has also been linked to motility in other cell types (Xue and Hemmings, 2013). Given that clonogenic/superdiffusive cells are very rare in normal hESC populations, identification of candidate genes will require either enriching the proportion of superdiffusive cells by modifying culture conditions or developing reliable techniques to isolate superdiffusive cells before subjecting them to gene expression analysis to identify the subset of genes that are differentially expressed in this particular fraction compared to Brownian or nonmotile cells.

Similar to previously reported effects of plating cell density on cell motility (Li et al., 2010), we noted that at high-plating density, cells exhibited enhanced motility, thus allowing nonsister cell contacts through migration. Under these high-density conditions, the normal hESCs that gave rise to colonies came not only from superdiffusive cells but also from the Brownian-like cells, thus now mimicking the behavior of adapted cells. Hence, the observed nonlinear rise in the growth of normal cells at increasing plating densities can be explained by an increased chance for cell:cell contact resulting from both a reduced average distance between cells and by increasing the motility of cells.

In contrast to the normal cells, the requirement for cell:cell contacts to promote survival was removed in the H7 and H14 adapted cells, implying the loss of niche dependence, as has also been reported elsewhere (Werbowetski-Ogilvie et al., 2009). Consequently, the functional significance of the observed increased motility of adapted cells remains unclear. One possibility is that increased motility plays a role in the early stages of adaptation, allowing cells to make more contacts, but becomes superfluous once the adapted cells acquire further changes that render them cell contact independent. If this is the case, monitoring motility could provide a noninvasive approach for the early detection of culture adaptation.

Time-lapse analysis reveals the marked complexity of hESC cultures and the multifaceted interplay of the cell interactions that drive the behavior of these cells and, ultimately, their fate choices. Our study has demonstrated not only the importance of signals mediated by cell:cell contact in promoting hESC proliferation but also the heterogeneity of these cells with respect to their clonogenic and motility patterns, which are influenced by diffusible signaling molecules. Furthermore, that all three bottlenecks to survival of hESCs during passaging were overcome by the ROCK inhibitor as well as adaptation in both H7 and H14 hESCs suggests a common pathway to overcoming these bottlenecks involving signaling associated with the cytoskeleton, which could also influence cell motility. Understanding the molecular basis for these different signals and how they interact will be essential for designing culture conditions that minimize the selective advantages of particular mutations and so reduce the occurrence of variant cells during prolonged culture and scale-up.

## Experimental Procedures

### Cell Culture

hESC lines used in this study were H7 and H14 (Thomson et al., 1998). Karyotypically normal sublines of H7 and H14 are termed H7.s14 and H14.s9, respectively. Karyotypically abnormal sublines of H7 (termed H7.s6) (47,XX,+1,der(6)t(6;17) (q27;q1)) and H14 (termed H14.BJ1) (48,XY,+12,+der(17) (p12p13.3) hsr(17) (p11.2)) have arisen spontaneously in culture (Baker et al., 2007; Draper et al., 2004; Enver et al., 2005).

### Time-Lapse Video Microscopy

Time-lapse microscopy was performed by taking one frame every 10 min over 72 hr, using an Olympus Ix70 microscope controlled by Simple PCI software (Compix). During imaging, cells were enclosed in a chamber maintained at 37°C under a humidified atmosphere of 5% CO_2_ in air.

### Motility Analysis

Cell motility was analyzed using fractional diffusion techniques from Dieterich et al. (2008). The Ornstein-Uhlenbeck model was used to characterize Brownian-like motion, and the fractional Klein-Kramers (FKK) was used to characterize superdiffusive motion.

Detailed experimental methods and a description of statistical analysis, lineage tree reconstruction, segmentation, and tracking are provided in Supplemental Experimental Procedures.

## Author Contributions

I.B., V.B., D.C., and P.W.A. conceived and designed the experiments. I.B., V.B., P.J.G., M.J., D.S., and A.G. performed the experiments. V.B. and D.C. developed numerical algorithms. I.B. and V.B. analyzed the data. I.B., V.B., P.J.G., D.C., and P.W.A. wrote the paper.

## Supplemental Information

Document S1. Supplemental Experimental Procedures, Figures S1–S5, and Tables S1 and S2

Movie S1. Time-Lapse Video of Normal H7.s14 Cells Plated at Low Density and Reconstructed Lineage Trees over 72 hr Postplating, Related to Figure 1 Initial plated cells labeled 1–18 correspond to those in Figure 1A (uppermost panel), and lineage trees are also included in Figure 2A.

Movie S2. Time-Lapse Video of Adapted H7.s6 Cells Plated at Low Density and Reconstructed Lineage Trees over 72 hr Postplating, Related to Figure 1 Initial plated cells labeled 1–12 correspond to those in Figure 1B (uppermost panel), and lineage trees are also included in Figure 2B.

Movie S3. Time-Lapse Video of Normal H7.s14 Cells Treated with Y-27632, Plated at Low Density, and Reconstructed Lineage Trees over 72 hr Postplating, Related to Figures 2 and S1 Initial plated cells labeled 1–19 correspond to those in Figure S1A (uppermost panel), and corresponding lineage trees are also included in Figure S1B. Also see Figure 2.

Movie S4. Time-Lapse Video of Adapted H7.s6 Cells Treated with Y-27632, Plated at Low Density, and Reconstructed Lineage Trees over 72 hr Postplating, Related to Figures 2 and S1 Initial plated cells labeled 1–14 correspond to those in Figure S1C (uppermost panel), and corresponding lineage trees are also included in Figure S1D. Also see Figure 2.

Movie S5. Representative Example of a Three-Cell Clump of Normal H7.s14 Cells Dissociated Using Nonenzymatic Solution and Observed in Time-Lapse over 72 hr Postplating, Related to Figure 5 Initial plated cells in the clump correspond to those in Figure 5A (uppermost panel), and lineage trees are included in Figure 5B.

Movie S6. Representative Examples of Cells Tracked from Time-Lapse Showing Superdiffusive, Brownian-like, and Nonmotile Cells from the H7 hESC Sublines Plated at Low Density, Related to Figures 7 and S4

Movie S7. Single-Cell Tracking of Time-Lapse Video Showing Normal H7.s14 Cells Plated at High Density Observed in Time-Lapse over 12 hr after Plating, Related to Figure 7 Cell labels correspond to those in Figure S5B. Also see Figure 7.

Document S2. Article plus Supplemental Information
