# Supplementary material for: Time-Lapse Analysis of Human Embryonic Stem Cells Reveals Multiple Bottlenecks Restricting Colony Formation and Their Relief upon Culture Adaptation
Source: Stem Cell Reports. 2014 Jun 12;3(1):142–55. doi: 10.1016/j.stemcr.2014.05.006 (PMC4110749; doi:10.1016/j.stemcr.2014.05.006)
Supplement: Document S1. Supplemental Experimental Procedures, Figures S1–S5, and Tables S1 and S2 [file mmc1.pdf]

Stem Cell Reports, Volume *3*

Supplemental Information

Time-Lapse Analysis of Human Embryonic Stem Cells Reveals Multiple Bottlenecks Restricting Colony Formation and Their Relief upon Culture Adaptation

Ivana Barbaric, Veronica Biga, Paul J. Gokhale, Mark Jones, Dylan Stavish, Adam Glen, Daniel Coca, and Peter W. Andrews

Figure S1.

A Normal (H7.s14) cells treated with Y-27632, plated at 4,300 cells/cm²

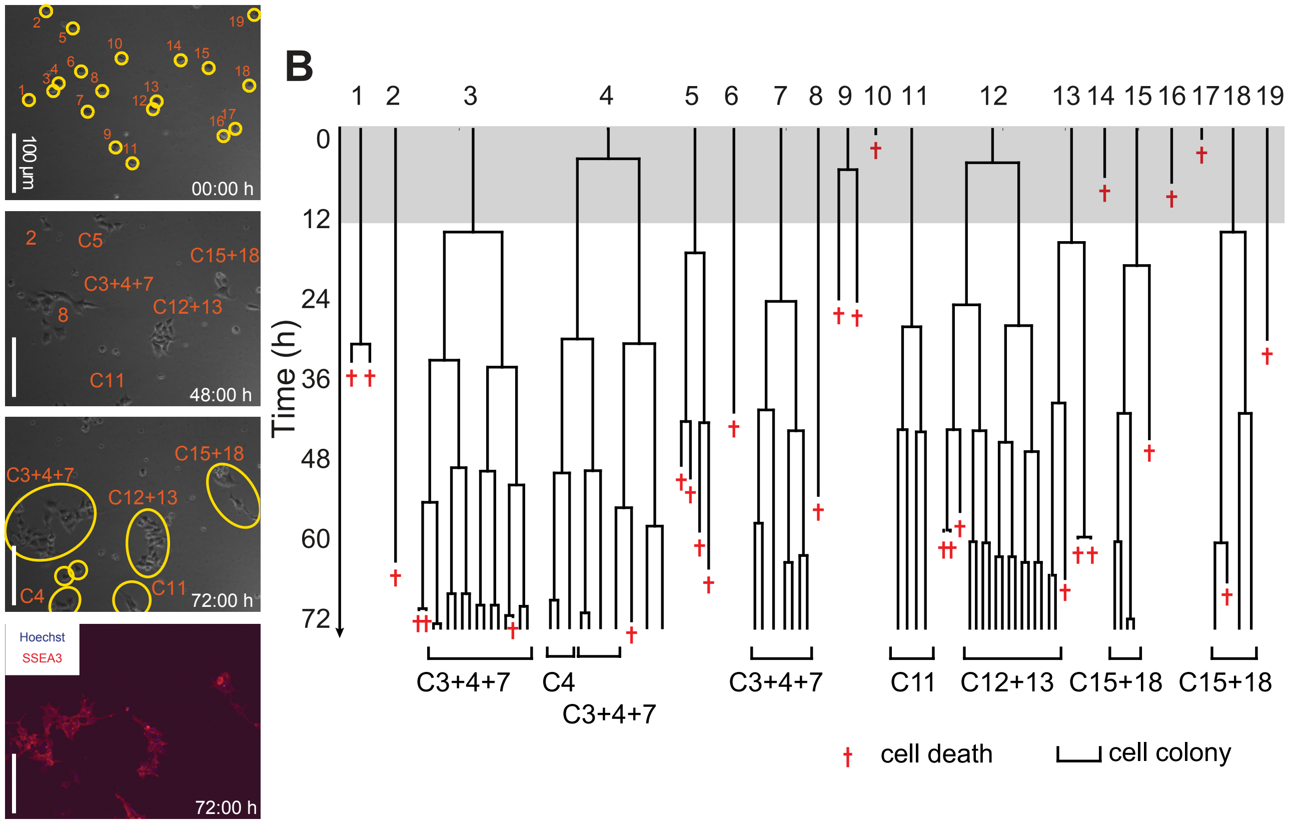

C Adapted (H7.s6) cells treated with Y-27632, plated at 2,100 cells/cm²

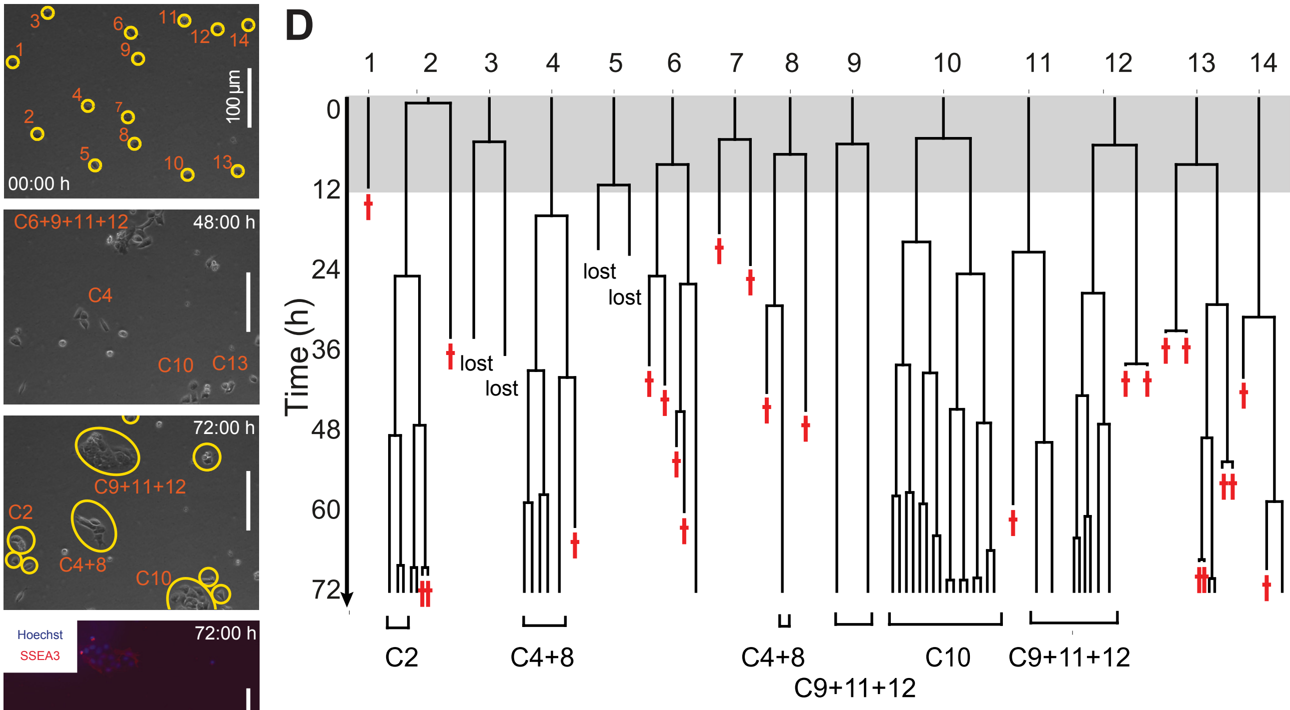

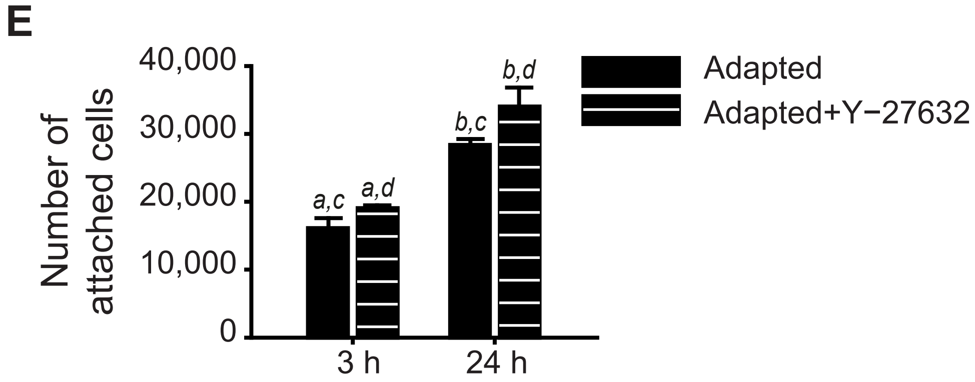

Figure S2.

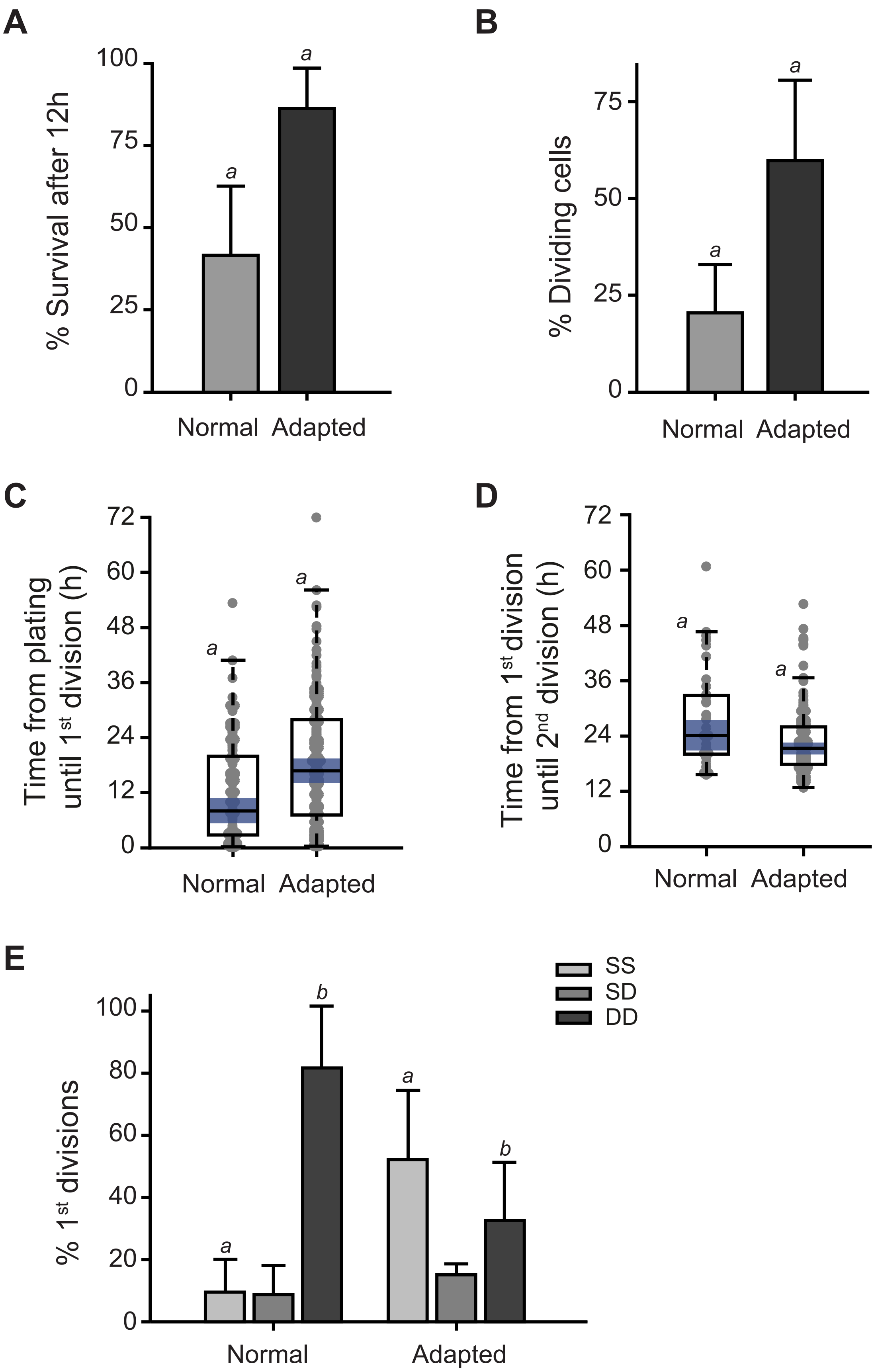
A
% Survival after 12h
100
75
50
25
0
a
a
Normal
Adapted
B
% Dividing cells
75
50
25
0
a
a
Normal
Adapted
C
Time from plating until 1st division (h)
72
60
48
36
24
12
0
a
a
Normal
Adapted
D
Time from 1st division until 2nd division (h)
72
60
48
36
24
12
0
a
a
Normal
Adapted
E
% 1st divisions
100
80
60
40
20
0
SS
SD
DD
a
b
a
b
Normal
Adapted

Figure S3.

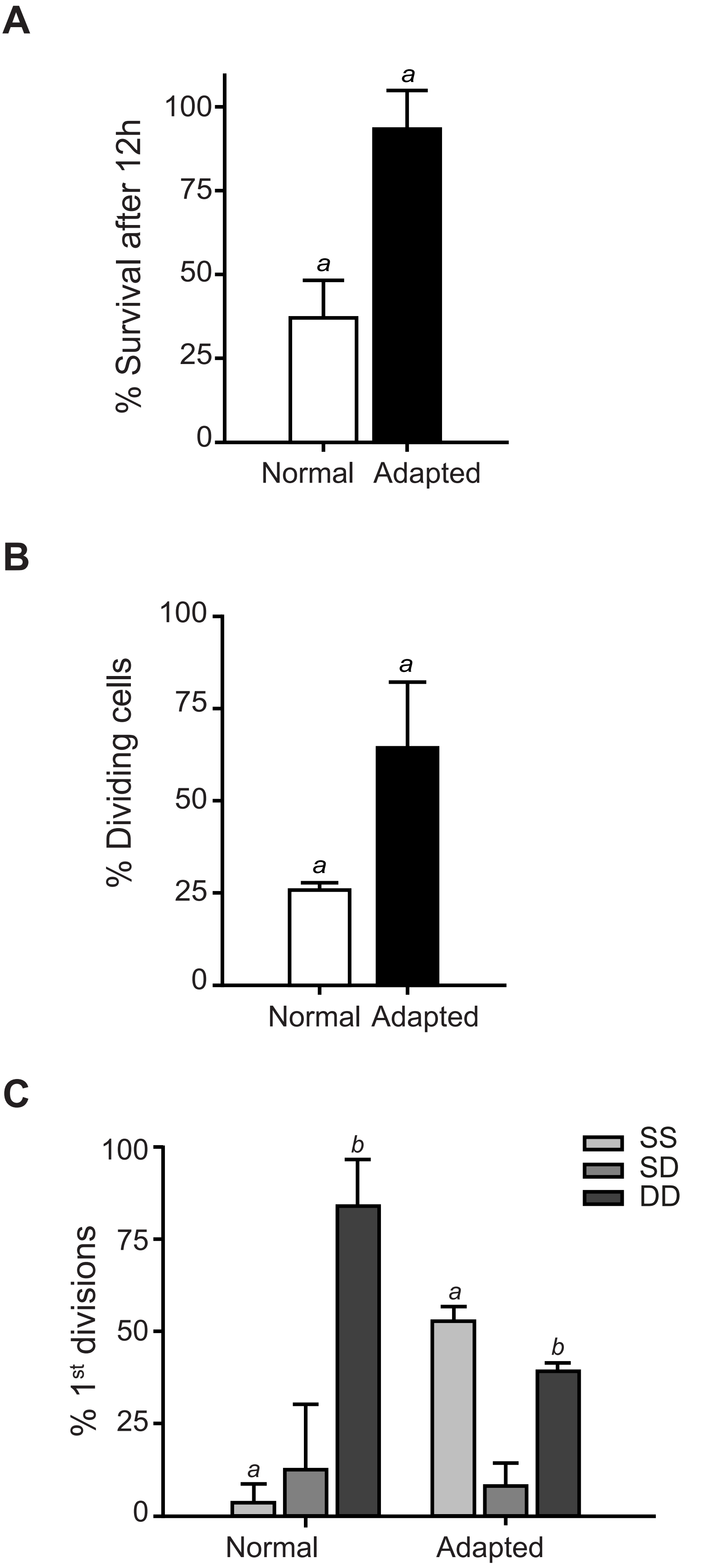

A
% Survival after 12h
100
75
50
25
0
a
a
Normal
Adapted
B
% Dividing cells
100
75
50
25
0
a
a
Normal
Adapted
C
% 1st divisions
100
75
50
25
0
SS
SD
DD
a
b
a
b
Normal
Adapted

Figure S4.

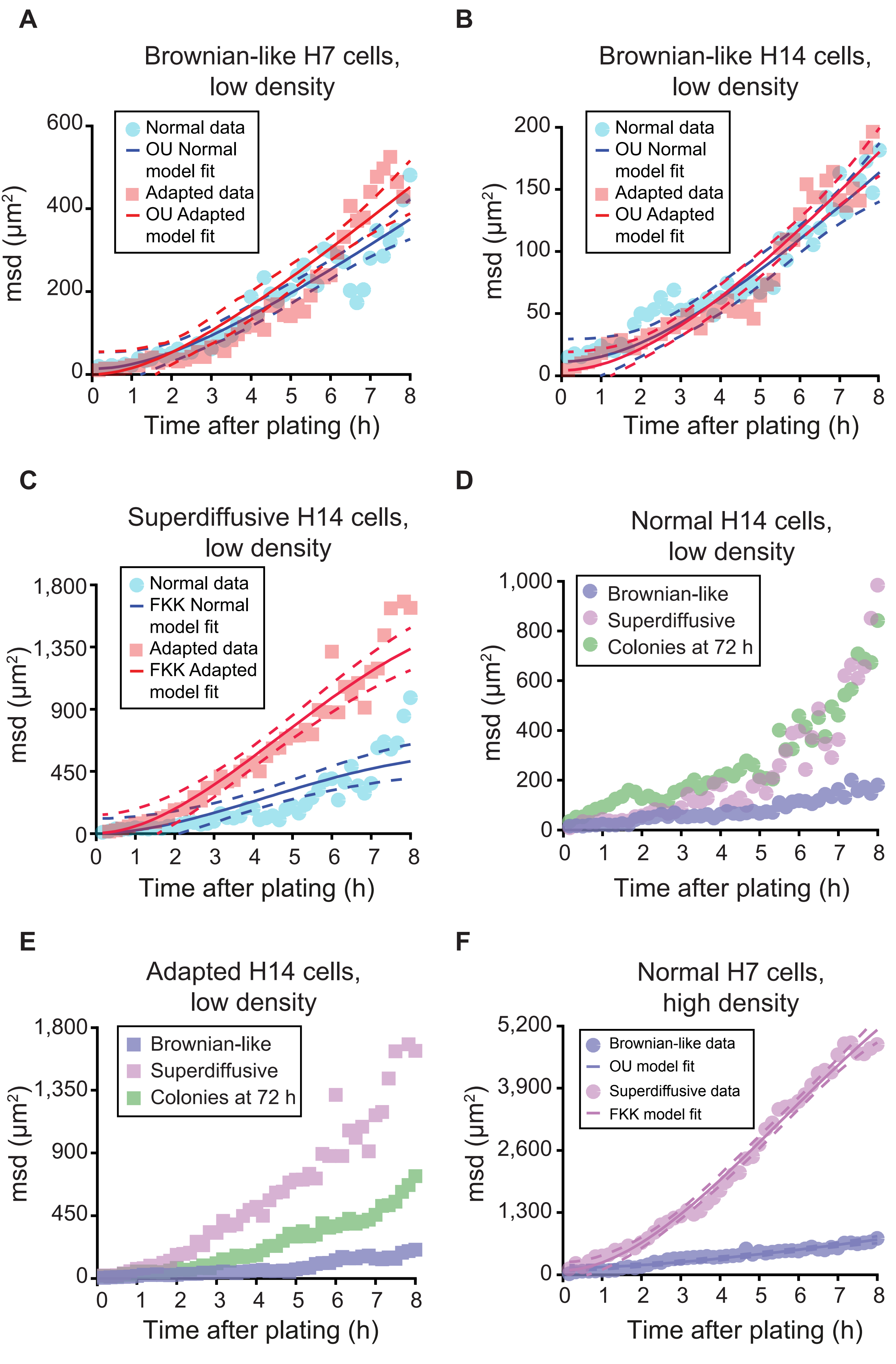
A
Brownian-like H7 cells, low density
Normal data
OU Normal model fit
Adapted data
OU Adapted model fit
msd (μm²)
Time after plating (h)
B
Brownian-like H14 cells, low density
Normal data
OU Normal model fit
Adapted data
OU Adapted model fit
msd (μm²)
Time after plating (h)
C
Superdiffusive H14 cells, low density
Normal data
FKK Normal model fit
Adapted data
FKK Adapted model fit
msd (μm²)
Time after plating (h)
D
Normal H14 cells, low density
Brownian-like
Superdiffusive
Colonies at 72 h
msd (μm²)
Time after plating (h)
E
Adapted H14 cells, low density
Brownian-like
Superdiffusive
Colonies at 72 h
msd (μm²)
Time after plating (h)
F
Normal H7 cells, high density
Brownian-like data
OU model fit
Superdiffusive data
FKK model fit
msd (μm²)
Time after plating (h)

Figure S5.

A

Normal (H7.s14) cells plated at low density (2,100 cells/cm^2)

B

Normal (H7.s14) cells plated at high density (10,600 cells/cm^2)

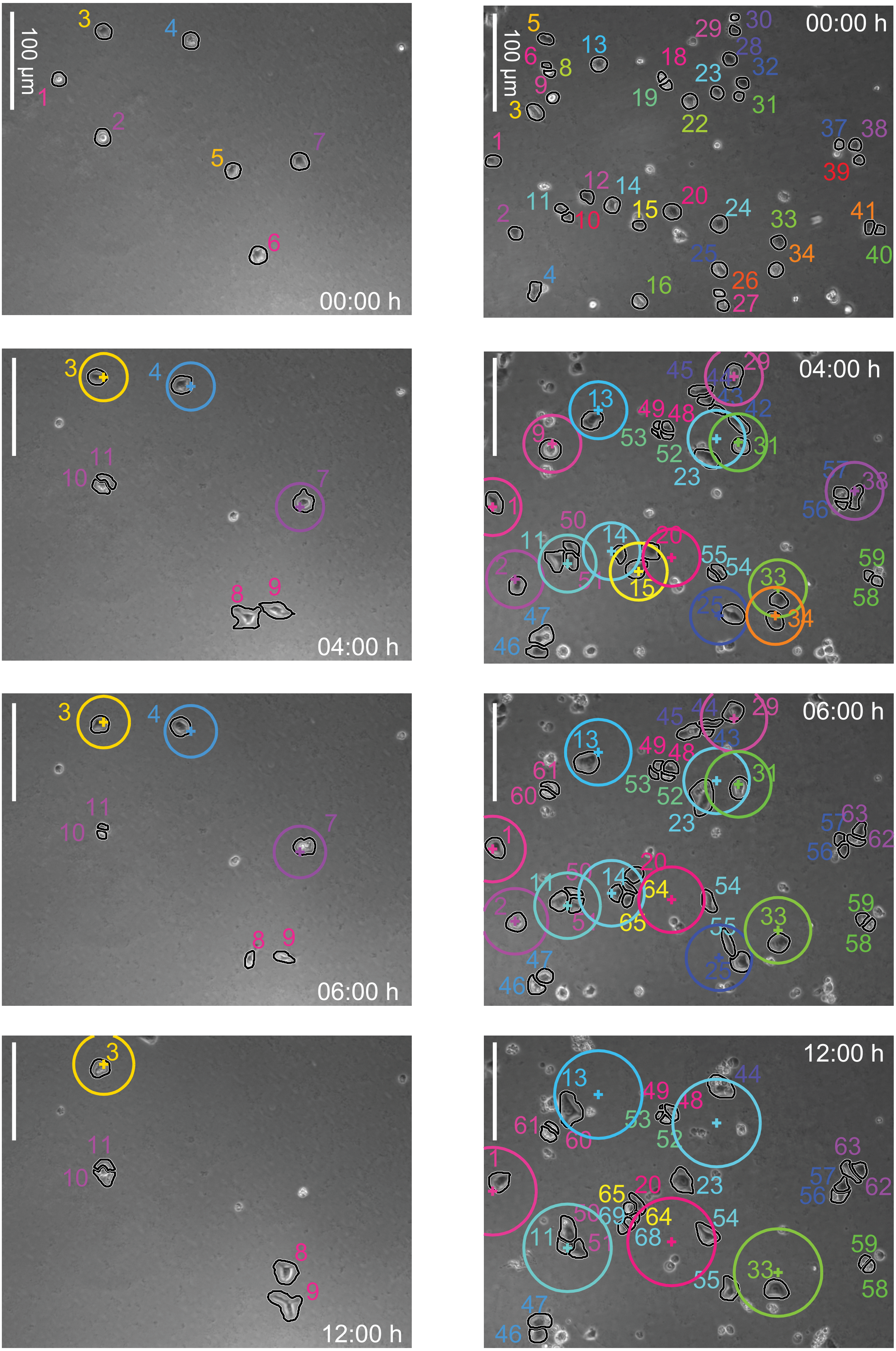

Supplemental Figure Legends

Figure S1. Single-cell behaviour of normal H7.s14 and adapted H7.s6 cells treated with the ROCK inhibitor Y-27632 investigated by time-lapse analysis.

(**A**) Time-lapse analysis of SSEA3-sorted normal cells treated with Y-27632 plated at low density. Still images are taken from **Movie S3** (available online). Cells labelled 1 to 19 are circled in the upper-most panel. Colonies present at 72h were partly from single cells (colonies labelled C4 and C11 originating from cells 4 and 11 respectively) and partly mixed (colonies labelled C3+4+7, C12+13 and C15+18 originating from cells with corresponding labels) and there were rare satellite colonies formed (cell 4 gives rise to progeny in two colonies labelled C4 and C3+4+7, with the addition of two single cells). The resulting colonies were stained for SSEA3 (red) and nuclei counterstained with Hoechst 33342 (blue) at the end of the filming (lower-most panel).

(**B**) Lineage trees of SSEA3-sorted normal cells treated with Y-27632 constructed from the **Movie S3**. Numbers correspond to cells circled in (**A**, upper-most panel). Colonies present at 72h (clonal colonies are labelled C4 and C11 and non-clonal colonies are labelled C3+4+7, C12+13 and C15+18 according to their constituent originating cells) are also circled in (**A**, the second panel from the bottom). Red crosses denote cell death. Gray shaded area indicates first 12h postplating.

(**C**) Time-lapse analysis of SSEA3-sorted adapted cells treated with Y-27632 plated at low density. Still images are taken from **Movie S4**. Cells labelled 1 to 14 are circled in the upper-most panel. Colonies present at 72h were partly from single cells (colonies labelled C2 and C10 originating from cells 2 and 10 respectively) and partly mixed (for example colonies labelled C4+8 and C9+11+12 originating from cells with corresponding

labels) and we noted single cells leaving colonies (progeny of cell 2 and cell 13 leave clonal colonies and surviving progeny of cell 6 leaves non-clonal colony C9+11+12). The resulting colonies were stained for SSEA3 (red) and nuclei counterstained with Hoechst 33342 (blue) at the end of the filming (lower-most panel).

(**D**) Lineage trees of SSEA3-sorted adapted cells treated with Y-27632 constructed from **Movie S4**. Numbers correspond to cells circled in (**C**, upper-most panel). Colonies present at 72h (clonal colonies labelled C2 and C10 and non-clonal colonies labelled C4+8 and C9+11+12 according to their constituent originating cells) are also circled in (**C**, the second panel from the bottom). Incomplete lineage trees are labelled as 'lost' due to cells escaping from the field of view. Red crosses denote cell death. Gray shaded area indicates the first 12h postplating.

(**E**) Representative numbers of attached adapted (H7.s6) cells and adapted cells treated with Y-27632 analysed after 3h and 24h postplating. Results are means of triplicate wells from the same experiment ± SD (a-d represent Student's t tests, left tail; p values: a<0.05; b<0.05; c<0.001; d=0.001).

Figure S2. Bottlenecks limiting clonal expansion confirmed on another pair of normal cells H14.s9 and their karyotypically adapted counterparts H14.BJ1 analysed at low plating density.

(**A**) Percentage of cells from the initial plated population that survived the first 12h after plating for normal (H14.s9) cells and adapted (H14.BJ1) cells. Results are means of triplicate independent experiments ± SD (a represents Student's t test, left tail; p value<0.01). Data consist of 150 to 340 cells per culture.

(**B**) Percentage of dividing cells in the normal and adapted cultures. Results are means of triplicate independent experiments ± SD (a represents Student's t test, left tail; p value<0.05). Data consist of 150 to 340 cells per culture.

(**C**) The cell cycle re-entry time following plating for normal and adapted cells. Results are box plot representations of distributions from triplicate independent experiments with median line and 95% median confidence intervals (blue); observations (gray dots) shown stretching horizontal by frequency (a represents Kruskal-Wallis test, p value$<10^{-5}$). Data consist of 75 to 165 cells per culture.

(**D**) Cell cycle time in subsequent divisions in normal and adapted cells. Results are box plot representations of distributions from triplicate independent experiments with median line and 95% confidence intervals (blue); observations (gray dots) shown stretching horizontal by frequency (a represents Kruskal-Wallis test, p value<0.05). Data consist of 30 to 120 cells per culture.

(**E**) Percentage of first divisions following which both daughter cells divide further (SS: survival-survival), one daughter cell divides while the other dies (SD: survival-death) or both daughter cells die (DD: death-death) in normal and adapted cultures. Results are means of triplicate independent experiments ± SD (a,b represent Student's t tests, a: left tail, p value<0.05, b: right tail, p value<0.05). Data consist of 60 to 65 cells per culture.

Figure S3. Survival of normal cells H7.s14 and their karyotypically adapted counterparts H7.s6 serially passaged on Matrigel then plated at low plating density and analysed using time-lapse.

(**A**) Percentage of cells from the initial plated population that survived the first 12h after plating for normal (H7.s14) cells and adapted (H7.s6) cells serially passaged on Matrigel. Results are means of triplicate wells from the same experiment ± SD (a represents Student's t test, left tail; p value<0.01). Data consist of 50 to 55 cells per culture.

(**B**) Percentage of dividing cells in the normal and adapted cells serially passaged on Matrigel. Results are means of triplicate wells from the same experiment ± SD (a represents Student's t test, left tail; p value=0.01). Data consist of 50 to 55 cells per culture.

(**C**) Percentage of first divisions following which both daughter cells divide further (SS: survival-survival), one daughter cell divides while the other dies (SD: survival-death) or both daughter cells die (DD: death-death) in normal and adapted cells serially passaged on Matrigel. Results are means of duplicate independent experiments ± SD (a, b represent Student's t tests, a: left tail; p value<0.01, b: right tail; p value<0.05). Data consist of 20 to 30 cell divisions per culture.

Figure S4. Motility of normal and adapted H7 and H14 hESC sublines characterised using fractional diffusion models.

(**A**) Mean squared displacement (msd) calculated from trajectories of Brownian-like normal (H7.s14) and adapted (H7.s6) cells plated at low density showing similar movement characteristics. Data collected in both normal and adapted H7 sublines consisted of cells that were classified according to msd into a Brownian-like and a superdiffusive fraction, with the addition of non motile cells. Only motile cells were analysed, with the msd for super-diffusive cells included in **Figure 7A**. Markers represent

msd from real data, solid lines show predictions of the Ornstein-Uhlenbeck (OU) model which corresponds to Brownian-like motion and dashed lines indicate 95% model prediction bounds. Model parameters are specified in **Table S1**. Data consist of 14 to 21 cells per culture.

(**B**) msd calculated from trajectories of Brownian-like normal (H14.s9) and adapted (H14.BJ1) cells plated at low density showing similar movement characteristics. Data collected in both H14 sublines consisted of cells that were classified according to msd into Brownian-like and superdiffusive, with the addition of non motile cells. Markers represent ensemble msd from real data, solid lines show predictions of the Ornstein-Uhlenbeck (OU) model which corresponds to Brownian-like motion and dashed lines indicate 95% model prediction bounds. Model parameters are specified in **Table S2**. Data consist of 16 to 19 cells per culture.

(**C**) msd calculated from trajectories of superdiffusive normal (H14.s9) and adapted (H14.BJ1) cells plated at low density showing that superdiffusive adapted cells can travel further from origin compared to superdiffusive normal hESCs. Markers represent ensemble msd from real data, solid lines show the predictions of fractional Klein-Kramers (FKK) model, which corresponds to superdiffusive motion and dashed lines indicate 95% prediction bounds. Model parameters are specified in **Table S2**. Data consist of 10 cells per culture.

(**D**) msd for Brownian-like (also included in **B**) and superdiffusive (also included in **C**) normal (H14.s9) cells plated at low density with the rare 8 cells that give rise to colonies present at 72h showing similar movement characteristic as the superdiffusive normal hESCs. Markers represent ensemble msd from real data.

(**E**) msd for Brownian-like (also included in **B**) and superdiffusive (also included in **C**) adapted (H14.BJ1) cells plated at low density, with the cells that give rise to colonies present at 72h showing a mixture of Brownian-like and superdiffusive movement characteristics. Markers represent ensemble msd from real data. Data consist of 23 cells forming colonies.

(**F**) msd calculated from trajectories of Brownian-like and superdiffusive normal (H7.s14) hESCs plated at high density. Markers represent ensemble msd from real data, solid lines show predictions of the Ornstein-Uhlenbeck (OU) and the fractional Klein-Kramers (FKK) models which correspond to Brownian-like and superdiffusive motion respectively; dashed lines indicate 95% model prediction bounds. Model parameters are specified in **Table S1**. Data consist of 18 to 25 cells per category.

Figure S5. Early colony formation in normal H7.s14 cells at different cell plating densities observed in time-lapse microscopy.

(A) Tracking of single cells from time-lapse images of normal (H7.s14) hESCs plated at low density observed over 12h postplating showing that in this culture condition colonies are formed from single cells. Tracked cell boundaries are indicated with black contours and time after plating is specified in each image. Cells at the time of plating (upper-most panel) are labelled 1 to 7 and colour-coded to indicate distinct lineages. Cells 1, 5 and 7 die off during the indicated time period. Cells 2 and 6 divide early and give rise to daughter cells 10, 11 and 8, 9 respectively. Cell 4 is lost from the field of view after 12h (lower-most panel). Colour-coded crosses indicate the location of cells at plating time. With the exception of the upper-most panel, visualisations include colour coded circles

denoting model-predicted locations (cells are expected to remain inside the circled area) at the indicated times after plating. The radius of circles increases with time according to the spatial probability distribution function (pdf) of an equal weighted mixture of Brownian-like and superdiffusive cells. The radius is given by: (i) the distance that 95% of cells are expected to travel up to (for example, we estimated that 95% of cells plated at low density could travel up to 43μm after 12h (see **Figure 7E**) and (ii) added average cell radius (15μm). As a result of reduced motility, colonies present after 12h are clonal as indicated by a unique colour of cells in contact.

(**B**) Tracking of single cells from time-lapse images of normal (H7.s14) hESCs plated at high density taken from **Movie S7** showing that in this culture condition, colonies form from single cells and in addition, there are many non-clonal colonies after 12h postplating. Tracked cell boundaries are indicated with black contours and time after plating is specified in each image. Cells at the time of plating (upper-most panel) are labelled 1 to 41 and colour-coded to indicate distinct lineages. A number of cells are in contact from the time of plating (for example cells 10 and 11). Colonies form through a sequence of death, divisions and migration events. For example, cell 10 dies off; cell 12 divides into daughter cell 51 and daughter cell 50 which subsequently join with cell 11 to form a colony. Cells 14, 15 and 20 start off as single cells but migrate towards each other; cell 15 gives rise to daughter cells 64 and 65, while cell 14 gives rise to daughter cells 68 and 69 thus forming another mixed colony. Colour coded crosses indicate the location of cells at plating time. With the exception of the upper-most panel, visualisations include colour coded model-predicted locations (cells are expected to remain inside the circled area) at the indicated times after plating. The radius of circles increases with time

according to the spatial probability distribution function (pdf) of an equal weighted mixture of Brownian-like and superdiffusive cells. The radius is given by: (i) the distance that 95% of cells are expected to travel up to (for example, at high plating density, we estimated that 95% of cells could travel up to 76μm over 12h postplating time – see **Figure 7E**) and (ii) added average cell radius (15μm). As expected, most cells remain within the predicted area (cells 1, 11, 33 and 13 in the lower-most panel) while few migrate beyond this boundary however remaining in the proximity of the circle (cells 20 and 23 in the lower-most panel). The combined effect of reduced cell:cell distance and enhanced motility at the high density plating condition leads to many colonies present after 12h being non-clonal, as indicated by the multiple colours of cells in contact.

Movie S1. Time-lapse video of normal H7.s14 cells plated at low density and reconstructed lineage trees over 72h postplating. Initial plated cells labelled 1 to 18 correspond to those in **Figure 1A** (upper-most panel) and lineage trees are also included in **Figure 2A.**

Movie S2. Time-lapse video of adapted H7.s6 cells plated at low density and reconstructed lineage trees over 72h postplating. Initial plated cells labelled 1 to 12 correspond to those in **Figure 1B** (upper-most panel) and lineage trees are also included in **Figure 2B.**

Movie S3. Time-lapse video of normal H7.s14 cells treated with Y-27632, plated at low density and reconstructed lineage trees over 72h postplating. Initial plated cells

labelled 1 to 19 correspond to those in **Figure S1A** (upper-most panel) and corresponding lineage trees are also included in **Figure S1B**. Also see **Figure 2**.

Movie S4. Time-lapse video of adapted H7.s6 cells treated with Y-27632, plated at low density and reconstructed lineage trees over 72h postplating. Initial plated cells labelled 1 to 14 correspond to those in **Figure S1C** (upper-most panel) and corresponding lineage trees are also included in **Figure S1D**. Also see **Figure 2**.

Movie S5. Representative example of a three-cell clump of normal H7.s14 cells dissociated using non-enzymatic solution and observed in time-lapse over 72h postplating. Initial plated cells in the clump correspond to those in **Figure 5A** (upper-most panel) and lineage trees are included in **Figure 5B**.

Movie S6. Representative examples of cells tracked from time-lapse showing superdiffusive, Brownian-like and non-motile hESCs plated at low density. Related to **Figure 7** and **Figure S4**.

Movie S7. Single-cell tracking of time-lapse video showing normal H7.s14 cells plated at high density over 12h postplating. Cell labels correspond to those in **Figure S5B**. Also see **Figure 7**.

Supplemental Tables

Table S1. Diffusion model parameters estimated from normal H7.s14 and adapted H7.s6 hESCs at low plating density and normal H7.s14 cells plated at high density.

Population		Thermal speed	Exponent	Damping coefficient	Standard deviation of noise	Diffusion coefficient
Parameter Name		v_{th} $(\mu m/min)$	$\alpha = 2 - \beta$	γ $(1/min^{\alpha})$	η (μm)	D_{α} $(\mu m^2/min^{2-\alpha})$
Normal (H7.s14) Low density	Brownian-like	0.0421 $\pm$ 0.0262	1	0.0066 $\pm$0.0041	2.5866 $\pm$ 2.6632	0.2703 $\pm$ 0.0857
	Super-diffusive	0.0670 $\pm$ 0.0396	0.5506 $\pm$ 0.1014	0.0163 $\pm$ 0.0035	0.3244 $\pm$ 4.1178	0.2749 $\pm$ 0.0653
Adapted (H7.s6) Low density	Brownian-like	0.0493 $\pm$ 0.0323	1	0.0076 $\pm$ 0.0048	0.0001 $\pm$ 3.1066	0.3219 $\pm$ 0.1069
	Super-diffusive	0.0728 $\pm$ 0.0361	0.7000 $\pm$ 0.1161	0.0100 $\pm$ 0.0030	0.0001 $\pm$ 4.0661	0.5302 $\pm$ 0.1134
Normal (H7.s14) High density	Brownian-like	0.0493 $\pm$ 0.0279	1	0.0048 $\pm$ 0.0030	8.4462 $\pm$ 3.1387	0.5025 $\pm$ 0.1484
	Super-diffusive	0.1423 $\pm$ 0.0495	0.6004 $\pm$ 0.0413	0.0149 $\pm$ 0.0013	6.8083 $\pm$ 5.1945	1.3594 $\pm$ 0.1318

Table S2. Diffusion model parameters estimated from normal H14.s9 and adapted H14.BJ1 hESCs plated at low plating density.

Population \ Parameter Name		Thermal speed	Exponent	Damping coefficient	Standard deviation of noise	Diffusion coefficient
		v_{th} $(\mu m/min)$	$\alpha = 2 - \beta$	γ $(1/min^{\alpha})$	η (μm)	D_{α} $(\mu m^2/min^{2-\alpha})$
Normal (H14.s9) Low density	Brownian-like	0.0253 ± 0.0161	1	0.0050 ± 0.0038	2.3734 ± 1.7974	0.1278 ± 0.0433
	Super-diffusive	0.0580 ± 0.0206	0.5319 ± 0.0045	0.0193 ± 0.0002	0.5384 ± 4.1474	0.1749 ± 0.0121
Adapted (H14.BJ1) Low density	Brownian-like	0.0272 ± 0.0146	1	0.0050 ± 0.0027	1.4599 ± 1.6232	0.1475 ± 0.0405
	Super-diffusive	0.0837 ± 0.0207	0.5643 ± 0.0027	0.0175 ± 0.0002	1.0964 ± 4.6012	0.4008 ± 0.0145

Supplemental Experimental Procedures

Human embryonic stem cell culture

Cells were maintained as previously described (Draper et al., 2002) at 37°C under a humidified atmosphere of 5% CO_2 in air, on mitotically-inactivated mouse embryonic fibroblasts (MEFs), in medium consisting of KnockOut DMEM (KO-DMEM) (Life Technologies Ltd, Paisley, UK) supplemented with 20% KnockOut Serum Replacement (Life Technologies), 1mM L-glutamine (Life Technologies), 0.1mM β-mercaptoethanol (Sigma-Aldrich, Poole, Dorset, UK), 1x non-essential amino acids (Life Technologies) and 4ng/ml basic FGF (Life Technologies) (Amit et al., 2000).

Compounds

Y-27632 was purchased from Sigma-Aldrich and used at 10μM in all the assays.

Antibodies

The following primary monoclonal antibodies were prepared in house as pre-titred supernatants from hybridoma MC631 (anti-SSEA3) (Shevinsky et al., 1982); antibody from the parental myeloma P3X63Ag8 (Kohler and Milstein, 1975) was used as a negative control. Secondary antibody used for SSEA3 in immunocytochemistry was Dylight-594-conjugated goat anti-rat IgM, μ -chain specific antibody (Jackson ImmunoResearch, Stratech, Newmarket, UK), and the secondary antibody used in cell sorting was fluorescein isothiocyanate (FITC)-conjugated goat anti-mouse IgG (H+L) antibody (Jackson ImmunoResearch).

Immunocytochemistry

Cells were fixed with 4% paraformaldehyde for 15 min at room temperature and blocked with 10% foetal calf serum in PBS (blocking buffer). They were then incubated with the anti-SSEA3 primary antibody in blocking buffer for 1h. After three washes with PBS, the cells were incubated with the Dylight-594-conjugated secondary antibody in the blocking buffer for 1h. This was followed by three washes with PBS. Nuclei were counter-stained with 10μg/ml Hoechst 33342 (Life Technologies).

Cell sorting

Cells were harvested using trypsin (0.25% trypsin with 0.2% EDTA in PBS) (Sigma-Aldrich) and resuspended in blocking buffer (PBS supplemented with 10% foetal calf serum). Cells were incubated for 30 min with a primary antibody to SSEA3. After washing three times in blocking buffer, cells were labelled with FITC-conjugated secondary antibody for 30 min. This was followed by washing the cells three times with blocking buffer and analyzing cell fluorescence in a MoFlo Cell Sorter (Dako). The gate for FITC-positive cells was set using control cells that were incubated with a negative control antibody obtained from the parent myeloma cell line P3X63Ag8 (Kohler and Milstein, 1975).

For cell sorting based on the cell cycle profile, after harvesting the cells with trypsin and staining for SSEA3 (as described above), cells were washed once in medium and then incubated with 10μg/ml Hoechst33342 (Life Technologies) in medium at 37°C for 30 min. Cells were then sorted using the MoFlo Cell Sorter (Dako).

Cloning efficiency

Cells were sorted for SSEA3 and cell cycle profile and 10,000 normal (H7.s14) or 5,000 adapted (H7.s6) cells were plated onto 6-well plates (Corning Costar, High Wycombe, UK) coated with Matrigel (BD Biosciences, Oxford, UK) (1:25 dilution in KO-DMEM) in mTESR medium (STEMCELL Technologies, Vancouver, Canada). Seven days after seeding the cells, colonies were fixed and stained for SSSEA3 as described above. Images of stained colonies were acquired using an automated microscopy platform (InCell Analyzer 1000, GE Healthcare, Little Chalfont, UK). Numbers of colonies and SSEA3-positive cells were counted from images using the Developer Toolbox 1.7 software (GE Healthcare), as previously described (Barbaric et al., 2010).

Time-lapse video microscopy

For single-cell analysis under the time-lapse, cells sorted for SSEA3 (and in some experiments cells sorted for SSEA3 and cell cycle profile) were plated onto 6-well plates (Corning Costar) coated in Matrigel (BD Biosciences) (1:25 dilution in KO-DMEM), in mTESR medium (STEMCELL Technologies). For analysis of cell behaviour at low plating density, cells were seeded at 2,100 or 4,300 cells/cm^2, whereas the high plating density used was 10,600 cells/cm^2. One frame was taken every 10 min over 72h using an Olympus Ix70 microscope controlled by Simple PCI software (Compix, Brandywine, PA). During imaging, cells were enclosed in a chamber maintained at 37°C under a humidified atmosphere of 5% CO_2 in air. At the end of the time-lapse, cells were fixed and stained for SSEA3. Images of stained cells were acquired using an automated microscopy platform (InCell Analyzer 1000, GE Healthcare). Images were analyzed

using Developer Toolbox 1.9 software (GE Healthcare) as previously described (Barbaric et al., 2010).

For the time-lapse microscopy of single cells after serial passaging on Matrigel, cells grown on MEFs were passaged onto Matrigel in mTESR and maintained in these conditions for three to four passages. SSEA3-sorted single cells were then plated onto 6-well plates coated in Matrigel in mTESR medium and filmed as described above.

For the time-lapse microscopy of cells passaged in small clumps, cells grown in colonies on MEFs were passaged using Cell Dissociation Solution (Biological Industries, Kibbutz Beit-Haemek, Israel) to obtain clumps of approximately three to four cells. Clumps were then plated onto 6-well plates coated in Matrigel in mTESR medium and filmed as described above.

Statistical analysis

Statistical analysis was produced using standard methods from the Statistics Toolbox of Matlab R2010b. Replicates were created from independent experiments or wells from the same experiment as indicated in figure legends. Bar plot analysis consist of population counts from multiple experiments as a ratio of the entire population and indicates mean values with whiskers denoting one standard deviation (SD) above the mean. Bar plot results were Gaussian-distributed and compared using the 2-sample Student's t test using left tail or right tail to ascertain whether one mean is lower or higher than the other respectively.

Box plot analysis indicates values between the 25th (q_1) and 75th (q_2) percentile as the bottom and top edges of the box; maximum whiskers length is w = 2.7SD; values lying

outside the whisker length on either side of the box (lower than $q_2 + w (q_2 – q_1)$ or higher than $q_1 – w (q_2 – q_1)$, respectively) are considered outliers; the level of the distribution is indicated by the median value and 95% median confidence intervals. Box plots are accompanied by a display of data using gray dots binned by value indicated on the y-axis and stretching horizontally by frequency. Distributions in the boxplot analysis were non-Gaussian and comparisons were made using the Kruskal-Wallis test. Tests were performed pairwise and we considered significant those differences with an associated p value < 0.05.

Lineage tree reconstruction

Lineage datasets were collected partly using an automated tracker and partly manually. Data was recorded in an electronic format, which allowed performing statistical analyses and visualisations. The lineage tree data structure is unique to each time-lapse field and contains information of the time of death or division and progeny (where applicable) for all recorded cells, the number of cells at plating time and the length of the time-lapse experiment. Cells are denoted in the data by a unique numeric label 1,2,.. and in cases where a division event occurs, unique numeric labels are created for the two daughter cells, such that the total number of cells recorded is continuously increasing. All numeric labels are stored into an organised data structure linking the parent cell to its progeny. Where the automated extractor output was used, it has been compared to the time-lapse video data to ensure that death and division events were correctly detected.

Segmentation and tracking of cell trajectories

We developed an automated segmentation and tracking model, which allows the detection of multiple non-overlapping objects with coupled geometric active contours. A detailed review of geometric active contour models may be found in (Cremers et al., 2007). Our algorithm relies on the partitioning of an image into two regions (target and background), with the target region further partitioned into multiple connected regions representing cell interiors (Biga et al., manuscript in preparation). Tracking was achieved by feeding the processing result of the current frame to be used as initialisation in processing the following one. Information regarding cell fate (death or division), death or division time, progeny (where applicable), as well as time series of cell location (representing un-weighted centre of mass coordinates of the region corresponding to each cell over time) and other cell statistics (morphology, relative cell:cell distance) was extracted in an automated fashion and stored in a data structure ready to be used in statistical analysis and visualisations.

Cell:cell contact detection and quantification from time-lapse images

We considered a colony to be a collection of a minimum of two cells, where any cell in the collection is found in direct cell:cell contact with at least one other cell in the same group. In automated data extraction, cell:cell contacts were identified using the geometric active contours representation of cell interior and cell boundary. The contours of any particular cell evolved from the initial cell representation at plating time $t = 0$ to subsequent representations at postplating times $t > 0$. The distance between any two

segmented cells was computed as the minimum Euclidean distance between any two pixels from contour pairs:

$$d_{ij}(t) = \min\left\{\sqrt{\left(x_i(t) - x_j(t)\right)^2 + \left(y_i(t) - y_j(t)\right)^2}, \forall j = \overline{1{:}N(t)}, j \neq i\right\} \quad (1)$$

where $(x_i(t), y_i(t))$ denote boundary pixels and $N(t)$ represents the total number of cells alive at time t. Any two cells are considered in direct cell:cell contact at time t if their contours satisfy the condition $d_{ij}(t) = 0$.

The proportion of cells that had come into contact with one or more other cells was calculated from multiple fields collected from independent experiments. It represents a cumulative count of all cells that had at least one contact during the observation period indicated on the x-axis, proportional to the total number of initial plated cells present in all analysed fields. Where more than one contact is indicated, only the ratio (number of cells that had n contacts proportional to the total number of cells) for the highest number of contacts (denoted by n) is considered. For example, if cell A had a single neighbour up to 2h postplating but then acquired two neighbours at 3h, then A is included in the bin count for $n = 1$ from 0h to <3h, however after 3h postplating A is included in the bin count $n = 2$.

Movement classification for single cell trajectories

Trajectories of single cells were extracted from video data as timeseries of coordinates $(x_i(t), y_i(t))$, denoting the center of mass (corresponding to active contours identifying each cell interior) in consecutive frames observed up to 12h postplating. We computed the time-averaged $msd_i, i = \overline{1{:}N(0)}$ (Kusumi et al., 1993),(Qian et al., 1991):

$$\mathrm{msd_i(k\delta t)} = \frac{1}{\mathrm{K-k-1}}\sum_{\mathrm{j=1}}^{\mathrm{K-k-1}}\left[\left(\mathrm{x_i(j\delta t + k\delta t) - x_i(j\delta t)}\right)^2 + \left(\mathrm{y_i(j\delta t + k\delta t) - y_i(j\delta t)}\right)^2\right] \quad (2)$$

Where K is the lifespan of cell i and k represents the number of time lags δt.

The average cell radius was estimated to be the same for normal and adapted cells in a particular cell line: approximately 15μm in H7 and 11μm in H14, which is consistent with reports from literature (Li et al., 2010).

The $\mathrm{msd_i}$ of cells that showed superdiffusive movement transitioned from a stretched-exponential to a power law behaviour of type $\mathrm{msd} \sim \mathrm{t}^{\beta}$ in the long time limit, $\mathrm{t} \rightarrow \infty$ (Metzler and Klafter, 2000). Therefore we considered only the motile cells that had long timeseries of cell locations to show a clear transition (> 5h).

We adapted the method in (Kusumi et al., 1993) to take into account long time characteristics of movement. We characterised the $\mathrm{msd_i}$ shape through a linear fitting across the entire length of the curve. We then assessed the performance of the linear fitting to be sufficient if it explained a large proportion of the variance in the data ($\mathrm{rsquare} \geq 0.95$) and the root mean squared error (rmse) was small ($\mathrm{rmse} \leq 0.05$). These cells were referred to as Brownian-like. For the remaining curves, we varied the endpoint of the linear part to find the best fitting line satisfying $\mathrm{rsquare} \geq 0.95$ and $\mathrm{rmse} \leq 0.05$ and considered a measure of relative deviation from linearity:

$$\mathrm{RD_i} = \frac{1}{\mathrm{K}}\sum_{\mathrm{k=1}}^{\mathrm{K}} \frac{\mathrm{msd_i(k\delta t)}}{4\hat{\mathrm{a}}\mathrm{k}\delta\mathrm{t} + \hat{\mathrm{b}}} \quad (3)$$

where $\hat{\mathrm{a}}, \hat{\mathrm{b}}$ are the estimated linear coefficients. Cells with corresponding values of $\mathrm{RD_i} > 1$ were considered superdiffusive, while $\mathrm{RD_i} < 1$ were considered subdiffusive.

Although the time-averaged mean squared displacement (2) has become widely used (Ruthardt et al., 2011), it is known that uncertainty in the curve increases with

increasing time-lag $k\delta t$ due to few remaining displacements. Thus, the recommended length to be analysed reliably is approximately $1/4$ of the total number of data points available (Saxton and Jacobson, 1997). However, in the case of our data the majority of normal cells died or divided early after plating leading to extremely few cells from the initial plated population to be observed after 12h postplating. We retained $2/5$ of the length of points available, representing approximately 5h at an acquisition rate of 1 frame every 10min. We classified the type of diffusion from the time-averaged msd and further analysed the cell movement characteristics from the ensemble average msd, which is a population measure and does not suffer from these statistical problems.

Motility analysis using fractional diffusion models

Assuming at time t postplating, a number of $N(t)$ surviving (but not divided) cells are found with corresponding coordinates $(x_i(t), y_i(t))$, the ensemble average mean squared displacement was computed as:

$$\mathrm{msd}(t) = \frac{1}{N(t)} \sum_{i=1}^{N(t)} \left[\left(x_i(t) - x_i(0)\right)^2 + \left(y_i(t) - y_i(0)\right)^2 \right]. \qquad (4)$$

Given the two subpopulations of cells identified as Brownian-like and superdiffusive, we computed the msd (4) for each of these separately. To exclude remaining heterogeneity in the movement of each subpopulation, cells that had a contribution of over five times of that of remaining cells were excluded from the motility analysis. We analysed the msd behaviour up to 8h postplating, which coincides with division time of normal cells.

We fitted the msd data using a fractional diffusion model, the Klein-Kramers equation, which describes superdiffusive processes ($1 < \beta \leq 2$) and for $\beta = 1$ reduces to an Ornstein-Uhlenbeck model (Barkai and Silbey, 2000),(Metzler and Klafter, 2000).

Recent work in (Dieterich et al., 2008) has shown that the fractional Klein-Kramers equation can model not only the transition from stretched exponential to power law behaviour $msd \sim t^{\beta}$, for any $1 < \beta \leq 2$ but also characterize complex statistics such as the spread of cells over time as the spatial probability distribution function (pdf) more accurately than the standard Ornstein-Uhlenbeck model which assumes $\beta = 1$. A major advantage of both these models is that they allow characterizing the dynamics of cells exhibiting different types of movement, in different culture conditions with a reduced set of parameters.

We followed the approach proposed in (Dieterich et al., 2008) to model the mean squared displacement curves. The Ornstein-Uhlenbeck equation was used to model the msd for Brownian-like normal and adapted cells:

$$msd_{OU}(t) = 4\frac{{v_{th}}^2}{\gamma^2}(\gamma t - 1 + \exp(-\gamma t)) + (2\eta)^2 \quad (5)$$

where v_{th} , γ, η denote thermal speed, damping and standard deviation of noise respectively. The msd for superdiffusive normal and adapted cells was modelled with the fractional Klein-Kramers equation:

$$msd_{FKK}(t) = 4{v_{th}}^2 t^2 E_{\alpha,3}(-\gamma t^{\alpha}) + (2\eta)^2 \quad (6)$$

where:

$$E_{\alpha,3}(z) = \sum_{j=0}^{\infty} \frac{z^j}{\Gamma(\alpha + 3j)} \quad (7)$$

denotes the generalised Mittag-Leffler function and the exponent parameter $\alpha = 2 - \beta$. Using the Fox H function (Metzler and Klafter, 2000), (7) can be expressed as:

$$E_{\alpha,\delta}(z) = H_{1,2}^{1,1}\left[-z\middle|\begin{matrix}(0,1)\\(0,1),(1-\alpha,\delta)\end{matrix}\right] \tag{8}$$

In order to compute the Fox H function numerically, we first deduced that for $\delta = 3$ (8) can be expressed as a Meijer G function (Mathai and Hauboldt, 2008):

$$E_{\alpha,3}(z) = \frac{2\pi}{3^{\alpha-0.5}} G_{1,4}^{1,1}\left[-\frac{z}{27}\middle|\begin{matrix}0\\0, 1-\frac{\alpha}{3}, 1-\frac{\alpha+1}{3}, 1-\frac{\alpha+2}{3}\end{matrix}\right] \tag{9}$$

The Meijer G Function was computed in Matlab using the symbolic toolbox MuPAD.

For the Brownian-like normal and adapted cells, which are characterised by the Ornstein-Uhlenbeck model, the spatial probability distribution function (pdf) of a cell to travel the distance δx after time t was generated with the Gaussian distribution:

$$p_{OU}(\delta x, t) = \frac{1}{\sqrt{4\pi D_1 t}} \exp\left(\frac{-\delta x^2}{4D_1 t}\right) \tag{10}$$

where $D_1 = {vth^2}/{\gamma}$ denotes the generalised diffusion coefficient for $\alpha = 1$.

For the superdiffusive normal and adapted cells, the spatial pdf corresponding to the fractional Klein-Kramers model was generated as (Metzler and Klafter, 2000):

$$p_{FKK}(\delta x, t) = \frac{1}{\sqrt{4\pi D_\alpha t^{2-\alpha}}} H_{1,2}^{2,0}\left[\frac{\delta x^2}{4D_\alpha t^{2-\alpha}}\middle|\begin{matrix}(\alpha/2, 2-\alpha)\\(0,1),(0.5,1)\end{matrix}\right] \tag{11}$$

We deduced the transformation from Fox H function to Meijer G function by assuming $\beta = 2 - \alpha$ is a rational number $\beta = {k}/{l}, k, l \in \mathbb{Z}^*$ (Mathai and Hauboldt, 2008):

$$H_{1,2}^{2,0}\left[z\middle|\begin{matrix}(\alpha/2, 2-\alpha)\\(0,1),(0.5,1)\end{matrix}\right] =$$

$$= \frac{(2\pi)^{\frac{k-2l+1}{2}} l^{\frac{1}{2}}}{k^{\frac{l-k}{2l}}} G_{k,2l}^{2l,0}\left[\frac{z^l k^k}{l^l}\middle|\begin{matrix}-\frac{1}{2l}+\frac{1}{k}, -\frac{1}{2l}+\frac{2}{k}, \dots, -\frac{1}{2l}+1\\\left(0,\frac{1}{l},\dots,\frac{l-1}{l}\right),\left(\frac{1}{2l},\frac{1}{2l}+\frac{1}{l},\dots,\frac{1}{2l}+\frac{l-1}{l}\right)\end{matrix}\right] \tag{12}$$

The overall pdf for the motile normal and adapted cell cultures over time is computed as an equally weighted mixture with known mixing components $n_1, n_2 = 0.5$:

$$p(\delta x, t) = n_1 p_{OU}(\delta x, t) + n_2 p_{FKK}(\delta x, t) \tag{13}$$

Where p_{OU}, p_{FKK} are the pdfs for Brownian-like cells (parameters estimated with the Ornstein-Uhlenbeck model) and superdiffusive cells (parameters estimated with the fractional Klein-Kramers models) respectively.

Model parameters were estimated using the trust region reflective algorithm for nonlinear least squares fitting with boundary constraint > 0 for all parameters and in addition $\alpha \in (0,2)$. Nonlinear regression functions from the Statistics Toolbox were used to compute standard deviations for the parameters and 95% model prediction bounds. The model parameter estimates are included in **Table S1** and **Table S2**.

To calculate the distance that 95% of cells can travel up to with respect to the point of origin we considered the cumulative distribution function (cdf), associated to the pdf, which measures the probability that cells may be observed at distances $\leq$ x. Thus, the 95% maximum distance satisfies $\mathrm{cdf}(x, t) = 0.95$.

All data extraction, visualisations and algorithms were implemented in Matlab R2010b. For further information please contact the corresponding author at d.coca@sheffield.ac.uk.

Supplemental References

Amit, M., Carpenter, M.K., Inokuma, M.S., Chiu, C.P., Harris, C.P., Waknitz, M.A., Itskovitz-Eldor, J., and Thomson, J.A. (2000). Clonally derived human embryonic stem cell lines maintain pluripotency and proliferative potential for prolonged periods of culture. Dev Biol 227, 271-278.
Barkai, E., and Silbey, R.J. (2000). Fractional Kramers Equation. The Journal of Physical Chemistry B 104, 3866-3874.
Cremers, D., Rousson, M., and Deriche, R. (2007). A Review of Statistical Approaches to Level Set Segmentation: Integrating Color, Texture, Motion and Shape. Int J Comput Vision 72, 195-215.
Kohler, G., and Milstein, C. (1975). Continuous cultures of fused cells secreting antibody of predefined specificity. Nature 256, 495-497.
Kusumi, A., Sako, Y., and Yamamoto, M. (1993). Confined lateral diffusion of membrane receptors as studied by single particle tracking (nanovid microscopy). Effects of calcium-induced differentiation in cultured epithelial cells. Biophys J 65, 2021-2040.
Mathai, A.M., Hauboldt, H.J. (2008). Special functions for applied scientists (New York, NY: Springer Science and Business Media).
Qian, H., Sheetz, M.P., and Elson, E.L. (1991). Single particle tracking. Analysis of diffusion and flow in two-dimensional systems. Biophys J 60, 910-921.
Ruthardt, N., Lamb, D.C., and Brauchle, C. (2011). Single-particle Tracking as a Quantitative Microscopy-based Approach to Unravel Cell Entry Mechanisms of Viruses and Pharmaceutical Nanoparticles. Mol Ther 19, 1199-1211.
Saxton, M.J., and Jacobson, K. (1997). Single-particle tracking: applications to membrane dynamics. Annu Rev Biophys Biomolec Struct 26, 373-399.
Shevinsky, L.H., Knowles, B.B., Damjanov, I., and Solter, D. (1982). Monoclonal antobody to murine embryos defines a stage-specific embryonic antigen expressed on mouse embryos and human teratocarcinoma cell. Cell 30, 697-705.
