# Supplementary material for: Time-Lapse Analysis of Human Embryonic Stem Cells Reveals Multiple Bottlenecks Restricting Colony Formation and Their Relief upon Culture Adaptation
Source: Stem Cell Reports. 2014 Jun 12;3(1):142–55. doi: 10.1016/j.stemcr.2014.05.006 (PMC4110749; doi:10.1016/j.stemcr.2014.05.006)
Supplement: Document S2. Article plus Supplemental Information [file mmc9.pdf]

Stem Cell Reports
Article

OPEN ACCESS

Time-Lapse Analysis of Human Embryonic Stem Cells Reveals Multiple Bottlenecks Restricting Colony Formation and Their Relief upon Culture Adaptation

Ivana Barbaric,[1,3,4] Veronica Biga,[2,4] Paul J. Gokhale,[1] Mark Jones,[1] Dylan Stavish,[1] Adam Glen,[1,3] Daniel Coca,[2,*] and Peter W. Andrews[1,*]
[1]Centre for Stem Cell Biology, Department of Biomedical Science
[2]Centre for Signal Processing and Complex Systems, Department of Automatic Control and Systems Engineering
[3]Department of Materials Science and Engineering
The University of Sheffield, Western Bank, Sheffield S10 2TN, UK
[4]Co-first author
*Correspondence: d.coca@sheffield.ac.uk (D.C.), p.w.andrews@sheffield.ac.uk (P.W.A.)
http://dx.doi.org/10.1016/j.stemcr.2014.05.006
This is an open access article under the CC BY license (http://creativecommons.org/licenses/by/3.0/).

SUMMARY

Using time-lapse imaging, we have identified a series of bottlenecks that restrict growth of early-passage human embryonic stem cells (hESCs) and that are relieved by karyotypically abnormal variants that are selected by prolonged culture. Only a minority of karyotypically normal cells divided after plating, and these were mainly cells in the later stages of cell cycle at the time of plating. Furthermore, the daughter cells showed a continued pattern of cell death after division, so that few formed long-term proliferating colonies. These colony-forming cells showed distinct patterns of cell movement. Increasing cell density enhanced cell movement facilitating cell:cell contact, which resulted in increased proportion of dividing cells and improved survival postplating of normal hESCs. In contrast, most of the karyotypically abnormal cells reentered the cell cycle on plating and gave rise to healthy progeny, without the need for cell:cell contacts and independent of their motility patterns.

INTRODUCTION

Seemingly at odds with their indefinite self-renewing capability in vitro, human embryonic stem cells (hESCs) display a high death rate in culture, contributing to the problems of efficient mass culture. Indeed, although the cell-cycle time of hESCs is relatively short (less than 24 hr) (Becker et al., 2006), hESCs are commonly passaged only every 4–5 days at low split ratios (1:3 or even 1:2), implying a loss of up to 90% of cells from cultures (Olariu et al., 2010). The extensive hESC death is even further exacerbated upon passaging of cells by enzymatic methods that entail dissociation of cell colonies to single cells (Chen et al., 2010; Ohgushi et al., 2010; Watanabe et al., 2007), resulting in a very low single-cell cloning efficiency (typically <1%) (Enver et al., 2005; Harrison et al., 2007). The low cloning efficiency is at least partly due to an excessive apoptosis of cells upon dissociation (Chen et al., 2010; Ohgushi et al., 2010), but the discrepancy in the number of cells surviving the initial plating and the overall cloning efficiency suggests that critical restriction points exist between initial plating and when robust colony formation is established. The nature of these further restrictions remains unknown, although we have previously posited that cell:cell contact provides crucial signals, perhaps mediated by the NOTCH system, for the survival and proliferation of undifferentiated human pluripotent stem cells (Andrews et al., 1982; Fox et al., 2008).

The severe reduction in hESC numbers during culture has been proposed to impose a strong selection pressure on cells for genetic variants that permit escape from the normal restrictions for self-renewal (Amps et al., 2011; Baker et al., 2007; Draper et al., 2004). Indeed, nonrandom karyotypic changes, which might be indicative of such variants, are frequently observed in hESC cultures (Amps et al., 2011). We have previously termed such karyotypically abnormal hESC culture "adapted" cells because they present substantially more robust population growth patterns (Enver et al., 2005). The issue of adaptation has raised concerns about the safety of hESCs in regenerative therapies and has brought to the forefront the need for detection of adapted cells arising in culture. One of the major hallmarks of adapted cells is improved cloning efficiency (Enver et al., 2005; Harrison et al., 2007), but the interpretation of the origin of differences in cloning efficiencies of normal and adapted cells is complicated by the fact that in the cloning assays as routinely practiced, single cells are scored for their ability to form colonies after several days of growth, and no account is usually taken of how different sublineages from the single founder cells contribute to the final colony. Here, we have used a combination of cell sorting, time-lapse video microscopy, single-cell tracking, and modeling techniques to characterize the bottlenecks that prevent clonal expansion of normal hESCs and elucidate how these are overcome by adapted cells.

RESULTS

Bottlenecks in Postplating Survival and Re-entry into the Cell Cycle Are Relieved upon Culture Adaptation

We compared the behavior of normal and adapted sublines of two well-characterized hESC lines, H7 and H14 (Thomson et al., 1998): H7.s14 and H14.s9 are karyotypically normal sublines, whereas H7.s6 and H14.BJ1 are later passage, adapted sublines, with karyotypic changes and a markedly increased population growth rate (Baker et al., 2007; Draper et al., 2004; Enver et al., 2005). To ensure that the time-lapse analysis was done with undifferentiated stem cells rather than their differentiated derivatives, we first sorted the cells on the basis of their expression of the cell surface antigen, SSEA3, a particularly sensitive marker of the undifferentiated state (Draper et al., 2002; Enver et al., 2005). Dissociated normal and adapted cells were plated at low density and filmed from the time of plating for 72 hr (Figures 1A and 1B; Movies S1 and S2 available online). At the end of 72 hr, cells were fixed and stained for SSEA3 (using a different secondary antibody to the initial sort) to determine whether they remained undifferentiated (Figures 1A and 1B). A majority of cells remained SSEA3 positive, and there was no difference in the proportion of cells that expressed SSEA3 between normal and adapted cells (Figure 1C).

Cells were tracked from movies to record a detailed history of their survival and mitoses (Figures 2A and 2B; Movies S1 and S2). The lineage trees reconstructed from time-lapse movies of normal and adapted H7 cells were markedly different (Figures 2A and 2B). Strikingly, adapted cells showed better recovery after plating, with 82% ± 11% of the initial population still alive after 12 hr (Figure 2C) in contrast to 50% ± 5% of the initial normal population. The Rho-associated, coiled-coil containing protein kinase 1 (ROCK) inhibitor Y-27632 (Watanabe et al., 2007) improved the survival of normal cells but had no effect on the survival of adapted cells after 12 hr (Figures 2C and S1A–S1D; Movies S3 and S4). Although Y-27632 had no apparent beneficial effects on the individual adapted cells tracked in time-lapse movies, it increased the number of attached cells after plating and hence the overall cell number at 24 hr postplating (Figure S1E). We observed similar differences in the survival after plating between normal and adapted H14 hESCs (Figure S2A).

Of the cells that survived plating, a smaller proportion of the normal than the adapted H7 hESCs reentered the cell cycle and divided (31% ± 8% in normal cells compared to 66% ± 20% in adapted cells) (Figure 2D), indicating a second bottleneck in the expansion of the normal cells. Normal and adapted H14 cells displayed the same trend in reentry into the cell cycle (Figure S2B). This second bottleneck was overcome by treatment of normal cells with the ROCK inhibitor, Y-27632 (Figure 2D). However, the time from plating to the first division was shorter for the surviving, mitotic normal cells (median 8 hr) than for the adapted cells (median 15 hr), or for the normal or adapted cells treated with Y-27632 (medians 12 and 16 hr, respectively) (Figure 2E). H14 normal and adapted cells displayed a similar pattern (Figure S2C). On the other hand, in subsequent cell divisions, the cell-cycle times for the normal and adapted cells, whether or not exposed to Y-27632, were similar, although there were small differences (Figures 2F and S2D). One explanation for the difference in the time until the first division would be that only normal cells that are in the later stages of the cell cycle are able to divide after plating. Indeed, we conducted experiments with cells sorted based on SSEA3 expression and DNA content to show that the majority of dividing normal cells come from the G2 fraction (Figure 3A). However, the same appeared to be true for the adapted cells (data not shown), and in cloning efficiency experiments, the G2 cells were more efficient in creating colonies than G1 in both sublines: the cloning efficiency ratio of G2/G1 in triplicate experiments was 3.1 ± 0.9 in normal cells and 1.8 ± 0.6 in adapted cells (no statistical difference) (Figure 3B). SSEA3 staining at the end of the cloning assay revealed no difference in the percentage of undifferentiated cells between colonies formed from cells in G1 or G2 at the time of plating (Figure 3C), indicating that in the conditions we used, the G1 cells are more sensitive to death (rather than differentiation) signals upon plating than the G2 cells. Furthermore, no significant difference between the ratios of G2/G1 cloning efficiencies in normal and adapted cells suggests that the difference in time to first division between normal and adapted cells is not due to stage in the cell cycle but rather to the normal cells reentering cell cycle prematurely before they had recovered from the disruption of harvesting and reseeding. Evidently, ROCK inhibitor is able to prevent this premature re-entry into cell division (Figure 3B). We confirmed that the observed bottlenecks were not a result of the change in culture conditions (i.e., transferring cells from mouse embryonic fibroblasts to Matrigel), by analyzing survival of cells that were serially passaged in Matrigel with mTeSR for three to four passages and then filming them under the same culture conditions (Figures S3A and 3B).

Cell Death Postdivision Further Limits the Clonal Expansion of Normal hESCs but Is Reduced upon Culture Adaptation

Another factor that can influence the population growth and colony formation is the rate of cell loss during subsequent culture. Following division, daughter cells can conform to one of the three patterns: both daughter cells survive and give progeny (SS, survival-survival); one

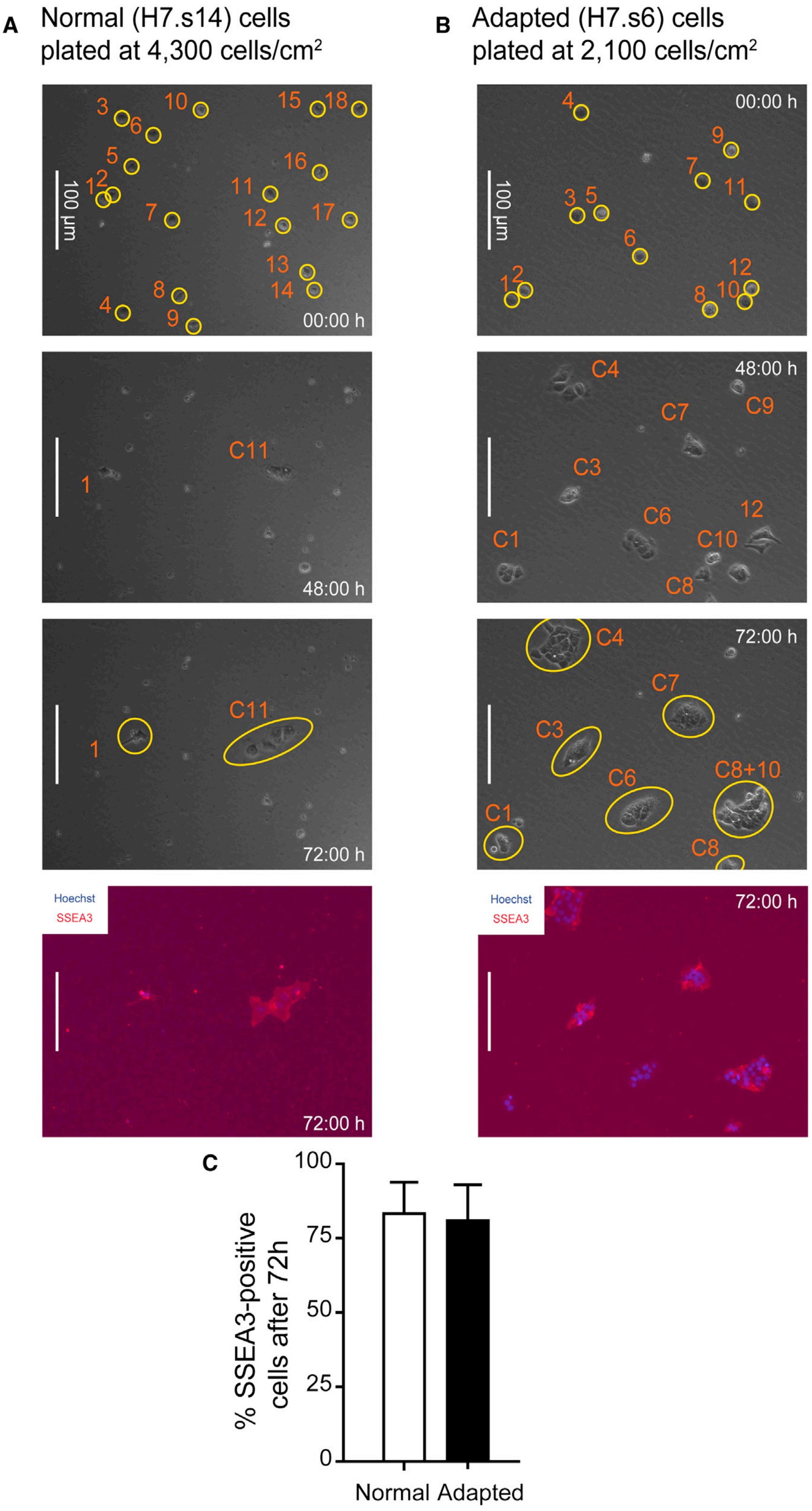

Figure 1. Single-Cell Behavior of Normal H7.s14 and Adapted H7.s6 Cells Investigated by Time-Lapse Analysis

(A) Time-lapse analysis of SSEA3-sorted normal cells plated at low density. Still images are taken from Movie S1. Cells labeled 1–18 are circled in the uppermost panel, and their corresponding lineage trees are shown in Figure 2A. The only colony present at 72 hr is labeled C11 (originating from cell 11). The lowermost panel shows staining for SSEA3 (red) and nuclei counterstained with Hoechst 33342 (blue) at the end of the filming.

(B) Time-lapse analysis of SSEA3-sorted adapted cells plated at low density. Still images are taken from Movie S2. Cells labeled 1–12 are circled in the uppermost panel, and their corresponding lineage trees are shown in Figure 2B. The majority of colonies present at 72 hr were formed from single cells (labeled C1–C8 according to the originating cell), but occasionally, we noted satellite colonies being formed (cell 8 gives rise to progeny in two colonies labeled C8 and C8+10) as well as rare mixing of nearby colonies (progeny from cells 8 and 10 found in the mixed colony labeled C8+10). The resulting colonies were stained for SSEA3 (red) and nuclei counterstained with Hoechst 33342 (blue) at the end of the filming (lowermost panel).

(C) Proportions of normal and adapted cells positive for SSEA3 at the end of the time-lapse experiments (72 hr). Results are the mean of triplicate independent experiments ± SD.

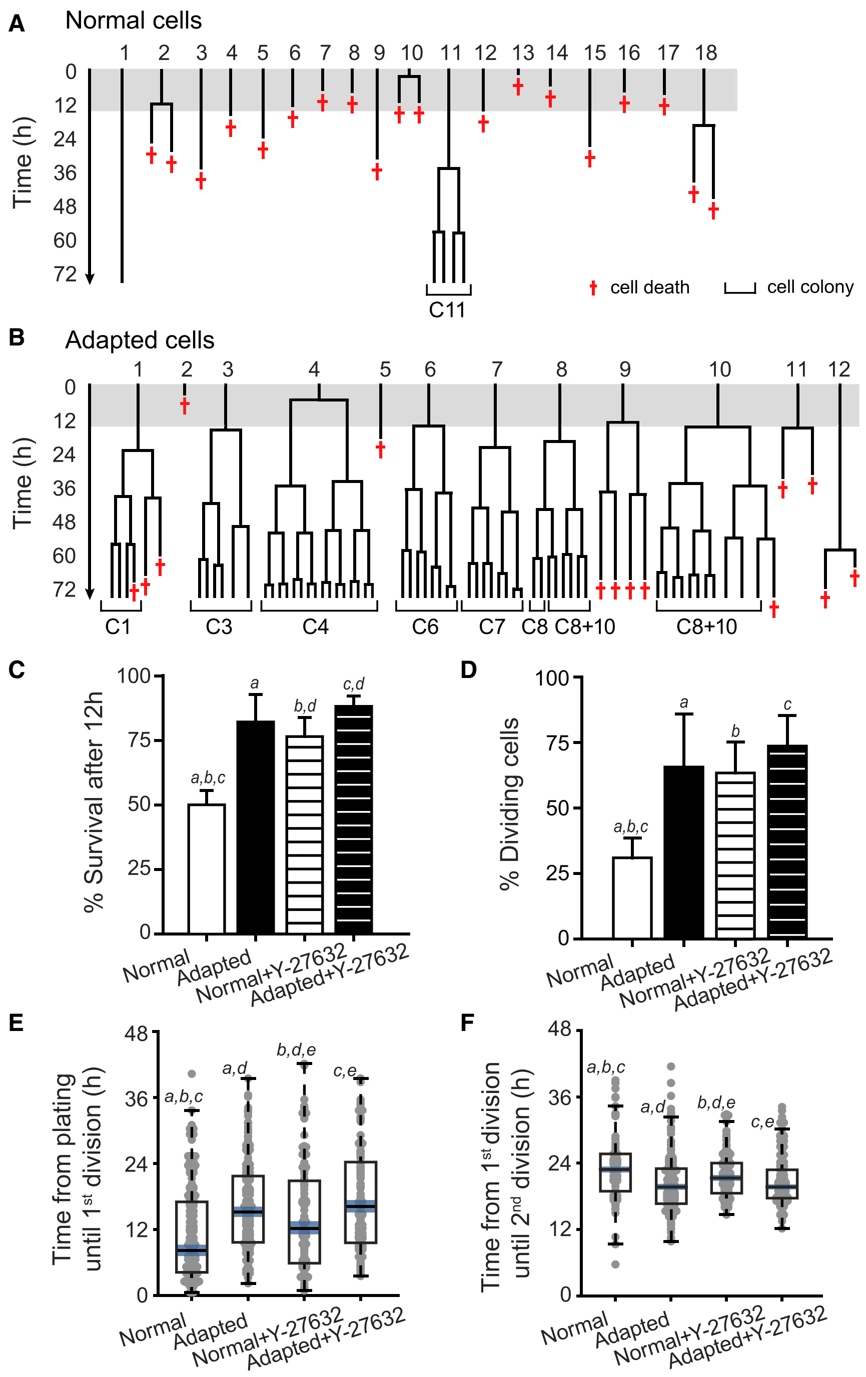

Figure 2. Bottlenecks Limiting the Growth of Normal H7.s14 hESCs Revealed through Time-Lapse Imaging

(A) Lineage trees of normal cells reconstructed from Movie S1 filmed over 72 hr from the time of plating the cells. Numbers correspond to cells circled in Figure 1A. Red crosses indicate the cell death. Label C11 denotes a colony arising from cell 11, which is also highlighted in Figure 1A. Gray-shaded area indicates the first 12 hr postplating.

(B) Lineage trees of adapted (H7.s6) cells reconstructed from Movie S2 filmed over 72 hr from the time of plating the cells. Numbers correspond to cells circled in Figure 1B. Red crosses indicate the cell death. Labels C1–C8 denote clonal colonies, whereas label C8+10 denotes a colony arising from merged progeny of cells 8 and 10, also highlighted in Figure 1B. Gray-shaded area indicates the first 12 hr postplating.

(C) The percentage of cells from the initial plated population that survived the first 12 hr after plating for normal, adapted, and Y-27632-treated normal and adapted cells. Results are the mean of quadruplicate independent experiments ± SD (a–d represent Student's t tests, left tail; p values: a, <0.001; b, <0.001; c, <0.0001; d, <0.05). Data consist of 150–205 cells per culture.

(D) The percentage of cells that divided in the normal, adapted, and Y-27632-treated normal and adapted cultures plated at low density. Results are the mean of quadruplicate independent experiments ± SD (a–c represent Student's t tests, left tail; p values: a, <0.01; b, <0.01; c, <0.001). Data consist of 150–205 cells per culture.

(E) The cell-cycle re-entry time following plating for normal, adapted, and Y-27632-treated normal and adapted cells. Results are box plot representations of distributions from a minimum of four independent experiments with median line and 95% median confidence intervals (blue); observations (gray dots) are shown stretching horizontal by frequency (a–e represent Kruskal-Wallis tests; p values: a, $<10^{-8}$; b, <0.01; c, $<10^{-8}$; d, <0.05; e, <0.01). Data consist of 110–170 cells per culture.

(F) Cell-cycle time in subsequent divisions for normal, adapted, and Y-27632-treated normal and adapted cells. Results are box plot representations of distributions from a minimum of four independent experiments with median line and 95% median confidence intervals (blue); observations (gray dots) are shown stretching horizontal by frequency (a–e represent Kruskal-Wallis tests; p values: a, <0.0001; b, <0.05; c, <0.001; d, <0.05; e, <0.001). Data consist of 70–170 cells per culture.

daughter cell survives and gives progeny, the other one dies (SD, survival-death); or both daughter cells die without dividing (DD, death-death) (Figure 4A). Although all three scenarios occurred during the regrowth of normal and adapted cells, normal cell divisions were strongly biased toward both daughter cells dying following the first division

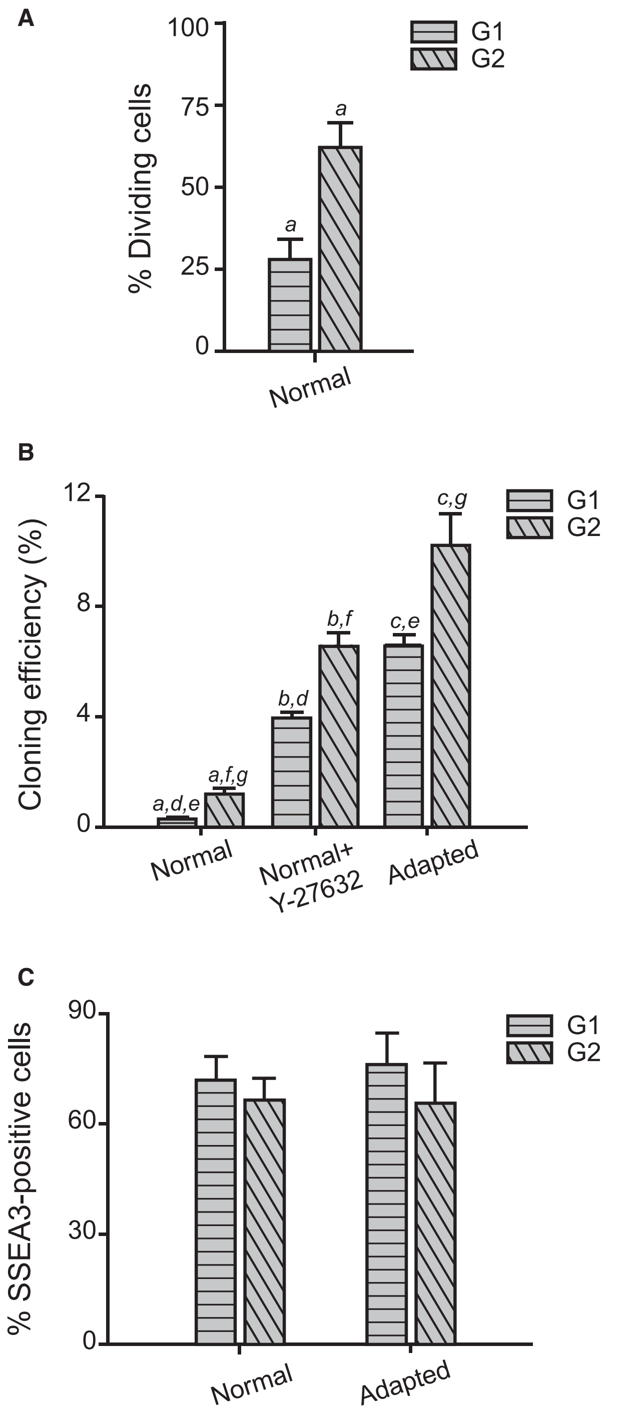

Figure 3. Cloning Efficiencies of Normal H7.s14 and Adapted H7.s6 Cells Plated in G1 or G2 Cell-Cycle Phase

(A) The percentage of cells that divided in the normal SSEA3-sorted population fractionated into G1 and G2 based on the DNA profile, plated at low density, and analyzed using time-lapse imaging. Results are the mean of triplicate independent experiments ± SD (a represents Student's t test, left tail; p value: <0.01). Data consist of 160–320 cells per fraction.

(B) A representative example of cloning efficiencies of SSEA3-sorted normal and adapted cells fractionated into G1 and G2 based on the DNA profile, analyzed 7 days after plating. Results are the mean of triplicate wells from the same experiment ± SD (a–g represent Student's t tests, left tail; p values: a, $<10^{-5}$; b, <0.001; c, <0.01; d, $<10^{-9}$; e, $<10^{-8}$; f, $<10^{-6}$; g, $<10^{-6}$).

(C) Proportions of normal and adapted cells expressing SSEA3 7 days after plating SSEA3-sorted cells fractionated into G1 and G2 based on the DNA profile. Results are the mean of triplicate wells from the same experiment ± SD.

postplating (DD, 62% ± 1%) compared to both adapted daughter cells surviving (SS, 71% ± 5%) (Figure 4B). A similar trend was identified in the H14 cell pair (Figure S2E) and in H7 cells after serial passaging on Matrigel (Figure S3C). Following the second division, the proportion of divisions leading to both daughter cells dying was reduced in normal cells compared to the first division postplating but, nonetheless, remained high (DD, 40% ± 9%), demonstrating that persistent cell death is an inherent feature of hESC cultures but is reduced by culture adaptation (Figures 4B and 4C). Treatment of normal cells with the ROCK inhibitor Y-27632 shifted the bias in cell division from death (DD) to survival (SS) of both daughter cells, with no further improvement in Y-27632-treated adapted cultures (Figures 4B and 4C). The number of surviving normal daughter cells declined rapidly in the 12 hr following the first division (Figure 4Da), with death occurring relatively early after the division (median 8 hr) (Figure 4Ea). Although the survival of normal daughter cells was improved following the second division (Figure 4Db), daughter cells continued to die sooner following division compared to the adapted cells (Figure 4Eb).

Direct Cell:Cell Contact Rather Than Diffusible Factors Enhances Survival and Re-entry into the Cell Cycle of Normal hESCs

High-cell density is known to enhance hESC viability, and hESCs are routinely passaged as clumps that increase their survival (Figures 5A–5C; Movie S5), cell-cycle re-entry (Figure 5D; Movie S5), and survival after division (Figure 5E; Movie S5) compared to plating of single cells. However, it remains unclear if high-cell density plays a role in culture adaptation and whether it is mediated by direct cell:cell contact or a diffusible factor produced by the cells. When normal and adapted cells were plated at increasing cell densities, the adapted cells gave rise, as expected, to larger populations compared to the normal cells at all plating densities after 5 days of growth (Figure 6A). However, whereas the adapted cells showed a linear increase in cell numbers in respect to the plating density, the normal cells exhibited a nonlinear (quadratic) relationship between cell-plating density and population growth. Thus,

increasing cell-seeding density enhanced the growth of normal cells, but not that of the adapted ones.

Given the growth advantage that high density conferred on normal cells, we used time-lapse microscopy to examine whether it alleviated bottlenecks early after plating. Higher plating density of SSEA3-sorted single cells did improve the initial survival of normal hESCs (Figure 6B) and also increased the proportion of cells that reentered the cell cycle (Figure 6C). Additionally, the bias in cells dividing early after plating evident in the low-density cultures of normal cells was not present at high density (Figure 6D). The cell-cycle duration in subsequent divisions was similar in both plating density conditions (Figure 6E).

Overcoming the bottlenecks by plating of cells at a higher density could be due to prosurvival signals transmitted via cell:cell contacts or due to higher concentrations of diffusible prosurvival factors produced by the cells. At our chosen high-plating density (10,600 cells/cm^2), not all cells came into contact with others, allowing us to compare the survival and re-entry into the cell cycle of cells that remained isolated as opposed to cells that came into contact with other cells. Strikingly, the normal cells that made contact with other cells were more likely to divide than the isolated cells, which showed similar behavior to cells plated at a low density (Figure 6F). Nevertheless, some isolated cells did divide, but similar to low-density cultures, their time until the first division was low (median 8 hr), in contrast to cells in contact for which the median time to division was approximately 17 hr postplating (Figure 6G), closer to that of the adapted cells. Given that the behavior of both groups of cells was analyzed in the same culture, these results are consistent with the hypothesis that direct cell:cell contact rather than factors present throughout the culture environment promotes survival and mitosis of normal cells.

The Proliferative Fraction of Normal but Not Adapted Cells Is Solely Associated with Enhanced Motility

Because cell:cell contact benefits normal cells, the growth advantage of the adapted cells could be explained if they were to exhibit enhanced motility. To test this, we analyzed the cell movement in the normal and adapted H7 and H14 cultures before division (<12 hr postplating) by calculating the mean squared displacement (msd) with respect to time (t). The msd is a measure related to the average distance traveled by cells and is proportional to $\sim t^{\beta}$ in the long time limit (Dieterich et al., 2008), where the parameter $0 < \beta \leq 2$ distinguishes between different stochastic diffusion processes. For a random walk, or Brownian-like motion, msd has a linear trend, with $\beta = 1$, whereas non-Brownian motion types are defined when msd deviates from linearity, i.e., $\beta \neq 1$ (Metzler and Klafter, 2000). The motility analysis of plated normal and adapted hESCs highlighted the existence of three subpopulations, exhibiting different dynamics: (1) Brownian-like cells in which the trajectories resembled a random walk (msd$\sim$t); (2) superdiffusive cells (msd$\sim t^{\beta}$, $\beta > 1$) in which the movement of cells was nonrandom; and (3) subdiffusive cells (msd$\sim t^{\beta}$, $\beta < 1$), which were essentially nonmotile (Movie S6).

We analyzed the msd data of the motile cells, both superdiffusive and Brownian-like, by estimating fractional diffusion models. There were no notable differences between the parameters of the diffusion models estimated from trajectories of Brownian-like normal and adapted cells (Figures S4A and S4B; Tables S1 and S2). However, for the adapted superdiffusive cells, the diffusion coefficient was double that of normal superdiffusive cells (Tables S1 and S2), indicating that they tended to travel further from their point of origin (Figures 7A and S4C). Nevertheless, at low-plating density, this made little difference because the average cell:cell distance was approximately 142 μm (Figure S5A), and 95% of normal and adapted cells, whether Brownian-like or superdiffusive, traveled at most only up to 43 μm during 12 hr postplating. Indeed, observations from time-lapse imaging showed that none of the normal and only 3% of adapted cells acquired additional cell contacts through movement over the 12 hr postplating period (Figure 7B), so that the enhanced growth of the adapted cells appears not to be a result of increased cell contacts mediated by enhanced motility. The small increase in contact observed in the adapted culture after 12 hr postplating is due to 95% of superdiffusive adapted hESCs traveling up to 12 μm further than 95% of superdiffusive normal hESCs.

Despite the lack of additional cell contact through cell movement, the msd for the normal cells that gave rise to colonies present at 72 hr postplating closely matched that of superdiffusive normal cells (Figures 7C and S4D). This suggests that the enhanced ability of superdiffusive normal hESCs to proliferate is an intrinsic property of this subpopulation rather than a consequence of increased motility mediating contacts with other cells. In contrast, the msd characteristic of colony-forming adapted cells was intermediate, indicating that, in this case, colonies formed from both Brownian-like and superdiffusive cells (Figures 7D and S4E). Notably, in both the normal and adapted cultures, colonies did not form from nonmotile cells.

Motility may reflect a response to diffusible signaling factors. Because the proliferation of normal cells was promoted by high-cell density conditions, where the concentrations of diffusible factors produced by the cells would be expected to be higher, we investigated whether these conditions further enhanced cell movement. When we analyzed normal H7 cells plated at high density, the msd of both Brownian-like and superdiffusive cells increased compared to the low-density condition (Figure S4F).

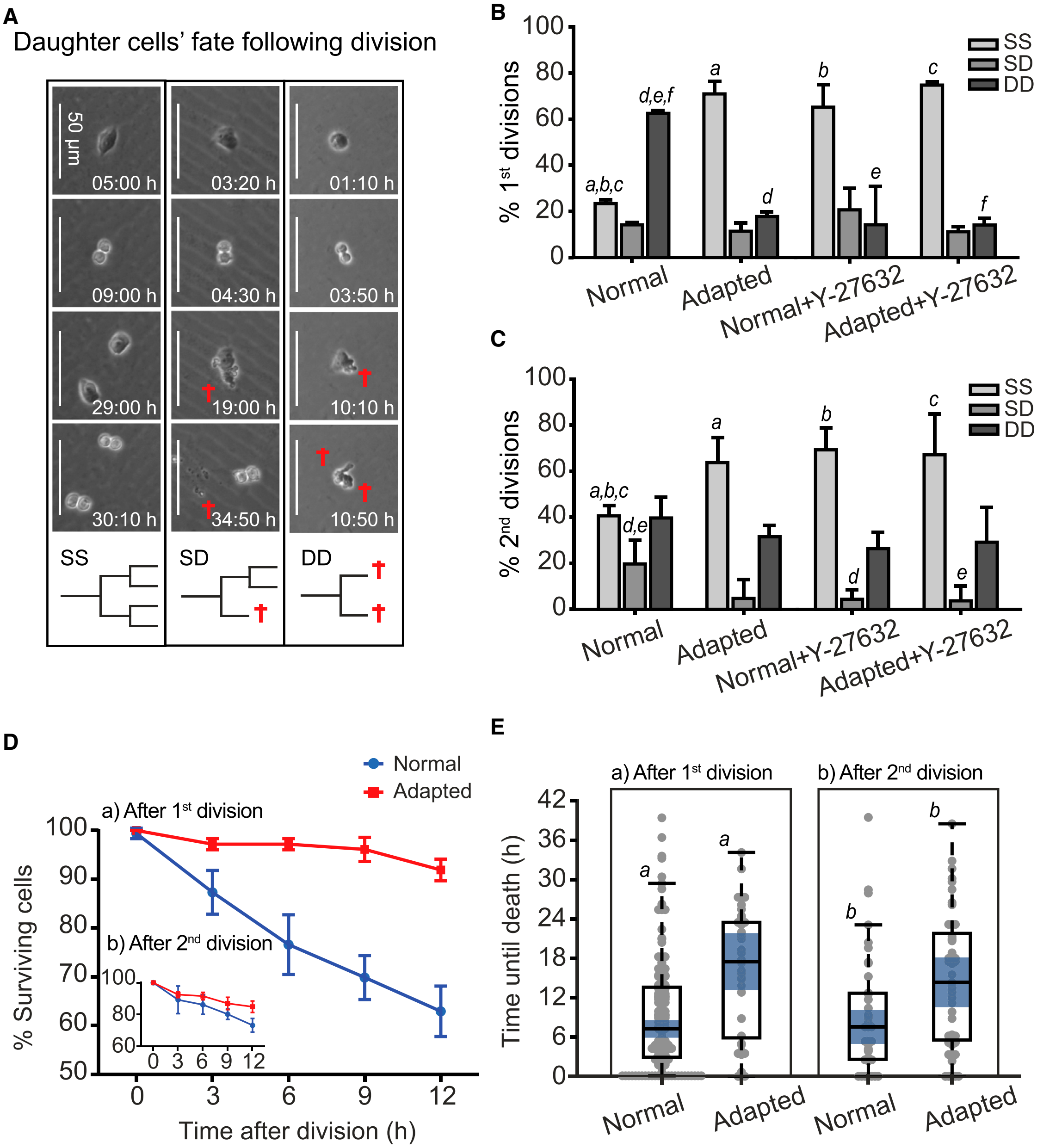

Figure 4. Survival of Normal H7.s14 and Adapted H7.s6 Daughter Cells Postdivision Characterized through Time-Lapse Imaging

(A) Possible scenarios of daughter cells' fate following division: (1) both daughter cells divide (SS), (2) only one daughter cell divides (SD), and (3) both daughter cells die without dividing (DD). Still images from time-lapse movies exemplify the three scenarios. Red crosses indicate cell death. Time after plating is indicated in each of the still images.

(B) Percentage of SS, SD, and DD types of division occurring in normal, adapted, and Y-27632-treated normal and adapted cells that divided for the first time after plating. Results are the mean of triplicate independent experiments ± SD (a–f represent Student's t tests, a–c left tail, d–f right tail; p values: a, 0.0001; b, <0.001; c, $<10^{-5}$; d, $<10^{-5}$; e, <0.01; f, <0.0001). Data consist of 60–110 cell divisions per culture.

(C) Percentage of SS, SD, and DD types of division occurring in normal, adapted, and Y-27632-treated normal and adapted cells that divided for the second time after plating. Results are the mean of triplicate independent experiments ± SD (a–d represent Student's t tests, a–c left tail, d–e right tail; p values: a, <0.05; b, <0.01; c, <0.05; d, <0.05; e, <0.05). Data consist of 35–65 cell divisions per culture.

(D) Percentage of surviving normal and adapted cells over a 12 hr time period following the first (a) and the second division postplating (b). Results are the mean of triplicate independent experiments ± SD. Data consist of 145–250 cells per culture.

(legend continued on next page)

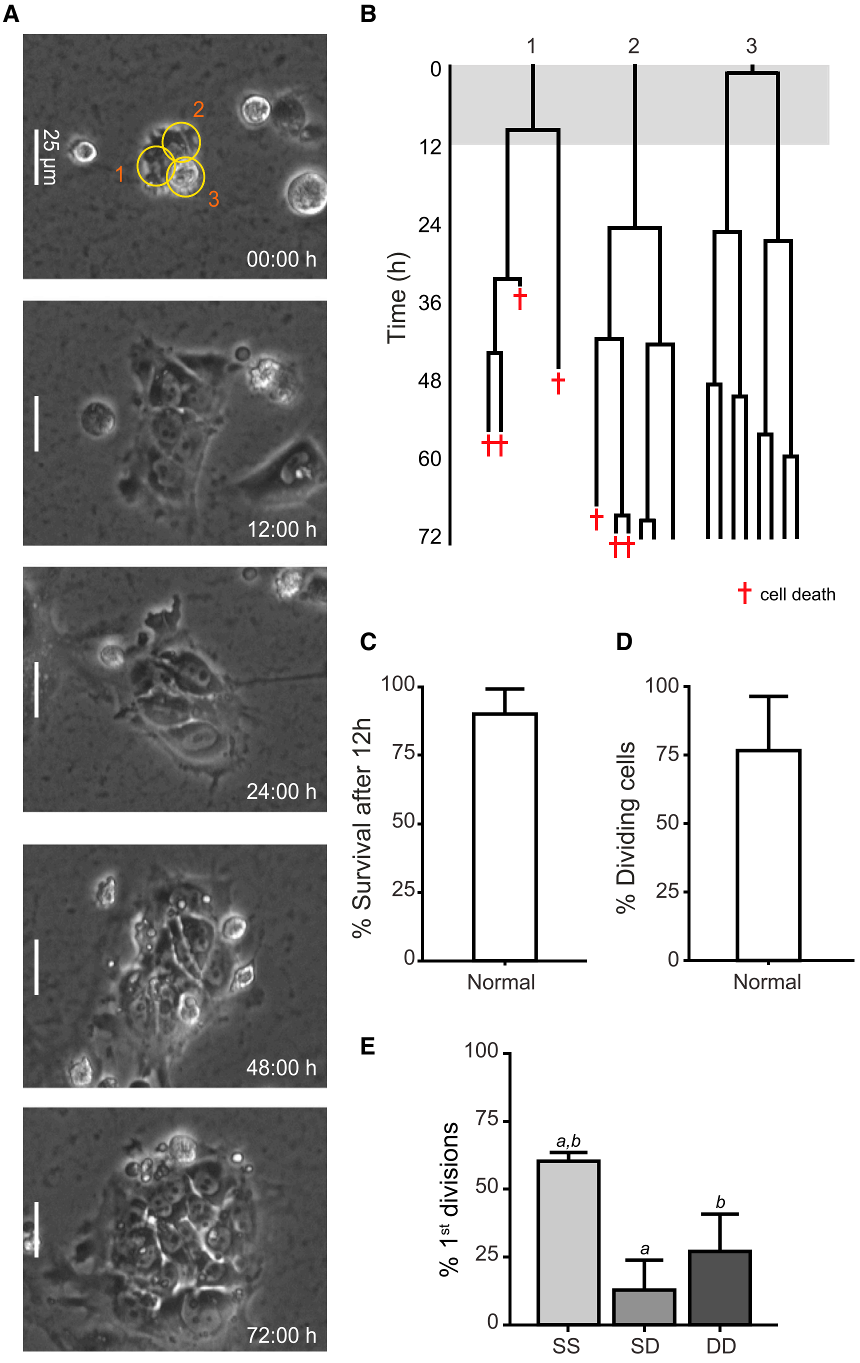

Figure 5. Time-Lapse Analysis of Normal H7.s14 Cells Passaged in Small Clumps

(A) A three-cell clump of normal cells obtained by dissociation of cells with nonenzymatic solution. Still images are taken from Movie S5. The cells in the clump are labeled 1–3 and circled in the uppermost panel, and their corresponding lineage trees are shown in (B).

(B) Lineage trees of normal cells passaged in small clumps (reconstructed from Movie S5) filmed over 72 hr from the time of plating the cells. Numbers correspond to cells circled in (A). Red crosses indicate the cell death.

(C) The percentage of normal cells passaged as clumps that survived the first 12 hr post-plating. Results are the mean of triplicate wells from the same experiment ± SD. Data consist of approximately 40 cells.

(D) The percentage of normal cells passaged as clumps that divided after plating. Results are the mean of triplicate wells from the same experiment ± SD. Data consist of approximately 40 cells.

(E) Percentage of first divisions following which both daughter cells divide further (SS), one daughter cell divides whereas the other dies (SD), or both daughter cells die (DD) in normal cells passaged as clumps. Results are the mean of triplicate wells from the same experiment ± SD (a and b represent Student's t tests, right tail; p values: a, 0.001; b, <0.01). Data consist of approximately 25 divisions.

However, the increased density disproportionately enhanced the motility of superdiffusive cells (diffusion coefficient increased 5-fold, and thermal speed increased 2-fold) compared to Brownian-like cells (diffusion coefficient increased 2-fold, and thermal speed was unchanged) (Table S1). This suggests that cultures do produce factors that enhance the motility of cells and that superdiffusive normal cells are more responsive to such factors.

(E) The time from division until the observed cell death in time-lapse movies of normal and adapted cells, corresponding to the first (a) and the second division (b) postplating. Results are box plot representations of distributions from triplicate independent experiments with median line and 95% median confidence intervals (blue); observations (gray dots) are shown stretching horizontal by frequency (a and b represent Kruskal-Wallis tests; p values: a, <0.0001; b, <0.01). Data consist of 40–160 cells per culture.

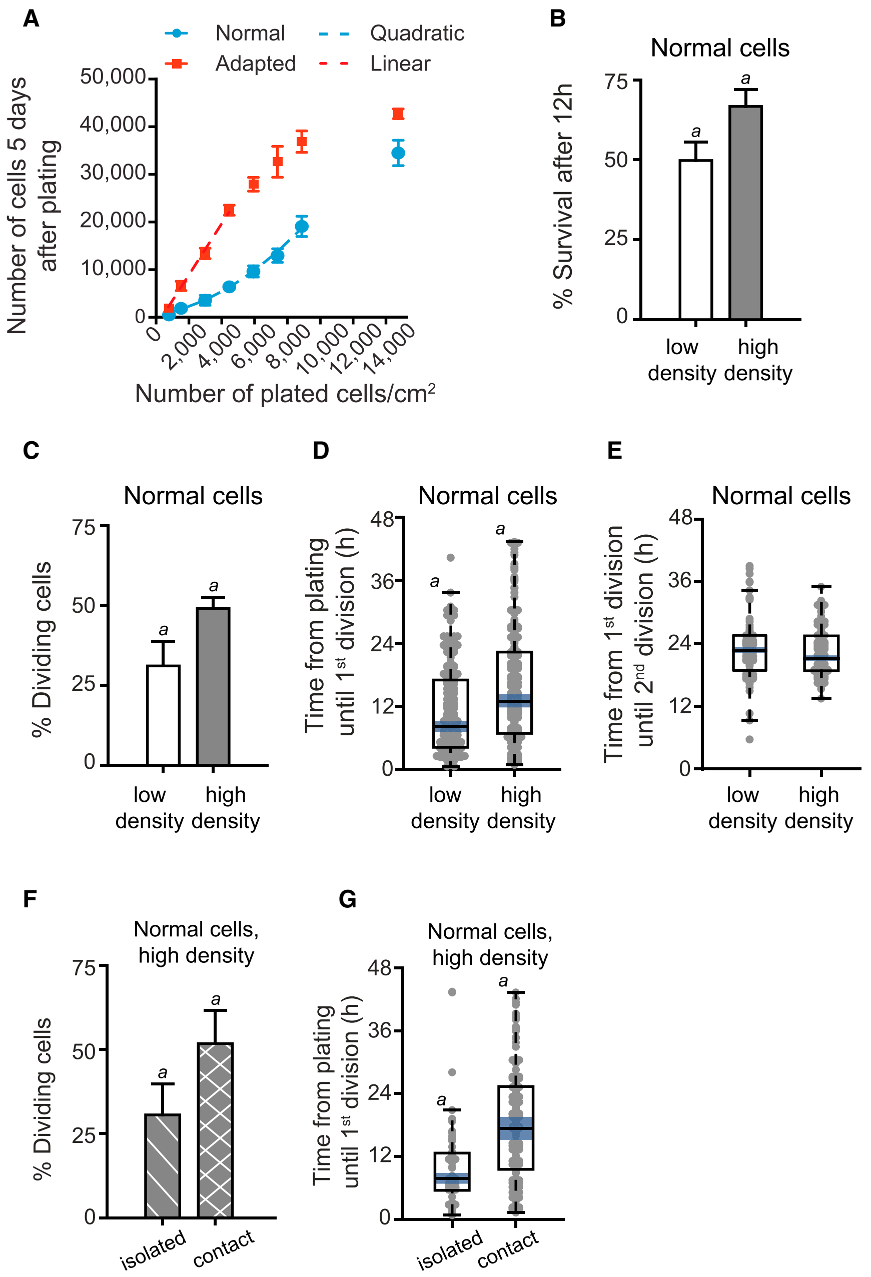

Figure 6. The Effect of High-Plating Density on the Survival of Normal H7.s14 hESCs

(A) Effect of increasing cell-plating density on the numbers of normal (H7.s14) and adapted (H7.s6) cells 5 days postplating. The dashed blue line indicates a quadratic trend of the normal curve, and the dashed red line indicates the linear trend of the adapted curve. Due to the limited area of the well, at high-plating densities, cells form a monolayer, and the growth of the adapted population plateaus. Results are the mean of six wells from the same experiment ± SD.

(B) Percentage of surviving normal hESCs 12 hr postplating at low and high cell-plating density. Low-density normal data are also presented in Figure 2C. Results are the mean of a minimum of triplicate independent experiments ± SD (a, Student's t test, left tail; p value: <0.01). Data consist of 205–365 cells per culture condition.

(C) Percentage of normal cells that divided from the initial plated population at low and high cell-plating density. Low-density normal data are also presented in Figure 2D. Results are the mean of a minimum of triplicate independent experiments ± SD (a, Student's t test, left tail; p value: <0.01). Data consist of 205–365 cells per culture condition.

(D) The cell-cycle re-entry time following plating for normal cells plated at low and high cell-plating density. Low-density normal data are also presented in Figure 2E. Results are a box plot analysis of a minimum of three independent experiments with median and 95% median confidence bounds (blue) and observations (gray dots) shown stretching horizontal by frequency (a, Kruskal-Wallis test; p value: <0.0001). Data consist of 175–180 cell divisions per culture condition.

(E) Cell-cycle time for normal cells plated at low and high cell-plating density. Low-density normal data are also presented in Figure 2F. Results are box plot representations of distributions from a minimum of triplicate independent experiments with median line and 95% median confidence intervals (blue); observations (gray dots) are shown stretching horizontal by frequency. Data consist of 70–85 cells per culture condition.

(F) Percentage of normal cells that divided when present in culture as isolated cells (and without ever making contact with other cells after plating) versus the cells that divided after having come into direct contact with other cells, analyzed within the same culture. Results are the mean of triplicate independent experiments ± SD (a, Student's t test; p value: <0.05). Data consist of 365 cells.

(G) Cell-cycle re-entry postplating for normal cells that divided when present as isolated cells versus the cells that divided after having come into direct contact with other cells, analyzed within the same high-cell density culture. Results are box plot representations of distributions from triplicate independent experiments with median line and 95% median confidence intervals (blue); observations (gray dots) are shown stretching horizontal by frequency (a, Kruskal-Wallis test; p value: $<10^{-7}$). Data consist of 45–130 cells per category.

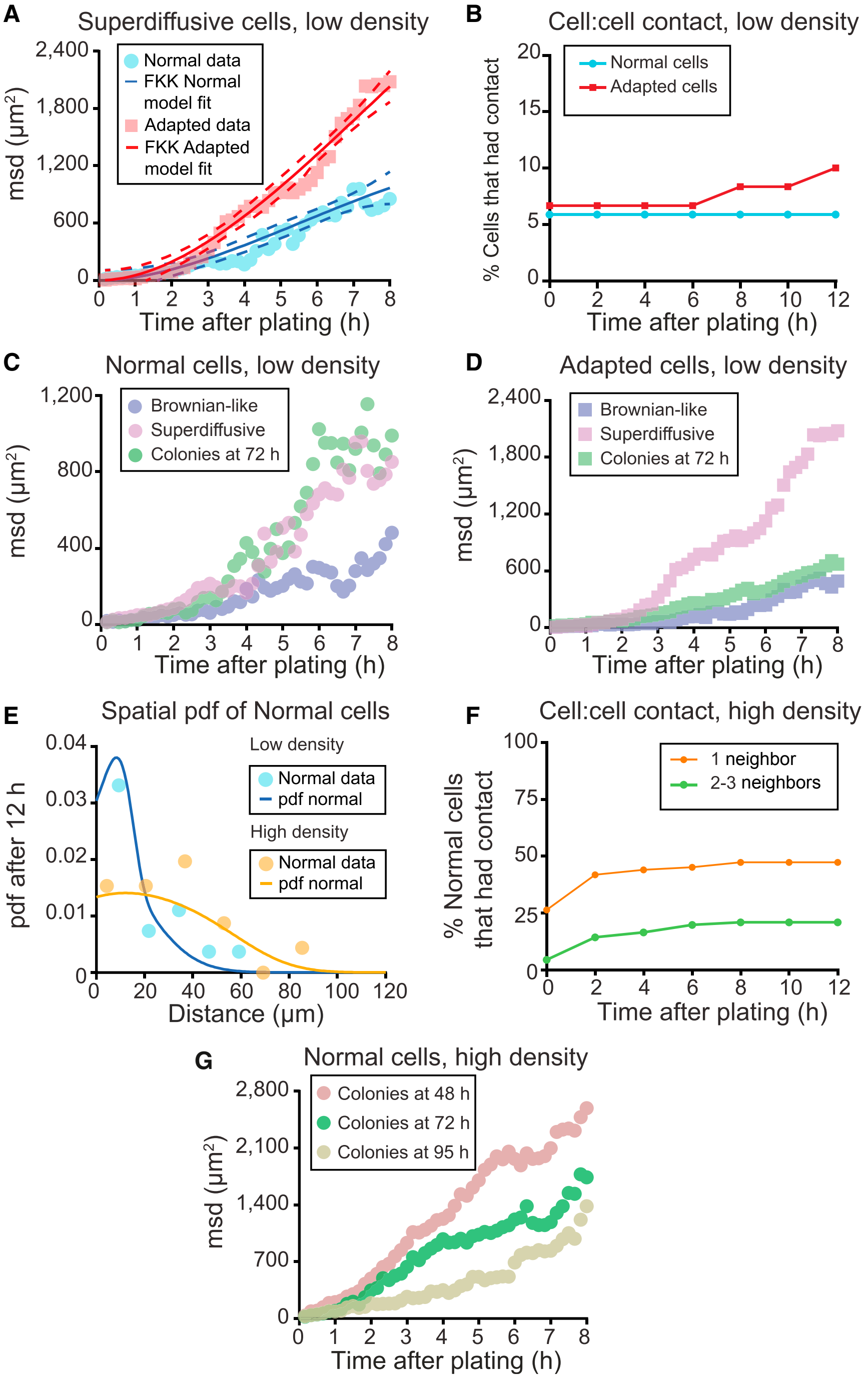

Figure 7. Motility Analysis for Normal H7.s14 and Adapted H7.s6 Cells

(A) msd for trajectories of superdiffusive normal and adapted cells at low-plating density. Only motile cells were analyzed, with the msd for Brownian-like cells included in Figure S4A. Markers represent ensemble average msd, solid lines are trends predicted with the FKK model corresponding to superdiffusive motion, and dashed lines indicate the 95% prediction bounds. The rise in the msd for adapted superdiffusive cells indicates an increased ability to travel in culture compared to the trend in normal superdiffusive cells. Data consist of 11–13 cells per culture.

(B) Percentage of cells plated at low density that had come into direct cell:cell contact with at least one other cell at any time up to 12 hr postplating. Data consist of 65 cells per culture.

(C) Msd calculated from trajectories of normal cells exhibiting Brownian-like (also presented in Figure S4A) and superdiffusive (also presented in A) movement. Markers represent ensemble average msd from real data in specified categories.

(D) Msd calculated from trajectories of adapted cells exhibiting Brownian-like (also presented in Figure S4A) and superdiffusive (also presented in A) movement. Markers represent ensemble average msd from real data in specified categories. Data consist of 55 cells forming colonies.

(E) Probability of normal cells plated at low and high density to travel any given distance from origin after 12 hr postplating calculated as spatial pdf. Markers represent empirical pdf of distance traveled from origin after 12 hr, and solid lines represent predicted trend of spatial pdf. The trend is calculated assuming an equally weighted mixture of Brownian-like and superdiffusive normal cells. Data consist of approximately 20 cell displacements per culture condition.

(F) Percentage of normal cells plated at high density that had come into cell:cell contact with nearby cells at any time up to 12 hr postplating, arranged by number of neighbors. Data consist of 91 cells.

(G) Msd calculated from trajectories of normal cells that gave rise to colonies present at 48, 72, and 95 hr postplating. Markers represent ensemble average msd from real data in specified categories. Data consist of 30–80 cells per category.

The probability density function (pdf) derived from the diffusion models shows that at high-plating density, normal cells traveled further from their origins compared to low-density conditions (Figure 7E). Specifically, at high density, the average cell:cell distance was reduced to approximately 47 μm, and 95% of normal cells, whether Brownian-like or superdiffusive, traveled up to 76 μm over 12 hr postplating (Figure S5B). As a result, a significant proportion of normal (either superdiffusive or Brownian-like) cells acquired contacts through motility (Figure 7F). Analyzing the msd curves

for cells that gave rise to colonies present in the high-density normal culture revealed that whereas the contribution of superdiffusive cells was noticeable in early colonies (after 48 hr, Figure 7G), at later times, the colony-forming cells encompassed larger proportions of the slower Brownian-like cells (after 72 and 95 hr, Figure 7G). Thus, in addition to the survival of superdiffusive normal cells, high-plating density also allowed Brownian-like normal hESCs to be rescued through contact. A further consequence was that, at high-plating density, most of the colonies derived from the normal cells observed after 12 hr were from mixed lineages rather than clonal (Figure S5B; Movie S7). These observations suggest that the cell density-dependent growth of normal cells is related, at least in part, to their ability to form cell contacts through increased cell motility, a feature not required of adapted cells.

DISCUSSION

The maintenance and expansion of hESCs are compromised by their propensity to differentiate or to die, which inevitably affords conditions for the selection of genetic variants that provide a growth advantage by altering the cellular dynamics to favor self-renewal. Characterization of the selection pressures that drive the appearance of (epi)genetic variants of hESCs is pivotal for optimizing their maintenance conditions and minimizing the opportunity for expansion of mutated clones that could compromise safety and efficacy for applications in regenerative medicine. Several genetic changes, notably gains of sections of chromosomes 12, 17, and 20, do occur commonly on the long-term passaging of hESCs and appear to provide such growth advantages, seemingly independently of one another. One hypothesis is that overexpression of a gene(s) encoded by these regions of genomic gain provides the selective advantage, but so far, only the role of one such gene, *BCL2L1*, encoded on chromosome 20 has been definitively characterized (Avery et al., 2013), though other candidates have been proposed (Amps et al., 2011; Laurent et al., 2011). In the present study, we took a different approach by using time-lapse analysis of single cells to identify aspects of hESC behavior that tend to restrict growth and so would be targets for mutations that promote proliferation.

In the time-lapse data, only a very small proportion (approximately 3%) of the initially seeded, karyotypically normal cells, but a much greater proportion (approximately 19%) of the karyotypically abnormal, adapted cells, formed robust, continually growing colonies, which correlates well with previous observations of the differential plating efficiencies of normal and adapted hESCs (Enver et al., 2005; Harrison et al., 2007). Our results indicated at least three different bottlenecks to establishing proliferating cultures of hESCs after passaging, namely, initial survival of harvested and replated cells, their subsequent ability to reenter the cell cycle, and, most surprisingly, a continued propensity in those that do reenter the cell cycle to continue to die following subsequent cell divisions. All of these bottlenecks were alleviated in large measure in the adapted, genetically variant sublines of two independent hESC lines: H7 and H14.

Inhibition of ROCK, using the chemical inhibitor Y-27632, also alleviated each of the bottlenecks in the normal cells to a significant extent. Several studies have shown that the tendency of hESCs to die after dissociation to single cells is caused by actin-myosin contraction, mediated by nonmuscle myosin II (Chen et al., 2010; Ohgushi et al., 2010; Walker et al., 2010). ROCK mediates this contraction through phosphorylation of a number of target proteins, notably the myosin regulatory light chains (MLC2) of nonmuscle myosin II. Thus, chemical inhibition of ROCK isoforms ROCK I and II (e.g., with Y-27632, HA1007, or Pinacidil) (Barbaric et al., 2010; Watanabe et al., 2007), or their knockdown, allows cells to reattach to extracellular matrix and survive (Chen et al., 2010; Ohgushi et al., 2010). Our present data from time-lapse tracking of single cells treated with Y-27632 confirmed this, but we also found that the effects of ROCK inhibition extend beyond the rescue of cells at the time of plating. Specifically, the treatment of normal cells with Y-27632 increased the proportion of dividing cells among those that survived reseeding. Furthermore, Y-27632 reverted the apparent premature entry of normal cells into mitosis following replating. The signal(s) that drives normal cells to attempt early divisions is not clear, but it is likely that such a signal is triggered as a result of an inappropriate disruption of extracellular receptors by trypsin treatment during cell dissociation. One possibility is NOTCH receptors, which may regulate hESC proliferation and differentiation (Fox et al., 2008) and are implicated in cell-cycle progression of various cell types (Campa et al., 2008; Joshi et al., 2009), are activated by EDTA-trypsin treatment of cells (Rand et al., 2000). Furthermore, NOTCH activation has been observed to upregulate ROCK1 signaling (Yugawa et al., 2013), and therefore, inhibition of Rho-ROCK signaling could counteract inappropriate activation of NOTCH signaling during dissociation and replating of hESCs. Y-27632 also shifted the bias in cell division from both daughter cells dying after division to both daughter cells surviving, so that normal cells grown in the presence of Y-27632 formed many more robust long-term colonies like the adapted cells. Because completion of cell division requires transient detachment of cells from the extracellular matrix, and given the sensitivity of hESCs to dissociation-related apoptosis (Chen et al., 2010; Ohgushi et al., 2010), one possibility is that the same mechanism that results in the death of normal cells following

dissociation might also trigger their death when the cells detach to complete division.

Given the importance of ROCK signaling in hESC survival, it is likely that mutations affecting the activity or expression of multiple genes in this pathway could confer a competitive advantage to mutant cells. Indeed, neither of the adapted sublines of H7 or H14 was significantly influenced at any of the bottlenecks by Y-27632, whereas treatment of the normal sublines with Y-27632 closely phenocopied the adapted cells in each case, suggesting that adaptation in these H7 and H14 sublines may involve mutations affecting the ROCK pathway. A number of proteins that participate in the actin-myosin contractility are encoded by genes on commonly amplified chromosomes in hESCs. However, although these two sublines have some overt karyotypic abnormalities in common, notably gains of the long arm of chromosome 17, they almost certainly have other genetic and epigenetic changes that are not apparent at the gross G-banded karyotype level (Amps et al., 2011; Baker et al., 2007; Enver et al., 2005), so that their ability to overcome these bottlenecks cannot, at this stage, be ascribed to altered expression of genes specifically encoded in those shared regions.

Previous studies have indicated that the maintenance of undifferentiated hESCs is facilitated by culture at high-cell densities (Andrews et al., 1982; Thomson et al., 1998), and we have suggested that this depends upon NOTCH signaling requiring cell:cell contact (Fox et al., 2008). Our present time-lapse data do indeed confirm that the proliferation of normal hESCs is promoted by cell:cell contact. However, these data also reveal that the relationship between hESC proliferation and cell density involves a complex interplay with cell motility, which can be influenced by diffusible factors. Cells can acquire contact either through dividing and forming a colony with a sister cell or by migrating to form a colony with a nonsister cell. At low-plating density, which was prohibitive of cells meeting through migration, cells relied on mitosis for creating contacts. Nevertheless, the rare normal hESCs that did survive and proliferate under these conditions also belonged to a privileged clonogenic state that was characterized by superdiffusive motility; the Brownian-like cells did not seem to contribute to colonies. In the adapted cells, clonogenic capacity was also associated with motility, although in this case, both superdiffusive and Brownian-like cells formed colonies. The absence of nonmotile cells from the subsets of colony-forming hESCs might reflect cytoskeletal damage that interferes with both long-term proliferation of cells and motility. The future work should address the molecular underpinning of superdiffusive behavior and its association to survival and proliferation of hESCs, but this is likely to involve proteins that mediate actin cytoskeleton dynamics and/or proteins that regulate the crosstalk between cell motility and survival. ROCK is an example of one such protein, and certainly, inhibition of ROCK in the normal cells at low density enhanced motility as well as clonogenicity. The prosurvival phosphatidylinositol 3-kinase/Akt pathway has also been linked to motility in other cell types (Xue and Hemmings, 2013). Given that clonogenic/superdiffusive cells are very rare in normal hESC populations, identification of candidate genes will require either enriching the proportion of superdiffusive cells by modifying culture conditions or developing reliable techniques to isolate superdiffusive cells before subjecting them to gene expression analysis to identify the subset of genes that are differentially expressed in this particular fraction compared to Brownian or nonmotile cells.

Similar to previously reported effects of plating cell density on cell motility (Li et al., 2010), we noted that at high-plating density, cells exhibited enhanced motility, thus allowing nonsister cell contacts through migration. Under these high-density conditions, the normal hESCs that gave rise to colonies came not only from superdiffusive cells but also from the Brownian-like cells, thus now mimicking the behavior of adapted cells. Hence, the observed nonlinear rise in the growth of normal cells at increasing plating densities can be explained by an increased chance for cell:cell contact resulting from both a reduced average distance between cells and by increasing the motility of cells.

In contrast to the normal cells, the requirement for cell: cell contacts to promote survival was removed in the H7 and H14 adapted cells, implying the loss of niche dependence, as has also been reported elsewhere (Werbowetski-Ogilvie et al., 2009). Consequently, the functional significance of the observed increased motility of adapted cells remains unclear. One possibility is that increased motility plays a role in the early stages of adaptation, allowing cells to make more contacts, but becomes superfluous once the adapted cells acquire further changes that render them cell contact independent. If this is the case, monitoring motility could provide a noninvasive approach for the early detection of culture adaptation.

Time-lapse analysis reveals the marked complexity of hESC cultures and the multifaceted interplay of the cell interactions that drive the behavior of these cells and, ultimately, their fate choices. Our study has demonstrated not only the importance of signals mediated by cell:cell contact in promoting hESC proliferation but also the heterogeneity of these cells with respect to their clonogenic and motility patterns, which are influenced by diffusible signaling molecules. Furthermore, that all three bottlenecks to survival of hESCs during passaging were overcome by the ROCK inhibitor as well as adaptation in both H7 and H14 hESCs suggests a common pathway to overcoming these bottlenecks involving signaling associated with the cytoskeleton, which could also influence cell motility. Understanding

the molecular basis for these different signals and how they interact will be essential for designing culture conditions that minimize the selective advantages of particular mutations and so reduce the occurrence of variant cells during prolonged culture and scale-up.

EXPERIMENTAL PROCEDURES

Cell Culture

hESC lines used in this study were H7 and H14 (Thomson et al., 1998). Karyotypically normal sublines of H7 and H14 are termed H7.s14 and H14.s9, respectively. Karyotypically abnormal sublines of H7 (termed H7.s6) (47,XX,+1,der(6)t(6;17) (q27;q1)) and H14 (termed H14.BJ1) (48,XY,+12,+der(17) (p12p13.3) hsr(17) (p11.2)) have arisen spontaneously in culture (Baker et al., 2007; Draper et al., 2004; Enver et al., 2005).

Time-Lapse Video Microscopy

Time-lapse microscopy was performed by taking one frame every 10 min over 72 hr, using an Olympus Ix70 microscope controlled by Simple PCI software (Compix). During imaging, cells were enclosed in a chamber maintained at 37°C under a humidified atmosphere of 5% CO_2 in air.

Motility Analysis

Cell motility was analyzed using fractional diffusion techniques from Dieterich et al. (2008). The Ornstein-Uhlenbeck model was used to characterize Brownian-like motion, and the fractional Klein-Kramers (FKK) was used to characterize superdiffusive motion.

Detailed experimental methods and a description of statistical analysis, lineage tree reconstruction, segmentation, and tracking are provided in Supplemental Experimental Procedures.

SUPPLEMENTAL INFORMATION

Supplemental Information includes Supplemental Experimental Procedures, five figures, two tables, and seven movies and can be found with this article online at http://dx.doi.org/10.1016/j.stemcr.2014.05.006.

AUTHOR CONTRIBUTIONS

I.B., V.B., D.C., and P.W.A. conceived and designed the experiments. I.B., V.B., P.J.G., M.J., D.S., and A.G. performed the experiments. V.B. and D.C. developed numerical algorithms. I.B. and V.B. analyzed the data. I.B., V.B., P.J.G., D.C., and P.W.A. wrote the paper.

ACKNOWLEDGMENTS

We thank Mr. David Preskey for help with cell cultures and fluorescence-activated cell sorting and Dr. Andrew Hills for the MATLAB to mp4 conversion tool. This work was supported by the Medical Research Council, Human Frontier Science Program, and European Research Council Advanced Investigator Grant. I.B. was also supported by Wellcome Trust discipline hopping fellowship and D.C. by Medical Research Council discipline hopping fellowship.

Received: January 8, 2014
Revised: May 7, 2014
Accepted: May 8, 2014
Published: June 12, 2014

REFERENCES

Amps, K., Andrews, P.W., Anyfantis, G., Armstrong, L., Avery, S., Baharvand, H., Baker, J., Baker, D., Munoz, M.B., Beil, S., et al.; International Stem Cell Initiative (2011). Screening ethnically diverse human embryonic stem cells identifies a chromosome 20 minimal amplicon conferring growth advantage. Nat. Biotechnol. *29*, 1132–1144.

Andrews, P.W., Goodfellow, P.N., Shevinsky, L.H., Bronson, D.L., and Knowles, B.B. (1982). Cell-surface antigens of a clonal human embryonal carcinoma cell line: morphological and antigenic differentiation in culture. Int. J. Cancer *29*, 523–531.

Avery, S., Hirst, A.J., Baker, D., Lim, C.Y., Alagaratnam, S., Skotheim, R.I., Lothe, R.A., Pera, M.F., Colman, A., Robson, P., et al. (2013). BCL-XL mediates the strong selective advantage of a 20q11.21 amplification commonly found in human embryonic stem cell cultures. Stem Cell Rep. *1*, 379–386.

Baker, D.E., Harrison, N.J., Maltby, E., Smith, K., Moore, H.D., Shaw, P.J., Heath, P.R., Holden, H., and Andrews, P.W. (2007). Adaptation to culture of human embryonic stem cells and oncogenesis in vivo. Nat. Biotechnol. *25*, 207–215.

Barbaric, I., Gokhale, P.J., Jones, M., Glen, A., Baker, D., and Andrews, P.W. (2010). Novel regulators of stem cell fates identified by a multivariate phenotype screen of small compounds on human embryonic stem cell colonies. Stem Cell Res. (Amst.) *5*, 104–119.

Becker, K.A., Ghule, P.N., Therrien, J.A., Lian, J.B., Stein, J.L., van Wijnen, A.J., and Stein, G.S. (2006). Self-renewal of human embryonic stem cells is supported by a shortened G1 cell cycle phase. J. Cell. Physiol. *209*, 883–893.

Campa, V.M., Gutiérrez-Lanza, R., Cerignoli, F., Díaz-Trelles, R., Nelson, B., Tsuji, T., Barcova, M., Jiang, W., and Mercola, M. (2008). Notch activates cell cycle reentry and progression in quiescent cardiomyocytes. J. Cell Biol. *183*, 129–141.

Chen, G., Hou, Z., Gulbranson, D.R., and Thomson, J.A. (2010). Actin-myosin contractility is responsible for the reduced viability of dissociated human embryonic stem cells. Cell Stem Cell *7*, 240–248.

Dieterich, P., Klages, R., Preuss, R., and Schwab, A. (2008). Anomalous dynamics of cell migration. Proc. Natl. Acad. Sci. USA *105*, 459–463.

Draper, J.S., Pigott, C., Thomson, J.A., and Andrews, P.W. (2002). Surface antigens of human embryonic stem cells: changes upon differentiation in culture. J. Anat. *200*, 249–258.

Draper, J.S., Smith, K., Gokhale, P., Moore, H.D., Maltby, E., Johnson, J., Meisner, L., Zwaka, T.P., Thomson, J.A., and Andrews, P.W. (2004). Recurrent gain of chromosomes 17q and 12 in cultured human embryonic stem cells. Nat. Biotechnol. *22*, 53–54.

Enver, T., Soneji, S., Joshi, C., Brown, J., Iborra, F., Orntoft, T., Thykjaer, T., Maltby, E., Smith, K., Abu Dawud, R., et al. (2005). Cellular

differentiation hierarchies in normal and culture-adapted human embryonic stem cells. Hum. Mol. Genet. *14*, 3129–3140.

Fox, V., Gokhale, P.J., Walsh, J.R., Matin, M., Jones, M., and Andrews, P.W. (2008). Cell-cell signaling through NOTCH regulates human embryonic stem cell proliferation. Stem Cells *26*, 715–723.

Harrison, N.J., Baker, D., and Andrews, P.W. (2007). Culture adaptation of embryonic stem cells echoes germ cell malignancy. Int. J. Androl. *30*, 275–281, discussion 281.

Joshi, I., Minter, L.M., Telfer, J., Demarest, R.M., Capobianco, A.J., Aster, J.C., Sicinski, P., Fauq, A., Golde, T.E., and Osborne, B.A. (2009). Notch signaling mediates G1/S cell-cycle progression in T cells via cyclin D3 and its dependent kinases. Blood *113*, 1689–1698.

Laurent, L.C., Ulitsky, I., Slavin, I., Tran, H., Schork, A., Morey, R., Lynch, C., Harness, J.V., Lee, S., Barrero, M.J., et al. (2011). Dynamic changes in the copy number of pluripotency and cell proliferation genes in human ESCs and iPSCs during reprogramming and time in culture. Cell Stem Cell *8*, 106–118.

Li, L., Wang, B.H., Wang, S., Moalim-Nour, L., Mohib, K., Lohnes, D., and Wang, L. (2010). Individual cell movement, asymmetric colony expansion, rho-associated kinase, and E-cadherin impact the clonogenicity of human embryonic stem cells. Biophys. J. *98*, 2442–2451.

Metzler, R., and Klafter, J. (2000). The random walk's guide to anomalous diffusion: a fractional dynamics approach. Phys. Rep. *339*, 1–77.

Ohgushi, M., Matsumura, M., Eiraku, M., Murakami, K., Aramaki, T., Nishiyama, A., Muguruma, K., Nakano, T., Suga, H., Ueno, M., et al. (2010). Molecular pathway and cell state responsible for dissociation-induced apoptosis in human pluripotent stem cells. Cell Stem Cell *7*, 225–239.

Olariu, V., Harrison, N.J., Coca, D., Gokhale, P.J., Baker, D., Billings, S., Kadirkamanathan, V., and Andrews, P.W. (2010). Modeling the evolution of culture-adapted human embryonic stem cells. Stem Cell Res. (Amst.) *4*, 50–56.

Rand, M.D., Grimm, L.M., Artavanis-Tsakonas, S., Patriub, V., Blacklow, S.C., Sklar, J., and Aster, J.C. (2000). Calcium depletion dissociates and activates heterodimeric notch receptors. Mol. Cell. Biol. *20*, 1825–1835.

Thomson, J.A., Itskovitz-Eldor, J., Shapiro, S.S., Waknitz, M.A., Swiergiel, J.J., Marshall, V.S., and Jones, J.M. (1998). Embryonic stem cell lines derived from human blastocysts. Science *282*, 1145–1147.

Walker, A., Su, H., Conti, M.A., Harb, N., Adelstein, R.S., and Sato, N. (2010). Non-muscle myosin II regulates survival threshold of pluripotent stem cells. Nat. Commun. *1*, 71.

Watanabe, K., Ueno, M., Kamiya, D., Nishiyama, A., Matsumura, M., Wataya, T., Takahashi, J.B., Nishikawa, S., Nishikawa, S., Muguruma, K., and Sasai, Y. (2007). A ROCK inhibitor permits survival of dissociated human embryonic stem cells. Nat. Biotechnol. *25*, 681–686.

Werbowetski-Ogilvie, T.E., Bossé, M., Stewart, M., Schnerch, A., Ramos-Mejia, V., Rouleau, A., Wynder, T., Smith, M.J., Dingwall, S., Carter, T., et al. (2009). Characterization of human embryonic stem cells with features of neoplastic progression. Nat. Biotechnol. *27*, 91–97.

Xue, G., and Hemmings, B.A. (2013). PKB/Akt-dependent regulation of cell motility. J. Natl. Cancer Inst. *105*, 393–404.

Yugawa, T., Nishino, K., Ohno, S., Nakahara, T., Fujita, M., Goshima, N., Umezawa, A., and Kiyono, T. (2013). Noncanonical NOTCH signaling limits self-renewal of human epithelial and induced pluripotent stem cells through ROCK activation. Mol. Cell. Biol. *33*, 4434–4447.

Stem Cell Reports, Volume *3*

Supplemental Information

Time-Lapse Analysis of Human Embryonic Stem Cells Reveals Multiple Bottlenecks Restricting Colony Formation and Their Relief upon Culture Adaptation

Ivana Barbaric, Veronica Biga, Paul J. Gokhale, Mark Jones, Dylan Stavish, Adam Glen, Daniel Coca, and Peter W. Andrews

Figure S1.

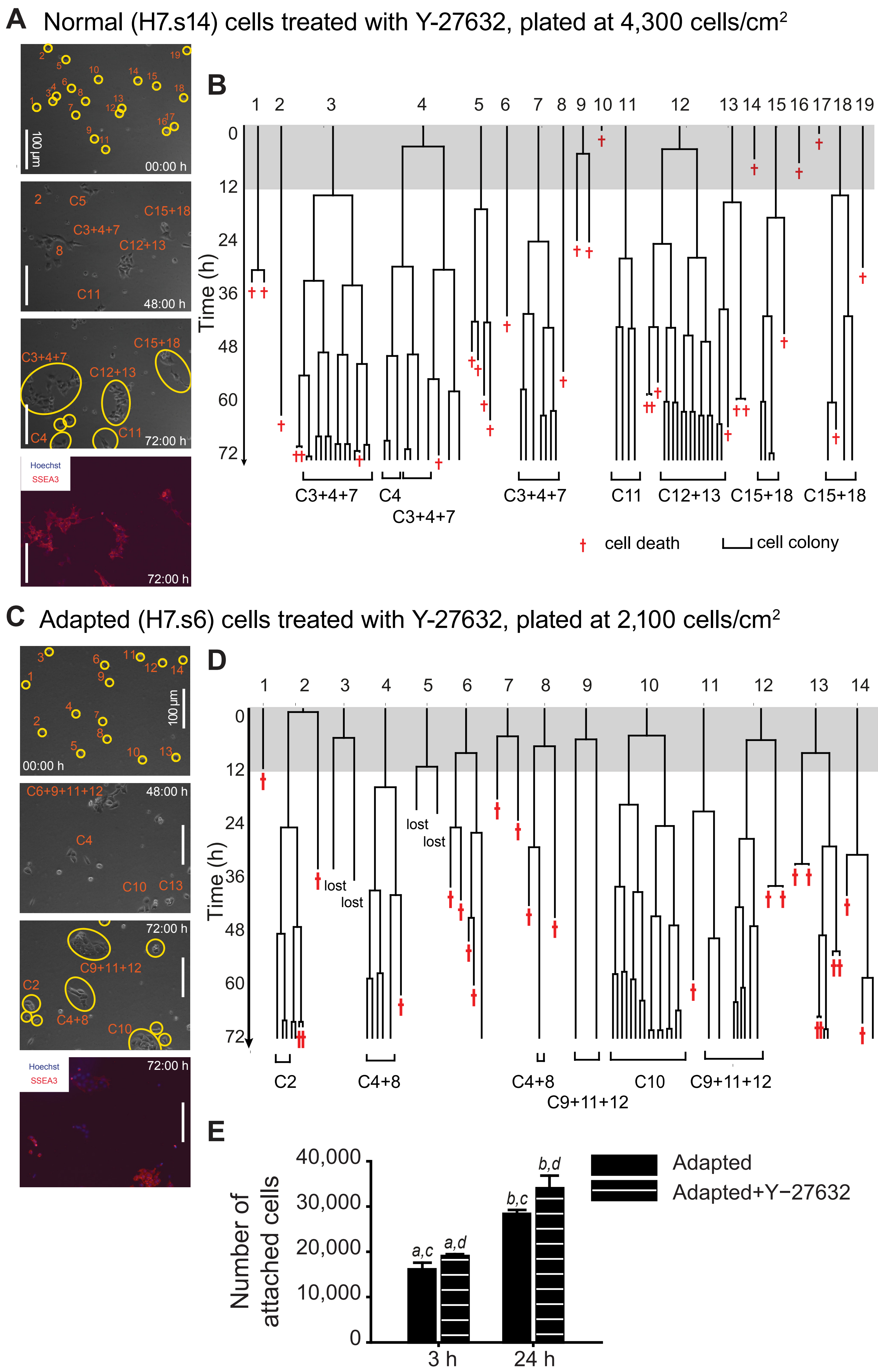
A
Normal (H7.s14) cells treated with Y-27632, plated at 4,300 cells/cm2
100 µm
00:00 h
48:00 h
72:00 h
Hoechst
SSEA3
72:00 h
B
Time (h)
C3+4+7
C4
C3+4+7
C3+4+7
C11
C12+13
C15+18
C15+18
cell death
cell colony
C
Adapted (H7.s6) cells treated with Y-27632, plated at 2,100 cells/cm2
100 µm
00:00 h
48:00 h
72:00 h
Hoechst
SSEA3
72:00 h
D
Time (h)
lost
C2
C4+8
C4+8
C9+11+12
C10
C9+11+12
E
Number of attached cells
40,000
30,000
20,000
10,000
0
3 h
24 h
a,c
a,d
b,c
b,d
Adapted
Adapted+Y−27632

Figure S2.

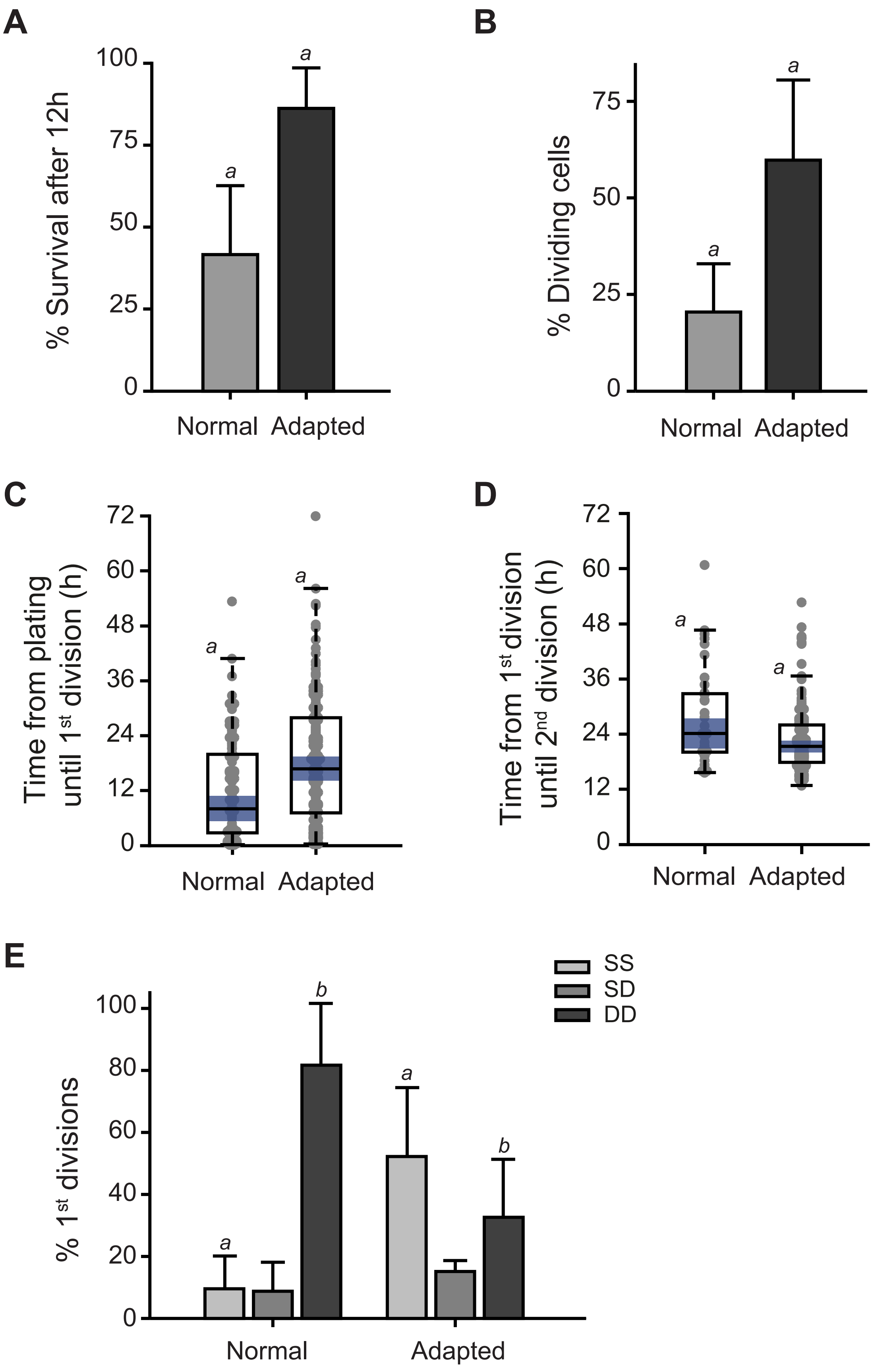
A
% Survival after 12h
100
75
50
25
0
a
a
Normal
Adapted
B
% Dividing cells
75
50
25
0
a
a
Normal
Adapted
C
Time from plating until 1st division (h)
72
60
48
36
24
12
0
a
a
Normal
Adapted
D
Time from 1st division until 2nd division (h)
72
60
48
36
24
12
0
a
a
Normal
Adapted
E
% 1st divisions
100
80
60
40
20
0
SS
SD
DD
a
b
a
b
Normal
Adapted

Figure S3.

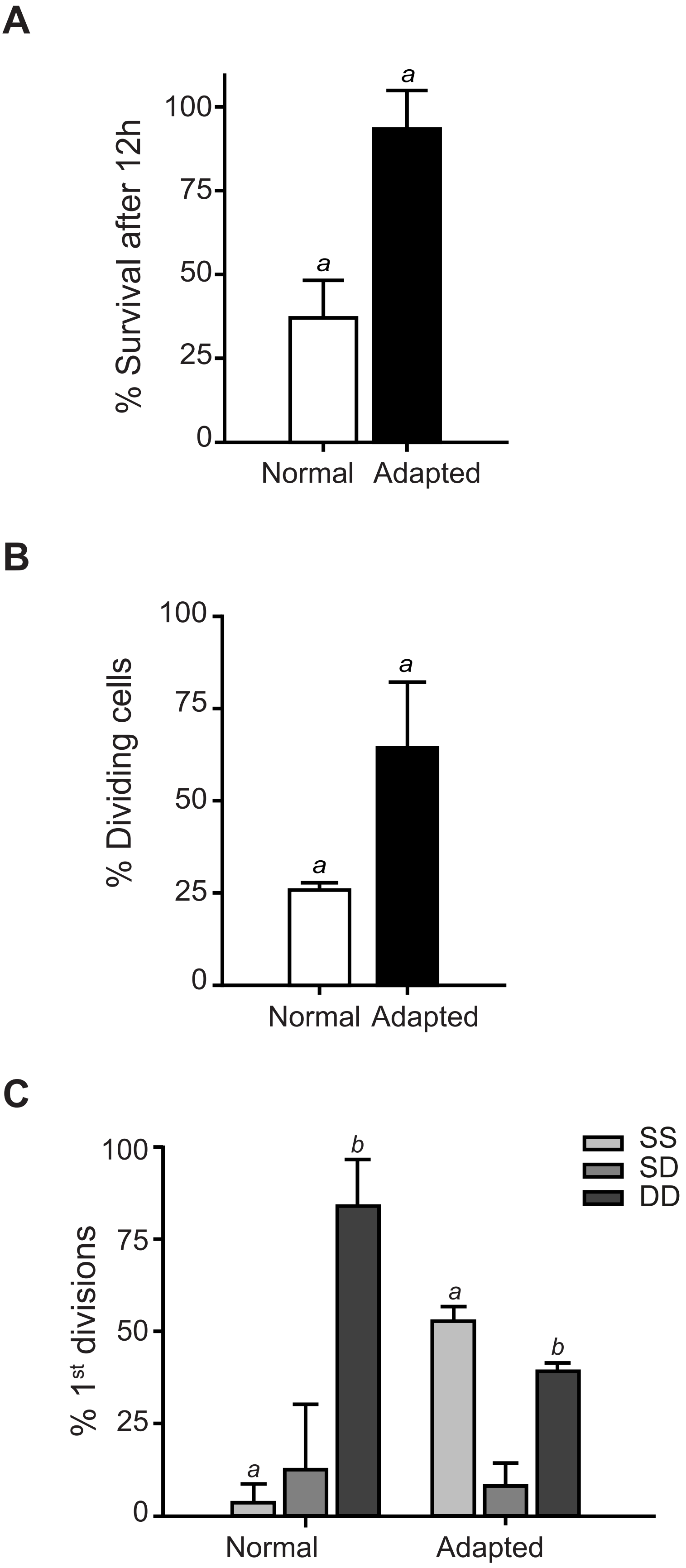
A
% Survival after 12h
0
25
50
75
100
a
a
Normal
Adapted
B
% Dividing cells
0
25
50
75
100
a
a
Normal
Adapted
C
% 1st divisions
0
25
50
75
100
SS
SD
DD
a
b
a
b
Normal
Adapted

Figure S4.

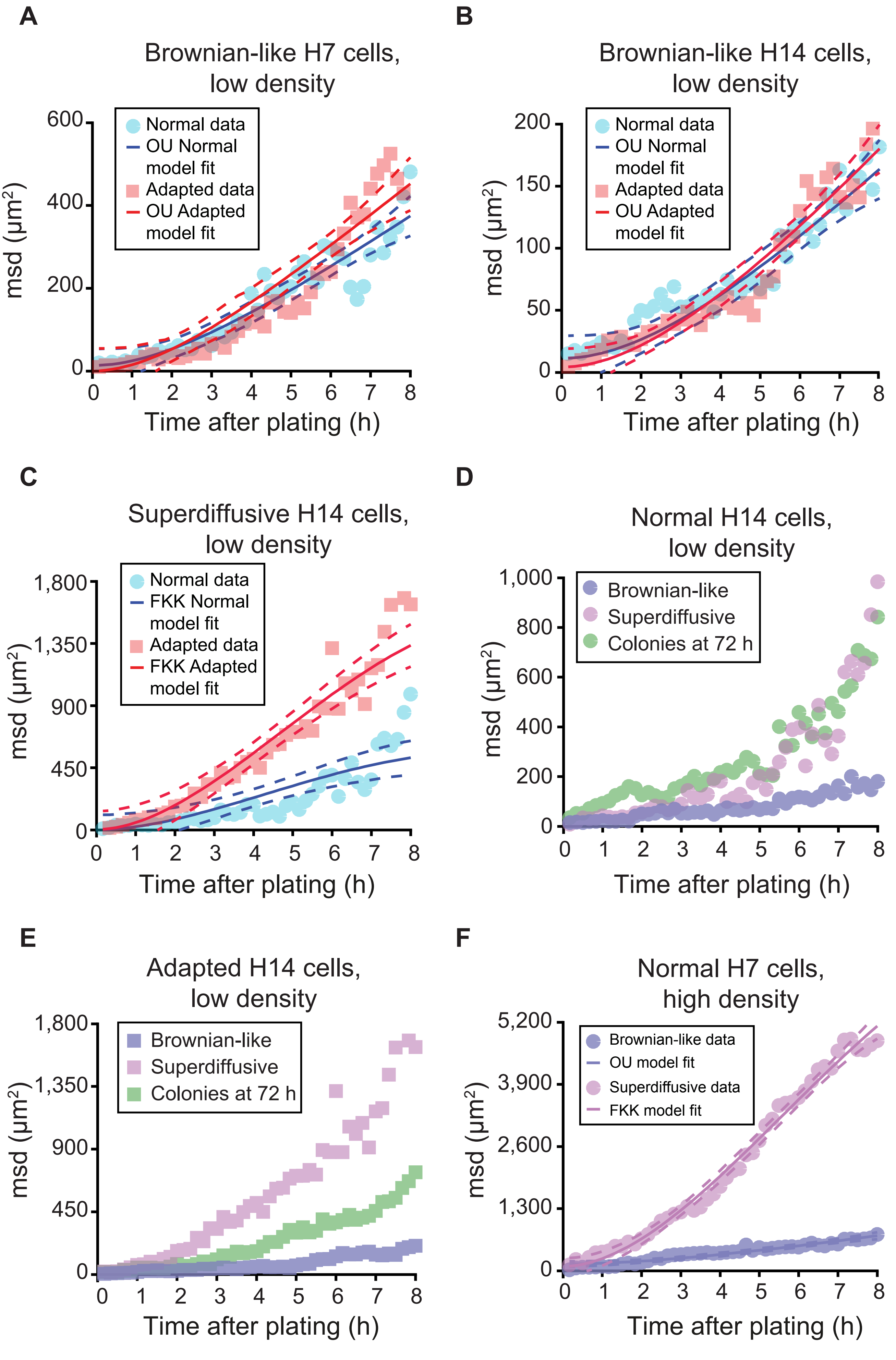
A
Brownian-like H7 cells, low density
Normal data
OU Normal model fit
Adapted data
OU Adapted model fit
msd (μm²)
Time after plating (h)
B
Brownian-like H14 cells, low density
Normal data
OU Normal model fit
Adapted data
OU Adapted model fit
msd (μm²)
Time after plating (h)
C
Superdiffusive H14 cells, low density
Normal data
FKK Normal model fit
Adapted data
FKK Adapted model fit
msd (μm²)
Time after plating (h)
D
Normal H14 cells, low density
Brownian-like
Superdiffusive
Colonies at 72 h
msd (μm²)
Time after plating (h)
E
Adapted H14 cells, low density
Brownian-like
Superdiffusive
Colonies at 72 h
msd (μm²)
Time after plating (h)
F
Normal H7 cells, high density
Brownian-like data
OU model fit
Superdiffusive data
FKK model fit
msd (μm²)
Time after plating (h)

Figure S5.

A

Normal (H7.s14) cells plated at low density (2,100 cells/cm^2)

B

Normal (H7.s14) cells plated at high density (10,600 cells/cm^2)

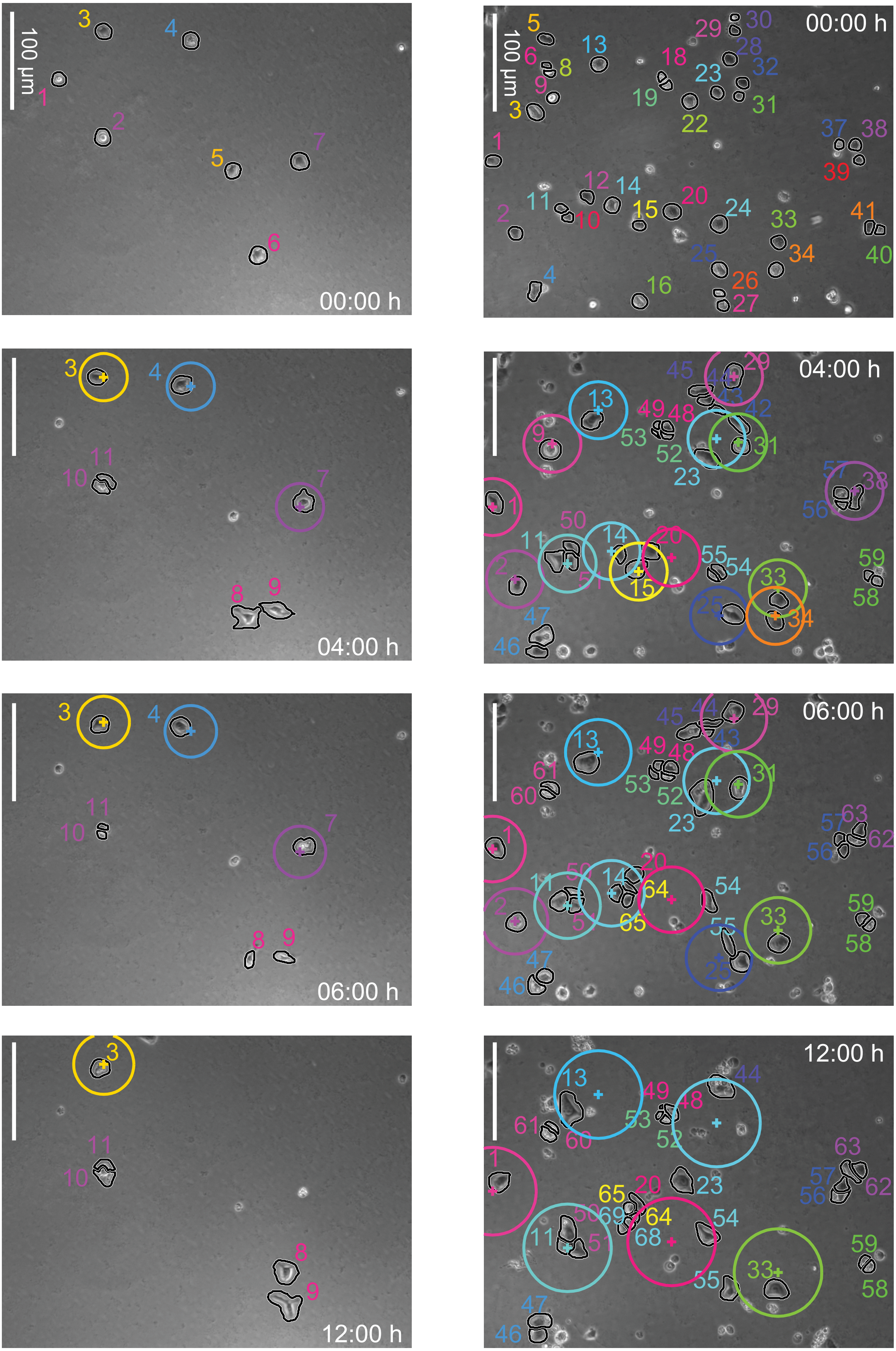

Supplemental Figure Legends

Figure S1. Single-cell behaviour of normal H7.s14 and adapted H7.s6 cells treated with the ROCK inhibitor Y-27632 investigated by time-lapse analysis.

(**A**) Time-lapse analysis of SSEA3-sorted normal cells treated with Y-27632 plated at low density. Still images are taken from **Movie S3** (available online). Cells labelled 1 to 19 are circled in the upper-most panel. Colonies present at 72h were partly from single cells (colonies labelled C4 and C11 originating from cells 4 and 11 respectively) and partly mixed (colonies labelled C3+4+7, C12+13 and C15+18 originating from cells with corresponding labels) and there were rare satellite colonies formed (cell 4 gives rise to progeny in two colonies labelled C4 and C3+4+7, with the addition of two single cells). The resulting colonies were stained for SSEA3 (red) and nuclei counterstained with Hoechst 33342 (blue) at the end of the filming (lower-most panel).

(**B**) Lineage trees of SSEA3-sorted normal cells treated with Y-27632 constructed from the **Movie S3**. Numbers correspond to cells circled in (**A**, upper-most panel). Colonies present at 72h (clonal colonies are labelled C4 and C11 and non-clonal colonies are labelled C3+4+7, C12+13 and C15+18 according to their constituent originating cells) are also circled in (**A**, the second panel from the bottom). Red crosses denote cell death. Gray shaded area indicates first 12h postplating.

(**C**) Time-lapse analysis of SSEA3-sorted adapted cells treated with Y-27632 plated at low density. Still images are taken from **Movie S4**. Cells labelled 1 to 14 are circled in the upper-most panel. Colonies present at 72h were partly from single cells (colonies labelled C2 and C10 originating from cells 2 and 10 respectively) and partly mixed (for example colonies labelled C4+8 and C9+11+12 originating from cells with corresponding

labels) and we noted single cells leaving colonies (progeny of cell 2 and cell 13 leave clonal colonies and surviving progeny of cell 6 leaves non-clonal colony C9+11+12). The resulting colonies were stained for SSEA3 (red) and nuclei counterstained with Hoechst 33342 (blue) at the end of the filming (lower-most panel).

(**D**) Lineage trees of SSEA3-sorted adapted cells treated with Y-27632 constructed from **Movie S4**. Numbers correspond to cells circled in (**C**, upper-most panel). Colonies present at 72h (clonal colonies labelled C2 and C10 and non-clonal colonies labelled C4+8 and C9+11+12 according to their constituent originating cells) are also circled in (**C**, the second panel from the bottom). Incomplete lineage trees are labelled as 'lost' due to cells escaping from the field of view. Red crosses denote cell death. Gray shaded area indicates the first 12h postplating.

(**E**) Representative numbers of attached adapted (H7.s6) cells and adapted cells treated with Y-27632 analysed after 3h and 24h postplating. Results are means of triplicate wells from the same experiment ± SD (a-d represent Student's t tests, left tail; p values: a<0.05; b<0.05; c<0.001; d=0.001).

Figure S2. Bottlenecks limiting clonal expansion confirmed on another pair of normal cells H14.s9 and their karyotypically adapted counterparts H14.BJ1 analysed at low plating density.

(**A**) Percentage of cells from the initial plated population that survived the first 12h after plating for normal (H14.s9) cells and adapted (H14.BJ1) cells. Results are means of triplicate independent experiments ± SD (a represents Student's t test, left tail; p value<0.01). Data consist of 150 to 340 cells per culture.

(**B**) Percentage of dividing cells in the normal and adapted cultures. Results are means of triplicate independent experiments ± SD (a represents Student's t test, left tail; p value<0.05). Data consist of 150 to 340 cells per culture.

(**C**) The cell cycle re-entry time following plating for normal and adapted cells. Results are box plot representations of distributions from triplicate independent experiments with median line and 95% median confidence intervals (blue); observations (gray dots) shown stretching horizontal by frequency (a represents Kruskal-Wallis test, p value$<10^{-5}$). Data consist of 75 to 165 cells per culture.

(**D**) Cell cycle time in subsequent divisions in normal and adapted cells. Results are box plot representations of distributions from triplicate independent experiments with median line and 95% confidence intervals (blue); observations (gray dots) shown stretching horizontal by frequency (a represents Kruskal-Wallis test, p value<0.05). Data consist of 30 to 120 cells per culture.

(**E**) Percentage of first divisions following which both daughter cells divide further (SS: survival-survival), one daughter cell divides while the other dies (SD: survival-death) or both daughter cells die (DD: death-death) in normal and adapted cultures. Results are means of triplicate independent experiments ± SD (a,b represent Student's t tests, a: left tail, p value<0.05, b: right tail, p value<0.05). Data consist of 60 to 65 cells per culture.

Figure S3. Survival of normal cells H7.s14 and their karyotypically adapted counterparts H7.s6 serially passaged on Matrigel then plated at low plating density and analysed using time-lapse.

(**A**) Percentage of cells from the initial plated population that survived the first 12h after plating for normal (H7.s14) cells and adapted (H7.s6) cells serially passaged on Matrigel. Results are means of triplicate wells from the same experiment ± SD (a represents Student's t test, left tail; p value<0.01). Data consist of 50 to 55 cells per culture.

(**B**) Percentage of dividing cells in the normal and adapted cells serially passaged on Matrigel. Results are means of triplicate wells from the same experiment ± SD (a represents Student's t test, left tail; p value=0.01). Data consist of 50 to 55 cells per culture.

(**C**) Percentage of first divisions following which both daughter cells divide further (SS: survival-survival), one daughter cell divides while the other dies (SD: survival-death) or both daughter cells die (DD: death-death) in normal and adapted cells serially passaged on Matrigel. Results are means of duplicate independent experiments ± SD (a, b represent Student's t tests, a: left tail; p value<0.01, b: right tail; p value<0.05). Data consist of 20 to 30 cell divisions per culture.

Figure S4. Motility of normal and adapted H7 and H14 hESC sublines characterised using fractional diffusion models.

(**A**) Mean squared displacement (msd) calculated from trajectories of Brownian-like normal (H7.s14) and adapted (H7.s6) cells plated at low density showing similar movement characteristics. Data collected in both normal and adapted H7 sublines consisted of cells that were classified according to msd into a Brownian-like and a superdiffusive fraction, with the addition of non motile cells. Only motile cells were analysed, with the msd for super-diffusive cells included in **Figure 7A**. Markers represent

msd from real data, solid lines show predictions of the Ornstein-Uhlenbeck (OU) model which corresponds to Brownian-like motion and dashed lines indicate 95% model prediction bounds. Model parameters are specified in **Table S1**. Data consist of 14 to 21 cells per culture.

(**B**) msd calculated from trajectories of Brownian-like normal (H14.s9) and adapted (H14.BJ1) cells plated at low density showing similar movement characteristics. Data collected in both H14 sublines consisted of cells that were classified according to msd into Brownian-like and superdiffusive, with the addition of non motile cells. Markers represent ensemble msd from real data, solid lines show predictions of the Ornstein-Uhlenbeck (OU) model which corresponds to Brownian-like motion and dashed lines indicate 95% model prediction bounds. Model parameters are specified in **Table S2**. Data consist of 16 to 19 cells per culture.

(**C**) msd calculated from trajectories of superdiffusive normal (H14.s9) and adapted (H14.BJ1) cells plated at low density showing that superdiffusive adapted cells can travel further from origin compared to superdiffusive normal hESCs. Markers represent ensemble msd from real data, solid lines show the predictions of fractional Klein-Kramers (FKK) model, which corresponds to superdiffusive motion and dashed lines indicate 95% prediction bounds. Model parameters are specified in **Table S2**. Data consist of 10 cells per culture.

(**D**) msd for Brownian-like (also included in **B**) and superdiffusive (also included in **C**) normal (H14.s9) cells plated at low density with the rare 8 cells that give rise to colonies present at 72h showing similar movement characteristic as the superdiffusive normal hESCs. Markers represent ensemble msd from real data.

(**E**) msd for Brownian-like (also included in **B**) and superdiffusive (also included in **C**) adapted (H14.BJ1) cells plated at low density, with the cells that give rise to colonies present at 72h showing a mixture of Brownian-like and superdiffusive movement characteristics. Markers represent ensemble msd from real data. Data consist of 23 cells forming colonies.

(**F**) msd calculated from trajectories of Brownian-like and superdiffusive normal (H7.s14) hESCs plated at high density. Markers represent ensemble msd from real data, solid lines show predictions of the Ornstein-Uhlenbeck (OU) and the fractional Klein-Kramers (FKK) models which correspond to Brownian-like and superdiffusive motion respectively; dashed lines indicate 95% model prediction bounds. Model parameters are specified in **Table S1**. Data consist of 18 to 25 cells per category.

Figure S5. Early colony formation in normal H7.s14 cells at different cell plating densities observed in time-lapse microscopy.

(A) Tracking of single cells from time-lapse images of normal (H7.s14) hESCs plated at low density observed over 12h postplating showing that in this culture condition colonies are formed from single cells. Tracked cell boundaries are indicated with black contours and time after plating is specified in each image. Cells at the time of plating (upper-most panel) are labelled 1 to 7 and colour-coded to indicate distinct lineages. Cells 1, 5 and 7 die off during the indicated time period. Cells 2 and 6 divide early and give rise to daughter cells 10, 11 and 8, 9 respectively. Cell 4 is lost from the field of view after 12h (lower-most panel). Colour-coded crosses indicate the location of cells at plating time. With the exception of the upper-most panel, visualisations include colour coded circles

denoting model-predicted locations (cells are expected to remain inside the circled area) at the indicated times after plating. The radius of circles increases with time according to the spatial probability distribution function (pdf) of an equal weighted mixture of Brownian-like and superdiffusive cells. The radius is given by: (i) the distance that 95% of cells are expected to travel up to (for example, we estimated that 95% of cells plated at low density could travel up to 43μm after 12h (see **Figure 7E**) and (ii) added average cell radius (15μm). As a result of reduced motility, colonies present after 12h are clonal as indicated by a unique colour of cells in contact.

(**B**) Tracking of single cells from time-lapse images of normal (H7.s14) hESCs plated at high density taken from **Movie S7** showing that in this culture condition, colonies form from single cells and in addition, there are many non-clonal colonies after 12h postplating. Tracked cell boundaries are indicated with black contours and time after plating is specified in each image. Cells at the time of plating (upper-most panel) are labelled 1 to 41 and colour-coded to indicate distinct lineages. A number of cells are in contact from the time of plating (for example cells 10 and 11). Colonies form through a sequence of death, divisions and migration events. For example, cell 10 dies off; cell 12 divides into daughter cell 51 and daughter cell 50 which subsequently join with cell 11 to form a colony. Cells 14, 15 and 20 start off as single cells but migrate towards each other; cell 15 gives rise to daughter cells 64 and 65, while cell 14 gives rise to daughter cells 68 and 69 thus forming another mixed colony. Colour coded crosses indicate the location of cells at plating time. With the exception of the upper-most panel, visualisations include colour coded model-predicted locations (cells are expected to remain inside the circled area) at the indicated times after plating. The radius of circles increases with time

according to the spatial probability distribution function (pdf) of an equal weighted mixture of Brownian-like and superdiffusive cells. The radius is given by: (i) the distance that 95% of cells are expected to travel up to (for example, at high plating density, we estimated that 95% of cells could travel up to 76µm over 12h postplating time – see **Figure 7E**) and (ii) added average cell radius (15µm). As expected, most cells remain within the predicted area (cells 1, 11, 33 and 13 in the lower-most panel) while few migrate beyond this boundary however remaining in the proximity of the circle (cells 20 and 23 in the lower-most panel). The combined effect of reduced cell:cell distance and enhanced motility at the high density plating condition leads to many colonies present after 12h being non-clonal, as indicated by the multiple colours of cells in contact.

Movie S1. Time-lapse video of normal H7.s14 cells plated at low density and reconstructed lineage trees over 72h postplating. Initial plated cells labelled 1 to 18 correspond to those in **Figure 1A** (upper-most panel) and lineage trees are also included in **Figure 2A.**

Movie S2. Time-lapse video of adapted H7.s6 cells plated at low density and reconstructed lineage trees over 72h postplating. Initial plated cells labelled 1 to 12 correspond to those in **Figure 1B** (upper-most panel) and lineage trees are also included in **Figure 2B.**

Movie S3. Time-lapse video of normal H7.s14 cells treated with Y-27632, plated at low density and reconstructed lineage trees over 72h postplating. Initial plated cells

labelled 1 to 19 correspond to those in **Figure S1A** (upper-most panel) and corresponding lineage trees are also included in **Figure S1B**. Also see **Figure 2**.

Movie S4. Time-lapse video of adapted H7.s6 cells treated with Y-27632, plated at low density and reconstructed lineage trees over 72h postplating. Initial plated cells labelled 1 to 14 correspond to those in **Figure S1C** (upper-most panel) and corresponding lineage trees are also included in **Figure S1D**. Also see **Figure 2**.

Movie S5. Representative example of a three-cell clump of normal H7.s14 cells dissociated using non-enzymatic solution and observed in time-lapse over 72h postplating. Initial plated cells in the clump correspond to those in **Figure 5A** (upper-most panel) and lineage trees are included in **Figure 5B**.

Movie S6. Representative examples of cells tracked from time-lapse showing superdiffusive, Brownian-like and non-motile hESCs plated at low density. Related to **Figure 7** and **Figure S4**.

Movie S7. Single-cell tracking of time-lapse video showing normal H7.s14 cells plated at high density over 12h postplating. Cell labels correspond to those in **Figure S5B**. Also see **Figure 7**.

Supplemental Tables

Table S1. Diffusion model parameters estimated from normal H7.s14 and adapted H7.s6 hESCs at low plating density and normal H7.s14 cells plated at high density.

Population \ Parameter Name		Thermal speed v_{th} ($\mu m/min$)	Exponent $\alpha = 2 - \beta$	Damping coefficient γ ($1/min^{\alpha}$)	Standard deviation of noise η (μm)	Diffusion coefficient D_{α} ($\mu m^2/min^{2-\alpha}$)
Normal (H7.s14) Low density	Brownian-like	0.0421 $\pm$ 0.0262	1	0.0066 $\pm$0.0041	2.5866 $\pm$ 2.6632	0.2703 $\pm$ 0.0857
	Super-diffusive	0.0670 $\pm$ 0.0396	0.5506 $\pm$ 0.1014	0.0163 $\pm$ 0.0035	0.3244 $\pm$ 4.1178	0.2749 $\pm$ 0.0653
Adapted (H7.s6) Low density	Brownian-like	0.0493 $\pm$ 0.0323	1	0.0076 $\pm$ 0.0048	0.0001 $\pm$ 3.1066	0.3219 $\pm$ 0.1069
	Super-diffusive	0.0728 $\pm$ 0.0361	0.7000 $\pm$ 0.1161	0.0100 $\pm$ 0.0030	0.0001 $\pm$ 4.0661	0.5302 $\pm$ 0.1134
Normal (H7.s14) High density	Brownian-like	0.0493 $\pm$ 0.0279	1	0.0048 $\pm$ 0.0030	8.4462 $\pm$ 3.1387	0.5025 $\pm$ 0.1484
	Super-diffusive	0.1423 $\pm$ 0.0495	0.6004 $\pm$ 0.0413	0.0149 $\pm$ 0.0013	6.8083 $\pm$ 5.1945	1.3594 $\pm$ 0.1318

Table S2. Diffusion model parameters estimated from normal H14.s9 and adapted H14.BJ1 hESCs plated at low plating density.

Parameter Name / Population		Thermal speed	Exponent	Damping coefficient	Standard deviation of noise	Diffusion coefficient
		v_{th} ($\mu m/min$)	$\alpha = 2 - \beta$	γ ($1/min^{\alpha}$)	η (μm)	D_{α} ($\mu m^2/min^{2-\alpha}$)
Normal (H14.s9) Low density	Brownian-like	0.0253 ± 0.0161	1	0.0050 ± 0.0038	2.3734 ± 1.7974	0.1278 ± 0.0433
	Super-diffusive	0.0580 ± 0.0206	0.5319 ± 0.0045	0.0193 ± 0.0002	0.5384 ± 4.1474	0.1749 ± 0.0121
Adapted (H14.BJ1) Low density	Brownian-like	0.0272 ± 0.0146	1	0.0050 ± 0.0027	1.4599 ± 1.6232	0.1475 ± 0.0405
	Super-diffusive	0.0837 ± 0.0207	0.5643 ± 0.0027	0.0175 ± 0.0002	1.0964 ± 4.6012	0.4008 ± 0.0145

Supplemental Experimental Procedures

Human embryonic stem cell culture

Cells were maintained as previously described (Draper et al., 2002) at 37°C under a humidified atmosphere of 5% CO_2 in air, on mitotically-inactivated mouse embryonic fibroblasts (MEFs), in medium consisting of KnockOut DMEM (KO-DMEM) (Life Technologies Ltd, Paisley, UK) supplemented with 20% KnockOut Serum Replacement (Life Technologies), 1mM L-glutamine (Life Technologies), 0.1mM β-mercaptoethanol (Sigma-Aldrich, Poole, Dorset, UK), 1x non-essential amino acids (Life Technologies) and 4ng/ml basic FGF (Life Technologies) (Amit et al., 2000).

Compounds

Y-27632 was purchased from Sigma-Aldrich and used at 10μM in all the assays.

Antibodies

The following primary monoclonal antibodies were prepared in house as pre-titred supernatants from hybridoma MC631 (anti-SSEA3) (Shevinsky et al., 1982); antibody from the parental myeloma P3X63Ag8 (Kohler and Milstein, 1975) was used as a negative control. Secondary antibody used for SSEA3 in immunocytochemistry was Dylight-594-conjugated goat anti-rat IgM, μ -chain specific antibody (Jackson ImmunoResearch, Stratech, Newmarket, UK), and the secondary antibody used in cell sorting was fluorescein isothiocyanate (FITC)-conjugated goat anti-mouse IgG (H+L) antibody (Jackson ImmunoResearch).

Immunocytochemistry

Cells were fixed with 4% paraformaldehyde for 15 min at room temperature and blocked with 10% foetal calf serum in PBS (blocking buffer). They were then incubated with the anti-SSEA3 primary antibody in blocking buffer for 1h. After three washes with PBS, the cells were incubated with the Dylight-594-conjugated secondary antibody in the blocking buffer for 1h. This was followed by three washes with PBS. Nuclei were counter-stained with 10μg/ml Hoechst 33342 (Life Technologies).

Cell sorting

Cells were harvested using trypsin (0.25% trypsin with 0.2% EDTA in PBS) (Sigma-Aldrich) and resuspended in blocking buffer (PBS supplemented with 10% foetal calf serum). Cells were incubated for 30 min with a primary antibody to SSEA3. After washing three times in blocking buffer, cells were labelled with FITC-conjugated secondary antibody for 30 min. This was followed by washing the cells three times with blocking buffer and analyzing cell fluorescence in a MoFlo Cell Sorter (Dako). The gate for FITC-positive cells was set using control cells that were incubated with a negative control antibody obtained from the parent myeloma cell line P3X63Ag8 (Kohler and Milstein, 1975).

For cell sorting based on the cell cycle profile, after harvesting the cells with trypsin and staining for SSEA3 (as described above), cells were washed once in medium and then incubated with 10μg/ml Hoechst33342 (Life Technologies) in medium at 37°C for 30 min. Cells were then sorted using the MoFlo Cell Sorter (Dako).

Cloning efficiency

Cells were sorted for SSEA3 and cell cycle profile and 10,000 normal (H7.s14) or 5,000 adapted (H7.s6) cells were plated onto 6-well plates (Corning Costar, High Wycombe, UK) coated with Matrigel (BD Biosciences, Oxford, UK) (1:25 dilution in KO-DMEM) in mTESR medium (STEMCELL Technologies, Vancouver, Canada). Seven days after seeding the cells, colonies were fixed and stained for SSSEA3 as described above. Images of stained colonies were acquired using an automated microscopy platform (InCell Analyzer 1000, GE Healthcare, Little Chalfont, UK). Numbers of colonies and SSEA3-positive cells were counted from images using the Developer Toolbox 1.7 software (GE Healthcare), as previously described (Barbaric et al., 2010).

Time-lapse video microscopy

For single-cell analysis under the time-lapse, cells sorted for SSEA3 (and in some experiments cells sorted for SSEA3 and cell cycle profile) were plated onto 6-well plates (Corning Costar) coated in Matrigel (BD Biosciences) (1:25 dilution in KO-DMEM), in mTESR medium (STEMCELL Technologies). For analysis of cell behaviour at low plating density, cells were seeded at 2,100 or 4,300 cells/cm^2, whereas the high plating density used was 10,600 cells/cm^2. One frame was taken every 10 min over 72h using an Olympus Ix70 microscope controlled by Simple PCI software (Compix, Brandywine, PA). During imaging, cells were enclosed in a chamber maintained at 37°C under a humidified atmosphere of 5% CO_2 in air. At the end of the time-lapse, cells were fixed and stained for SSEA3. Images of stained cells were acquired using an automated microscopy platform (InCell Analyzer 1000, GE Healthcare). Images were analyzed

using Developer Toolbox 1.9 software (GE Healthcare) as previously described (Barbaric et al., 2010).

For the time-lapse microscopy of single cells after serial passaging on Matrigel, cells grown on MEFs were passaged onto Matrigel in mTESR and maintained in these conditions for three to four passages. SSEA3-sorted single cells were then plated onto 6-well plates coated in Matrigel in mTESR medium and filmed as described above.

For the time-lapse microscopy of cells passaged in small clumps, cells grown in colonies on MEFs were passaged using Cell Dissociation Solution (Biological Industries, Kibbutz Beit-Haemek, Israel) to obtain clumps of approximately three to four cells. Clumps were then plated onto 6-well plates coated in Matrigel in mTESR medium and filmed as described above.

Statistical analysis

Statistical analysis was produced using standard methods from the Statistics Toolbox of Matlab R2010b. Replicates were created from independent experiments or wells from the same experiment as indicated in figure legends. Bar plot analysis consist of population counts from multiple experiments as a ratio of the entire population and indicates mean values with whiskers denoting one standard deviation (SD) above the mean. Bar plot results were Gaussian-distributed and compared using the 2-sample Student's t test using left tail or right tail to ascertain whether one mean is lower or higher than the other respectively.

Box plot analysis indicates values between the 25th (q_1) and 75th (q_2) percentile as the bottom and top edges of the box; maximum whiskers length is $w = 2.7SD$; values lying

outside the whisker length on either side of the box (lower than $q_2 + w (q_2 – q_1)$ or higher than $q_1 – w (q_2 – q_1)$, respectively) are considered outliers; the level of the distribution is indicated by the median value and 95% median confidence intervals. Box plots are accompanied by a display of data using gray dots binned by value indicated on the y-axis and stretching horizontally by frequency. Distributions in the boxplot analysis were non-Gaussian and comparisons were made using the Kruskal-Wallis test. Tests were performed pairwise and we considered significant those differences with an associated p value < 0.05.

Lineage tree reconstruction

Lineage datasets were collected partly using an automated tracker and partly manually. Data was recorded in an electronic format, which allowed performing statistical analyses and visualisations. The lineage tree data structure is unique to each time-lapse field and contains information of the time of death or division and progeny (where applicable) for all recorded cells, the number of cells at plating time and the length of the time-lapse experiment. Cells are denoted in the data by a unique numeric label 1,2,.. and in cases where a division event occurs, unique numeric labels are created for the two daughter cells, such that the total number of cells recorded is continuously increasing. All numeric labels are stored into an organised data structure linking the parent cell to its progeny. Where the automated extractor output was used, it has been compared to the time-lapse video data to ensure that death and division events were correctly detected.

Segmentation and tracking of cell trajectories

We developed an automated segmentation and tracking model, which allows the detection of multiple non-overlapping objects with coupled geometric active contours. A detailed review of geometric active contour models may be found in (Cremers et al., 2007). Our algorithm relies on the partitioning of an image into two regions (target and background), with the target region further partitioned into multiple connected regions representing cell interiors (Biga et al., manuscript in preparation). Tracking was achieved by feeding the processing result of the current frame to be used as initialisation in processing the following one. Information regarding cell fate (death or division), death or division time, progeny (where applicable), as well as time series of cell location (representing un-weighted centre of mass coordinates of the region corresponding to each cell over time) and other cell statistics (morphology, relative cell:cell distance) was extracted in an automated fashion and stored in a data structure ready to be used in statistical analysis and visualisations.

Cell:cell contact detection and quantification from time-lapse images

We considered a colony to be a collection of a minimum of two cells, where any cell in the collection is found in direct cell:cell contact with at least one other cell in the same group. In automated data extraction, cell:cell contacts were identified using the geometric active contours representation of cell interior and cell boundary. The contours of any particular cell evolved from the initial cell representation at plating time $t = 0$ to subsequent representations at postplating times $t > 0$. The distance between any two

segmented cells was computed as the minimum Euclidean distance between any two pixels from contour pairs:

$$d_{ij}(t) = \min\left\{\sqrt{\left(x_i(t) - x_j(t)\right)^2 + \left(y_i(t) - y_j(t)\right)^2}, \forall j = \overline{1:N(t)}, j \neq i\right\} \tag{1}$$

where $(x_i(t), y_i(t))$ denote boundary pixels and $N(t)$ represents the total number of cells alive at time t. Any two cells are considered in direct cell:cell contact at time t if their contours satisfy the condition $d_{ij}(t) = 0$.

The proportion of cells that had come into contact with one or more other cells was calculated from multiple fields collected from independent experiments. It represents a cumulative count of all cells that had at least one contact during the observation period indicated on the x-axis, proportional to the total number of initial plated cells present in all analysed fields. Where more than one contact is indicated, only the ratio (number of cells that had n contacts proportional to the total number of cells) for the highest number of contacts (denoted by n) is considered. For example, if cell A had a single neighbour up to 2h postplating but then acquired two neighbours at 3h, then A is included in the bin count for $n = 1$ from 0h to <3h, however after 3h postplating A is included in the bin count $n = 2$.

Movement classification for single cell trajectories

Trajectories of single cells were extracted from video data as timeseries of coordinates $(x_i(t), y_i(t))$, denoting the center of mass (corresponding to active contours identifying each cell interior) in consecutive frames observed up to 12h postplating. We computed the time-averaged $msd_i, i = \overline{1:N(0)}$ (Kusumi et al., 1993),(Qian et al., 1991):

$$\mathrm{msd_i(k\delta t)} = \frac{1}{K-k-1}\sum_{j=1}^{K-k-1}\left[\left(x_i(j\delta t + k\delta t) - x_i(j\delta t)\right)^2 + \left(y_i(j\delta t + k\delta t) - y_i(j\delta t)\right)^2\right] \quad (2)$$

Where K is the lifespan of cell i and k represents the number of time lags δt.

The average cell radius was estimated to be the same for normal and adapted cells in a particular cell line: approximately 15μm in H7 and 11μm in H14, which is consistent with reports from literature (Li et al., 2010).

The $\mathrm{msd_i}$ of cells that showed superdiffusive movement transitioned from a stretched-exponential to a power law behaviour of type $\mathrm{msd} \sim t^{\beta}$ in the long time limit, $t \to \infty$ (Metzler and Klafter, 2000). Therefore we considered only the motile cells that had long timeseries of cell locations to show a clear transition (> 5h).

We adapted the method in (Kusumi et al., 1993) to take into account long time characteristics of movement. We characterised the $\mathrm{msd_i}$ shape through a linear fitting across the entire length of the curve. We then assessed the performance of the linear fitting to be sufficient if it explained a large proportion of the variance in the data ($\mathrm{rsquare} \geq 0.95$) and the root mean squared error (rmse) was small ($\mathrm{rmse} \leq 0.05$). These cells were referred to as Brownian-like. For the remaining curves, we varied the endpoint of the linear part to find the best fitting line satisfying $\mathrm{rsquare} \geq 0.95$ and $\mathrm{rmse} \leq 0.05$ and considered a measure of relative deviation from linearity:

$$\mathrm{RD_i} = \frac{1}{K}\sum_{k=1}^{K}\frac{\mathrm{msd_i(k\delta t)}}{4\hat{a}k\delta t + \hat{b}} \quad (3)$$

where $\hat{a}, \hat{b}$ are the estimated linear coefficients. Cells with corresponding values of $\mathrm{RD_i} > 1$ were considered superdiffusive, while $\mathrm{RD_i} < 1$ were considered subdiffusive.

Although the time-averaged mean squared displacement (2) has become widely used (Ruthardt et al., 2011), it is known that uncertainty in the curve increases with

increasing time-lag $k\delta t$ due to few remaining displacements. Thus, the recommended length to be analysed reliably is approximately $1/4$ of the total number of data points available (Saxton and Jacobson, 1997). However, in the case of our data the majority of normal cells died or divided early after plating leading to extremely few cells from the initial plated population to be observed after 12h postplating. We retained $2/5$ of the length of points available, representing approximately 5h at an acquisition rate of 1 frame every 10min. We classified the type of diffusion from the time-averaged msd and further analysed the cell movement characteristics from the ensemble average msd, which is a population measure and does not suffer from these statistical problems.

Motility analysis using fractional diffusion models

Assuming at time t postplating, a number of $N(t)$ surviving (but not divided) cells are found with corresponding coordinates $(x_i(t), y_i(t))$, the ensemble average mean squared displacement was computed as:

$$\mathrm{msd}(t) = \frac{1}{N(t)} \sum_{i=1}^{N(t)} \left[\left(x_i(t) - x_i(0)\right)^2 + \left(y_i(t) - y_i(0)\right)^2 \right]. \quad (4)$$

Given the two subpopulations of cells identified as Brownian-like and superdiffusive, we computed the msd (4) for each of these separately. To exclude remaining heterogeneity in the movement of each subpopulation, cells that had a contribution of over five times of that of remaining cells were excluded from the motility analysis. We analysed the msd behaviour up to 8h postplating, which coincides with division time of normal cells.

We fitted the msd data using a fractional diffusion model, the Klein-Kramers equation, which describes superdiffusive processes ($1 < \beta \leq 2$) and for $\beta = 1$ reduces to an Ornstein-Uhlenbeck model (Barkai and Silbey, 2000),(Metzler and Klafter, 2000).

Recent work in (Dieterich et al., 2008) has shown that the fractional Klein-Kramers equation can model not only the transition from stretched exponential to power law behaviour $msd \sim t^{\beta}$, for any $1 < \beta \leq 2$ but also characterize complex statistics such as the spread of cells over time as the spatial probability distribution function (pdf) more accurately than the standard Ornstein-Uhlenbeck model which assumes $\beta = 1$. A major advantage of both these models is that they allow characterizing the dynamics of cells exhibiting different types of movement, in different culture conditions with a reduced set of parameters.

We followed the approach proposed in (Dieterich et al., 2008) to model the mean squared displacement curves. The Ornstein-Uhlenbeck equation was used to model the msd for Brownian-like normal and adapted cells:

$$msd_{OU}(t) = 4\frac{{v_{th}}^2}{\gamma^2}(\gamma t - 1 + \exp(-\gamma t)) + (2\eta)^2 \quad (5)$$

where v_{th} , γ, η denote thermal speed, damping and standard deviation of noise respectively. The msd for superdiffusive normal and adapted cells was modelled with the fractional Klein-Kramers equation:

$$msd_{FKK}(t) = 4{v_{th}}^2 t^2 E_{\alpha,3}(-\gamma t^{\alpha}) + (2\eta)^2 \quad (6)$$

where:

$$E_{\alpha,3}(z) = \sum_{j=0}^{\infty} \frac{z^j}{\Gamma(\alpha + 3j)} \quad (7)$$

denotes the generalised Mittag-Leffler function and the exponent parameter $\alpha = 2 - \beta$. Using the Fox H function (Metzler and Klafter, 2000), (7) can be expressed as:

$$E_{\alpha,\delta}(z) = H_{1,2}^{1,1}\left[-z\middle|\begin{matrix}(0,1)\\(0,1),(1-\alpha,\delta)\end{matrix}\right] \tag{8}$$

In order to compute the Fox H function numerically, we first deduced that for $\delta = 3$ (8) can be expressed as a Meijer G function (Mathai and Hauboldt, 2008):

$$E_{\alpha,3}(z) = \frac{2\pi}{3^{\alpha-0.5}} G_{1,4}^{1,1}\left[-\frac{z}{27}\middle|\begin{matrix}0\\0, 1-\frac{\alpha}{3}, 1-\frac{\alpha+1}{3}, 1-\frac{\alpha+2}{3}\end{matrix}\right] \tag{9}$$

The Meijer G Function was computed in Matlab using the symbolic toolbox MuPAD.

For the Brownian-like normal and adapted cells, which are characterised by the Ornstein-Uhlenbeck model, the spatial probability distribution function (pdf) of a cell to travel the distance δx after time t was generated with the Gaussian distribution:

$$p_{OU}(\delta x, t) = \frac{1}{\sqrt{4\pi D_1 t}} \exp\left(\frac{-\delta x^2}{4D_1 t}\right) \tag{10}$$

where $D_1 = {}^{vth^2}/_{\gamma}$ denotes the generalised diffusion coefficient for $\alpha = 1$.

For the superdiffusive normal and adapted cells, the spatial pdf corresponding to the fractional Klein-Kramers model was generated as (Metzler and Klafter, 2000):

$$p_{FKK}(\delta x, t) = \frac{1}{\sqrt{4\pi D_\alpha t^{2-\alpha}}} H_{1,2}^{2,0}\left[\frac{\delta x^2}{4D_\alpha t^{2-\alpha}}\middle|\begin{matrix}(\alpha/2, 2-\alpha)\\(0,1),(0.5,1)\end{matrix}\right] \tag{11}$$

We deduced the transformation from Fox H function to Meijer G function by assuming $\beta = 2 - \alpha$ is a rational number $\beta = {}^{k}/_{l}, k, l \in \mathbb{Z}^*$ (Mathai and Hauboldt, 2008):

$$H_{1,2}^{2,0}\left[z\middle|\begin{matrix}(\alpha/2, 2-\alpha)\\(0,1),(0.5,1)\end{matrix}\right] =$$

$$= \frac{(2\pi)^{\frac{k-2l+1}{2}} l^{\frac{1}{2}}}{k^{\frac{l-k}{2l}}} G_{k,2l}^{2l,0}\left[\frac{z^l k^k}{l^l}\middle|\begin{matrix}-\frac{1}{2l}+\frac{1}{k}, -\frac{1}{2l}+\frac{2}{k}, \dots, -\frac{1}{2l}+1\\\left(0, \frac{1}{l}, \dots, \frac{l-1}{l}\right), \left(\frac{1}{2l}, \frac{1}{2l}+\frac{1}{l}, \dots, \frac{1}{2l}+\frac{l-1}{l}\right)\end{matrix}\right] \tag{12}$$

The overall pdf for the motile normal and adapted cell cultures over time is computed as an equally weighted mixture with known mixing components $n_1, n_2 = 0.5$:

$$p(\delta x, t) = n_1 p_{OU}(\delta x, t) + n_2 p_{FKK}(\delta x, t) \tag{13}$$

Where p_{OU}, p_{FKK} are the pdfs for Brownian-like cells (parameters estimated with the Ornstein-Uhlenbeck model) and superdiffusive cells (parameters estimated with the fractional Klein-Kramers models) respectively.

Model parameters were estimated using the trust region reflective algorithm for nonlinear least squares fitting with boundary constraint > 0 for all parameters and in addition $\alpha \in (0,2)$. Nonlinear regression functions from the Statistics Toolbox were used to compute standard deviations for the parameters and 95% model prediction bounds. The model parameter estimates are included in **Table S1** and **Table S2**.

To calculate the distance that 95% of cells can travel up to with respect to the point of origin we considered the cumulative distribution function (cdf), associated to the pdf, which measures the probability that cells may be observed at distances ≤ x. Thus, the 95% maximum distance satisfies $\mathrm{cdf}(x, t) = 0.95$.

All data extraction, visualisations and algorithms were implemented in Matlab R2010b. For further information please contact the corresponding author at d.coca@sheffield.ac.uk.

Supplemental References

Amit, M., Carpenter, M.K., Inokuma, M.S., Chiu, C.P., Harris, C.P., Waknitz, M.A., Itskovitz-Eldor, J., and Thomson, J.A. (2000). Clonally derived human embryonic stem cell lines maintain pluripotency and proliferative potential for prolonged periods of culture. Dev Biol 227, 271-278.

Barkai, E., and Silbey, R.J. (2000). Fractional Kramers Equation. The Journal of Physical Chemistry B 104, 3866-3874.

Cremers, D., Rousson, M., and Deriche, R. (2007). A Review of Statistical Approaches to Level Set Segmentation: Integrating Color, Texture, Motion and Shape. Int J Comput Vision 72, 195-215.

Kohler, G., and Milstein, C. (1975). Continuous cultures of fused cells secreting antibody of predefined specificity. Nature 256, 495-497.

Kusumi, A., Sako, Y., and Yamamoto, M. (1993). Confined lateral diffusion of membrane receptors as studied by single particle tracking (nanovid microscopy). Effects of calcium-induced differentiation in cultured epithelial cells. Biophys J 65, 2021-2040.

Mathai, A.M., Hauboldt, H.J. (2008). Special functions for applied scientists (New York, NY: Springer Science and Business Media).

Qian, H., Sheetz, M.P., and Elson, E.L. (1991). Single particle tracking. Analysis of diffusion and flow in two-dimensional systems. Biophys J 60, 910-921.

Ruthardt, N., Lamb, D.C., and Brauchle, C. (2011). Single-particle Tracking as a Quantitative Microscopy-based Approach to Unravel Cell Entry Mechanisms of Viruses and Pharmaceutical Nanoparticles. Mol Ther 19, 1199-1211.

Saxton, M.J., and Jacobson, K. (1997). Single-particle tracking: applications to membrane dynamics. Annu Rev Biophys Biomolec Struct 26, 373-399.

Shevinsky, L.H., Knowles, B.B., Damjanov, I., and Solter, D. (1982). Monoclonal antobody to murine embryos defines a stage-specific embryonic antigen expressed on mouse embryos and human teratocarcinoma cell. Cell 30, 697-705.
